# Consistency-Aware Weakly Supervised Anomaly Sensing for Large-Scale Expressway ETC Gantry Transactions

**DOI:** 10.3390/s26185889

**Published:** 2026-09-17

**Authors:** Yijia Li, Haiyan Jiang, Xiaoxue Xu, Vladimir Zyryanov

**Affiliations:** 1 Department of Unmanned Aerial Vehicles, School of Aeronautics, Shandong Jiaotong University, Haitang Road No. 5001, Jinan 250357, China; 220160@sdjtu.edu.cn (Y.L.); 13082716062@163.com (X.X.); 2 Department of Traffic Engineering, School of Transportation and Logistics Engineering, Shandong Jiaotong University, Haitang Road No. 5001, Jinan 250357, China; 3 Department of Traffic and Transport Organization, Faculty of Road Traffic, Don State Technical University, Sotsialisticheskaya Street No. 162, 344022 Rostov-on-Don, Russia; tolbaga@mail.ru

**Keywords:** electronic toll collection, ETC gantry transactions, anomaly sensing, weak supervision, LightGBM

## Abstract

Electronic toll collection (ETC) gantries generate transaction records, yet existing anomaly-detection approaches often depend on manual labels or external information, limiting multiview inconsistency ranking under restricted supervision. We propose consistency-aware weakly supervised anomaly sensing for ETC (CAWS-ETC), combining monetary, temporal, structural-pattern, and contextual-semantic evidence and transferring high-confidence references to Light Gradient Boosting Machine (LightGBM). Evaluation used 14,510,847 transactions from 2525 gantries. On joint-transfer benchmarks, CAWS-ETC achieved area under the precision–recall curve (AUPRC) values of 0.9354 for rule-aligned interventions and 0.9018 for rule-orthogonal challenges, versus 0.7609 and 0.7377 for Isolation Forest. A blinded audit of 1200 unmodified transactions by two independent reviewers yielded 777 determinate labels; CAWS-ETC achieved a sampling-weighted AUPRC of 0.7946, while Isolation Forest showed higher ranking point estimates on this temporal-only subset. Post hoc attribution showed that direct rule-created and high-confidence probabilistic labels were identical after the 0.90/0.10 selection, and matched LightGBM models produced essentially identical rankings. Thus, capability beyond direct rule activation arose primarily from discriminative transfer rather than measurable gains from probabilistic aggregation or posterior-confidence weighting. Because all experiments used one day from one provincial network, multi-day, seasonal, and cross-region generalizability remain unverified.

## 1. Introduction

Electronic toll collection (ETC) systems have evolved from automated payment facilities into distributed roadside sensing infrastructures. Each gantry passage can generate structured information on transaction time, toll calculation, vehicle category, transaction medium, and related operational states. When such records are collected across an expressway network, they can support traffic state estimation, travel time analysis, vehicle arrival prediction, infrastructure positioning, and other intelligent transportation applications [1,2,3]. The analytical value of these applications, however, depends on the integrity of the underlying transaction records. Inaccurate timestamps, inconsistent fee components, missing arrays, conflicting vehicle codes, or unusual combinations of transaction attributes can propagate into downstream estimates and operational decisions.

Existing ETC data studies have addressed several aspects of this reliability problem. Missing records have been reconstructed using temporal, spatial, and multi-attribute correlations [4]. Dynamic time-warping and supervised tree-based models have also been applied to detect predefined abnormal ETC patterns [5,6]. These studies demonstrate that transaction abnormalities can be identified when the expected route structure, external capture data, or representative abnormal classes are available. Nevertheless, operational ETC databases often do not contain manually adjudicated anomaly labels, and access to supplementary topology, trajectory, image, or plate recognition data may be restricted. Under such conditions, a record can be inconsistent without belonging to a previously defined failure class, while an unusual field value can still be legitimate within a particular vehicle, medium, or transaction context.

From a machine learning perspective, ETC transaction auditing is an anomaly-detection problem. Anomaly detection aims to identify observations that deviate from an expected data-generating pattern, but the definition of abnormality is inherently dependent on the application, available supervision, and structure of the data [7,8]. Existing methods include isolation-based models, one-class learning, reconstruction models, representation learning, and self-supervised transformation methods [7,8,9,10,11,12]. Although these approaches have substantially expanded the available methodological repertoire, comparative studies have shown that no single detector consistently dominates across anomaly types, supervision regimes, and data corruptions [10,11]. This limitation is particularly relevant to ETC transactions, in which deterministic accounting relations coexist with contextual temporal deviations, categorical conflicts, recurring missingness, and rare but potentially legitimate operating states.

Weak supervision provides a possible bridge between rigid business rules and fully supervised anomaly classification. Instead of assigning definitive labels to individual records, domain knowledge can be represented through labeling functions that emit noisy normal or anomaly evidence and abstain when their conditions are not satisfied. A probabilistic model can then estimate the reliability of these sources and combine their outputs into weak training labels [13]. This paradigm has been increasingly considered in domains where expert knowledge is available but manually verified labels are scarce or costly [14]. However, directly applying weak rules is insufficient when the objective is to rank subtle deviations that do not cross a predetermined threshold. The weak evidence must be transferred to a discriminative model that can learn continuous feature effects and interactions while remaining computationally feasible for large transaction tables.

In this study, a transaction anomaly is defined as a record that violates a deterministic field relationship, exhibits a statistically implausible deviation from comparable transactions, or contains an unusual combination of transaction attributes. This definition concerns transaction data consistency rather than confirmed fraud, toll evasion, equipment failure, or legal wrongdoing. On this basis, this study develops CAWS-ETC, a consistency-aware weakly supervised anomaly-sensing framework for ETC gantry transactions. The framework represents each transaction through monetary, temporal, structural-pattern, and contextual–semantic consistency views. Abstaining weak sources are integrated through a probabilistic latent-class model, after which high-confidence weak labels are transferred to a weighted Light Gradient Boosting Machine (LightGBM) discriminator. LightGBM was selected because tree boosting can efficiently model nonlinear interactions, missing values, heterogeneous variables, and large tabular datasets [15]. The resulting output is treated as an anomaly-ranking score rather than a calibrated probability of a specific operational cause.

The main contributions of this study are summarized as follows:

1. We formulate ETC anomaly sensing as a four-view transaction consistency problem combining monetary, temporal, structural-pattern, and contextual–semantic evidence, while excluding direct identifiers and treating the output as an anomaly-ranking signal rather than a causal diagnosis.
2. We develop CAWS-ETC, which integrates abstaining programmatic consistency sources and transfers high-confidence references to LightGBM. A matched direct-rule LightGBM comparator is included to distinguish discriminative transfer from any incremental contribution of probabilistic aggregation.
3. We establish a leakage-resistant multi-evidence validation protocol combining held-out time and gantry evaluation, rule-aligned and rule-orthogonal challenges, blinded adjudication of unmodified transactions by two independent reviewers, and operational proxies excluded from model construction.
4. We evaluate robustness, interpretability, and scalability through ablation, sensitivity analysis, tree-contribution analysis, low-prevalence testing, and privacy-preserving gantry–time aggregation. On the controlled benchmarks, CAWS-ETC achieved joint-transfer area under the precision–recall curve (AUPRC) values of 0.9354 for rule-aligned interventions and 0.9018 for rule-orthogonal challenges, compared with 0.7609 and 0.7377, respectively, for Isolation Forest.

The experiments indicate that CAWS-ETC maintained similar performance when evaluated on later within-day periods, held-out gantries, and their joint combination. Its AUPRC ranged from 0.9354 to 0.9404 on rule-aligned interventions and from 0.9014 to 0.9023 on the rule-orthogonal challenge. The blinded manual audit provided the principal real-record validation: CAWS-ETC achieved a sampling-weighted AUPRC of 0.7946 against 777 determinate manual labels, while Isolation Forest produced higher ranking point estimates on this temporal-only manually adjudicated subset. The subsequent post hoc supervision-source attribution analysis refined the interpretation of the rule-orthogonal results. After the original 0.90/0.10 posterior selection, high-confidence probabilistic labels were exactly equivalent to direct rule-created labels in training eligibility and binary class. Under matched records and model capacity, direct-rule LightGBM and the matched confidence-weighted LightGBM produced essentially identical rankings. These results indicate that the ability to rank deviations beyond direct rule activation arose primarily from discriminative transfer of programmatic consistency supervision, whereas no measurable incremental ranking benefit from probabilistic aggregation or posterior-confidence weighting was identified in the present dataset.

The remainder of this paper is organized as follows. Section 2 reviews ETC transaction analysis, anomaly-detection methods, weak supervision, and the methodological gap addressed in this study. Section 3 describes the data preparation, multi-view consistency features, probabilistic weak-label learning, discriminative ranking model, and validation protocol. Section 4 presents the data-audit, detection, transferability, robustness, interpretability, and computational results. Section 5 discusses the methodological and operational implications and limitations. Section 6 concludes the paper and outlines directions for further research.

## 2. Literature Review

### 2.1. ETC Gantry Transaction Data and Anomaly Detection

ETC infrastructure records a sequence of discrete vehicle–gantry transaction events and therefore provides a different type of sensing information from continuously sampled global positioning system (GPS), loop detector, radar, or video data. The records are typically sparse in physical space but rich in transaction context, including passage time, vehicle category, transaction medium, fee calculation, and route-related identifiers. Consequently, previous ETC research can be grouped into three broad categories: traffic-state and travel time inference, transaction data restoration, and abnormal-pattern detection. This functional expansion has transformed ETC data from a toll settlement resource into a source of information for broader intelligent transportation applications [1].

The first category uses ETC passage events to infer traffic conditions between gantries. Zou et al. [2] combined ETC records with spatial–temporal modeling to estimate expressway speeds, while Lai et al. [3] used ETC observations to estimate downstream vehicle arrival times. Related studies have reconstructed vehicle travel processes, classified traffic states, and inferred section-level conditions from successive gantry observations. These applications demonstrate the value of ETC data when a consistent relationship exists among passage time, spatial location, and the sequence of observations. Their reliability nevertheless depends on valid timestamps, correctly associated gantries, and complete transaction records. A record-level inconsistency can therefore affect both the transaction itself and any traffic variable derived from it.

The second category focuses on missing and incomplete ETC information. Zou et al. [4] developed a missing-data restoration model using multi-attribute features, illustrating that ETC fields contain exploitable dependencies across time, space, vehicle attributes, and transaction context. Restoration methods are appropriate when the missing component and its expected counterpart can be clearly defined. They do not, however, fully address records that are syntactically complete but internally inconsistent. Examples include agreement failures between total and component fees, validly formatted but implausible timestamps, disagreements between vehicle code fields, or combinations that are rare only under a specific transaction context. Such conditions require an anomaly formulation broader than missing-value imputation.

The third category detects predefined abnormal ETC patterns. Guo et al. [5] used an improved dynamic time-warping (DTW) procedure to compare ETC transaction sequences and identify abnormal data rapidly. Zou et al. [6] integrated transfer learning and Extreme Gradient Boosting (XGBoost) for ETC transaction anomaly detection, using road network topology and capture information to derive abnormal patterns. These studies show that domain structure can substantially improve detection compared with generic unsupervised modeling. Their target, however, is generally determined through predefined abnormal trajectories, supplementary sensing, or labeled source tasks. Such assumptions are not always satisfied in restricted operational datasets that contain only transaction table attributes and no manually verified anomaly classes.

A further difficulty is that an ETC field cannot always be classified as normal or anomalous in isolation. A missing fee array may be legitimate for one transaction medium but suspicious for another. A disagreement between recorded and billing vehicle types may reflect an operational correction, an unusual vehicle class, or a data inconsistency. Similarly, a long elapsed time can indicate a valid extended trip, a timestamp placeholder, or an abnormal transaction sequence. Therefore, a high-level anomaly-sensing framework should distinguish deterministic contradictions from context-dependent deviations and should allow a knowledge source to abstain when its prerequisites are absent.

The ETC literature has consequently established three important foundations for the present study. First, ETC records contain useful temporal, monetary, vehicle, and operational dependencies. Second, domain-informed models are more meaningful than treating all fields as independent numerical variables. Third, data quality must be evaluated before ETC records are used for downstream traffic analysis. Nevertheless, existing studies provide limited guidance on how heterogeneous consistency relations can be combined when manual labels and supplementary sensing modalities are unavailable. CAWS-ETC is designed for this setting by integrating monetary, temporal, structural-pattern, and contextual-semantic consistency evidence directly from transaction-table data, without requiring manually adjudicated labels or supplementary sensing modalities during model development.

### 2.2. Weak Supervision and Consistency-Aware Anomaly Learning

Anomaly-detection methods are commonly categorized according to their representation of normality and the amount of supervision available. Isolation-based approaches separate unusual records through short partition paths; one-class methods estimate a boundary around normal observations; reconstruction methods identify records that cannot be accurately reproduced; and deep representation methods learn latent structures in which anomalous samples are expected to remain distinguishable [7,8,9,10]. Self-supervised approaches further construct surrogate transformations or pretext tasks to learn anomaly-sensitive representations without manually supplied class labels [12]. These methods have achieved strong results in many domains, but their performance remains dependent on the anomaly mechanism, contamination level, feature representation, and evaluation protocol.

For structured ETC tables, unsupervised methods offer an attractive baseline because they do not require anomaly labels. Isolation Forest, for example, is computationally efficient and can process mixed tabular representations after suitable feature construction [16]. However, an unsupervised model has no direct knowledge that a recorded fee should equal the sum of its components, that one timestamp should not occur after another, or that the meaning of a missing field depends on the transaction medium. It may therefore assign high scores to statistically rare but valid records while overlooking contradictions that are operationally important but numerically small. Benchmark studies further indicate that increased supervision, even when limited, can materially change anomaly-detection performance and that algorithm rankings vary across anomaly types [11].

Conventional supervised learning can incorporate operational knowledge through labeled examples, but manually adjudicating ETC anomalies is expensive and conceptually difficult. A reviewer must often consult multiple fields, fee-calculation logic, temporal relations, and operational context before deciding whether a record is genuinely inconsistent. Labels can also be incomplete because only recognized failure mechanisms are documented. Treating system-generated status codes as definitive ground truth introduces another risk: the classifier may reproduce the logic of those codes rather than learn a broader transaction-consistency construct.

Weak supervision addresses this setting by encoding multiple imperfect sources of knowledge instead of requiring one authoritative label. Ratner et al. [13] formalized labeling functions as programmatic sources that can express heuristics and abstain outside their domains of competence. Their outputs can be combined through a generative model that estimates source accuracy and dependence without direct access to ground-truth labels. More recent reviews have shown that weak supervision is particularly relevant to industrial monitoring and predictive maintenance problems, where domain rules and historical operating knowledge are available but verified fault labels remain sparse [14].

Weak supervision nevertheless introduces several methodological requirements. First, weak sources should express distinct and interpretable relationships rather than duplicate the same heuristic under different names. Second, normal evidence must be conservative: satisfaction of one field relation does not imply that the entire transaction is normal. Third, source correlations can bias posterior probabilities when conditional independence is only approximate. Fourth, a probabilistic weak-label model can combine rule outputs but cannot rank records that share the same weak-source pattern. A secondary discriminative model is therefore required when the objective includes continuous ranking and generalization to subthreshold deviations. Whether probabilistic aggregation and posterior-confidence weighting provide an incremental benefit once the same discriminator is trained directly on rule-created labels is therefore an empirical component-attribution question rather than an assumption of the proposed framework.

Gradient-boosted decision trees are appropriate for this transfer because they model nonlinear thresholds and interactions while remaining efficient on large tabular data. LightGBM improves the computational efficiency of gradient boosting through optimized sampling and feature handling and has been widely adopted for classification and ranking tasks [15]. Its native handling of missing numerical values and its compatibility with sample weights are useful when high-confidence weak labels have unequal reliability. Tree-based models can also be interpreted through additive contribution methods. Lundberg et al. [17] showed that tree explanations can connect local predictions to global feature effects, enabling an audit of whether the model relies on plausible consistency attributes rather than excluded identifiers or operational outcomes.

The present study differs from conventional weak-label classification in two respects. First, the weak sources are organized around four consistency views rather than around alternative annotators of one predefined anomaly class. Second, the final output is deliberately interpreted as a ranking score. The probabilistic model transfers domain evidence to the discriminator, but the score is not treated as a calibrated probability of fraud or equipment failure. This distinction is essential because weak labels inherit the scope and limitations of the rules from which they are constructed.

### 2.3. Research Gap and Methodological Positioning

The literature reveals three unresolved gaps. The first concerns the target construct. Existing ETC studies commonly focus on missing records, trajectory or path abnormalities, toll evasion, or named operational failure types. These are important tasks, but they do not cover the broader situation in which a transaction exhibits internal or contextual inconsistency without a confirmed causal label. A method designed for this setting must rank heterogeneous inconsistency evidence while avoiding unsupported conclusions about why the record occurred.

The second gap concerns the supervision mechanism. Generic unsupervised detectors do not incorporate known monetary, temporal, and semantic relationships, whereas supervised ETC models require abnormal examples or additional data sources. Hard rules incorporate domain knowledge but produce discontinuous binary decisions and cannot directly rank subthreshold or multiview deviations. A probabilistic weak-supervision layer can integrate multiple sources and represent source-level uncertainty, but its incremental value over a discriminative model trained directly on rule-created labels is not guaranteed. The unresolved methodological problem therefore has two parts: whether programmatic consistency supervision can be transferred into a continuous ranking model and whether probabilistic aggregation provides additional ranking value beyond this discriminative transfer.

The third gap concerns validation under label scarcity. Anomaly-detection evaluation is sensitive to class imbalance, metric selection, synthetic design, and the definition of an anomalous event [18,19,20]. The precision–recall curve is more informative than the receiver operating characteristic curve when the positive class is rare because it directly reflects the trade-off between identifying anomalies and controlling false alerts [18]. Studies of time-series anomaly detection have also shown that favorable scoring protocols can overestimate performance and alter method rankings [19,20]. Therefore, a single synthetic test or operational status field is insufficient to establish credible performance.

CAWS-ETC is positioned to address these gaps through a layered validation design. Rule-aligned interventions verify that the implemented pipeline recovers known consistency mechanisms, but they are interpreted only as positive controls. Held-out gantries and later within-day periods evaluate transfer without mixing contextual reference statistics across partitions. System-recorded transaction outcomes are excluded from all training stages and used only to measure independent association and high-score enrichment. Most importantly, rule-orthogonal challenges keep the primary weak-source state unchanged and test whether the discriminative model has learned continuous and multiview information beyond direct rule activation. AUPRC is emphasized for ranking under imbalance, while the area under the receiver operating characteristic curve (AUROC), precision, recall, F1-score, low-prevalence stress tests, ablation, sensitivity, and tree contributions provide complementary evidence.

Accordingly, the methodological position of this study is neither purely rule-based, fully supervised, nor conventionally unsupervised. It is a domain-informed weak-supervision approach developed under restricted information and without manually adjudicated labels during model construction. Manual labels were introduced only after the original CAWS-ETC development and pre-specified evaluation pipeline had been frozen. They were used for independent evaluation of unmodified held-out transactions and were later reused only as an evaluation set for the post hoc supervision-source attribution analysis. Controlled interventions assess mechanism recovery, manual adjudication assesses observable real-record inconsistencies, operational proxies assess association rather than ground-truth accuracy, and the final score expresses consistency-based anomaly evidence rather than causal certainty.

Recent studies published in 2026 further illustrate both the progress and the remaining methodological gap. Taheri Sarteshnizi et al. [21] systematically examined time-series anomaly detection methods on real-world traffic-volume data and introduced a visually inspected anomaly-labeled dataset, demonstrating the importance of domain-adapted anomaly definitions and rigorous real-data evaluation; however, their setting focuses on traffic-volume time series rather than heterogeneous ETC transaction tables and domain-specific monetary, structural, and semantic consistency relations. Wang et al. [22] extended weakly supervised tabular anomaly detection to cell-level localization, improving the interpretability of anomalous samples under limited supervision, although the method still assumes access to coarse sample-level anomaly labels. These recent developments reinforce the need for anomaly-ranking methods that can exploit domain knowledge when manually verified labels and supplementary sensing information are unavailable. Taken together, the principal advantage of CAWS-ETC in the setting considered here is its ability to combine transaction-table-only domain consistency knowledge with continuous anomaly ranking and leakage-resistant multi-evidence validation, without requiring manual labels during model development or supplementary sensing modalities. This advantage is methodological and setting-specific rather than a claim of universal detector superiority. To provide a structured comparison of the recent literature and make the methodological positioning more transparent, Table 1 summarizes representative studies published from 2021 to 2026 in terms of their main focus, principal strengths, and limitations relevant to the present study.

## 3. Materials and Methods

**Figure 1:**
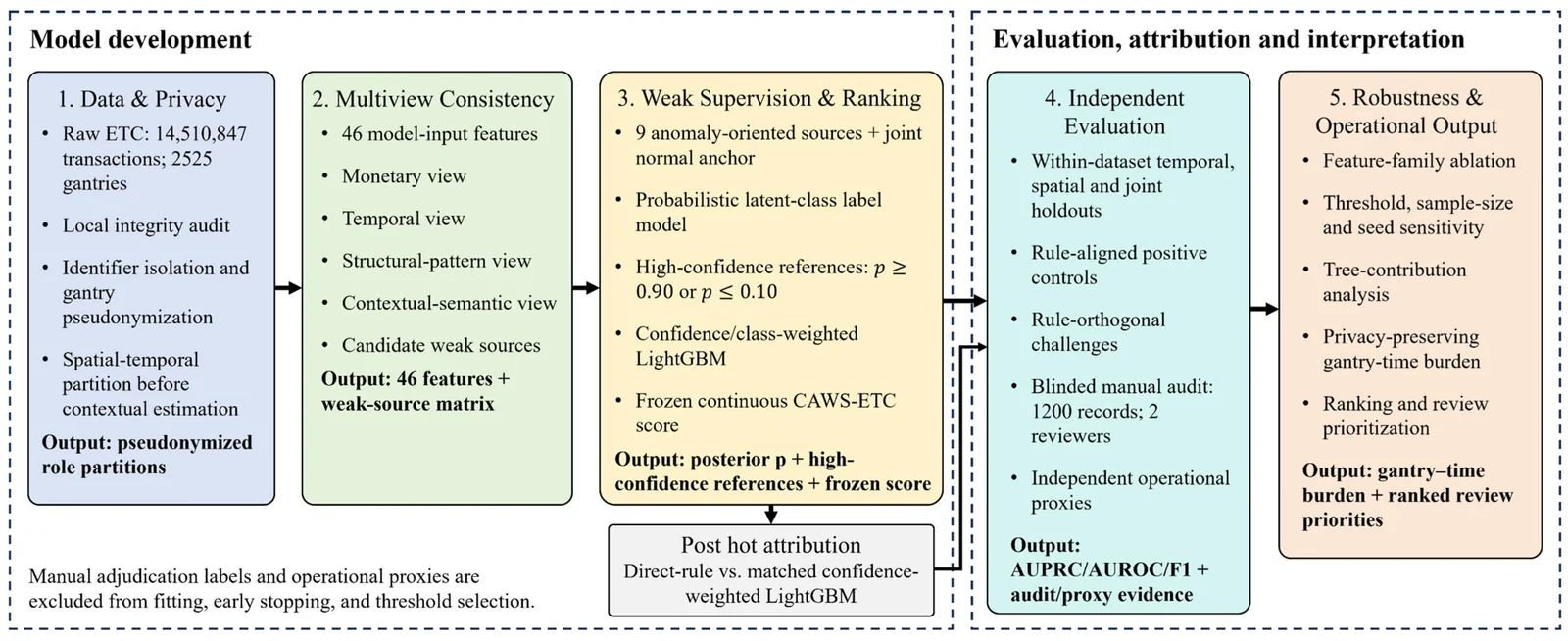
Detailed analytical workflow of CAWS-ETC, with the principal output of each stage identified below the corresponding processing block.

The CAWS-ETC workflow is organized into five linked stages, as summarized in Figure 1. Stage 1 performs local integrity auditing, privacy-preserving preprocessing, and spatial–temporal partitioning before contextual statistics are estimated. Stage 2 constructs the 46 monetary, temporal, structural-pattern, and contextual–semantic features and the associated candidate weak sources. Stage 3 integrates the selected weak sources through the probabilistic latent-class model and transfers the resulting high-confidence references to the weighted LightGBM discriminator. Stage 4 evaluates the frozen model using within-dataset temporal, spatial, and joint holdouts, rule-aligned and rule-orthogonal challenges, blinded manual adjudication, and independent operational proxies. Stage 5 examines robustness, interpretability, and privacy-preserving gantry–time aggregation. The post hoc supervision-source attribution analysis is shown separately because it does not modify the originally fitted CAWS-ETC model. Figure 1 provides a stage-level overview, while Algorithm 1 summarizes the corresponding procedural sequence and explicitly separates model-development steps from evaluation-only procedures; Section 3.1, Section 3.2, Section 3.3 and Section 3.4 provide the full technical definitions. For easier workflow tracing, Figure 1 also identifies the principal output passed forward from each stage, while the corresponding parameters and technical definitions are detailed in Section 3.1, Section 3.2, Section 3.3 and Section 3.4.
**Algorithm 1.** CAWS-ETC development, scoring, and evaluation workflow**Input:** Raw ETC transaction table D; fixed random seed 20,221,213. **Output:** Transaction-level CAWS-ETC anomaly-ranking scores, gantry–time anomaly burdens, and evaluation results.**Stage 1: Data preparation and privacy preservation**1.Audit the source schema, duplicate identifiers, timestamp validity, monetary-component consistency, and component-array integrity.2.Isolate direct identifiers, pseudonymize gantry identifiers, and exclude operational-status proxy fields from model development.3.Partition gantries and observation periods into training, validation, and test roles before estimating any context-dependent reference statistics.**Stage 2: Four-view consistency representation**4.Estimate contextual medians, median absolute deviations, categorical frequencies, and missingness-pattern frequencies using train_core only, and freeze these references.5.Construct the 46 monetary, temporal, structural-pattern, and contextual–semantic model-input features.6.Generate candidate weak sources and retain the nine active anomaly-oriented sources and the joint normal anchor.**Stage 3: Weak supervision and discriminative ranking**7.Fit the probabilistic latent-class model on the deterministic 500,000-record train_core sample and compute weak-label posterior probabilities.8.Select high-confidence anomaly references with *p* ≥ 0.90 and normal references with *p* ≤ 0.10; exclude intermediate-probability records from discriminator training.9.Select 180,000 anomaly and 420,000 normal training records, assign confidence and class weights, and fit the LightGBM discriminator using validation_joint for early stopping.10.Freeze the fitted discriminator and generate continuous CAWS-ETC anomaly-ranking scores for the required datasets.**Stage 4: Independent evaluation and attribution**11.Evaluate the frozen model using within-dataset temporal, spatial, and joint holdouts, rule-aligned positive controls, rule-orthogonal challenges, blinded manual adjudication, and independent operational proxies.12.Keep manual labels and operational proxies evaluation-only and exclude them from fitting, early stopping, and threshold selection.13.Conduct the post hoc supervision-source attribution analysis using direct-rule LightGBM and matched confidence-weighted LightGBM without modifying the originally fitted CAWS-ETC model.**Stage 5: Robustness, interpretation, and operational aggregation**14.Perform feature-family ablation, threshold/sample-size/seed sensitivity analyses, and tree-contribution interpretation.15.Aggregate transaction scores into privacy-preserving, pseudonymized gantry–time anomaly burdens for operational prioritization.

### 3.1. Data Preparation and Problem Formulation

The empirical data used in this study consist of electronic toll collection (ETC) gantry transaction records collected across the Shandong expressway network on 13 December 2022. The original semicolon-delimited file contained 14,510,847 transaction records from 2525 gantries over a complete 24 h observation period. Each row represents a vehicle–gantry transaction event rather than a continuously sampled vehicle trajectory. The 24 source fields describe transaction and passage identifiers, gantry attributes, transaction and entry times, payable and discounted fees, toll-interval components, transaction media, vehicle type and class, previous-gantry information, and operational status. During the development of the originally fitted CAWS-ETC and the four pre-specified comparator methods, no road-network topology, image data, vehicle speed measurements, manually verified anomaly labels, or records from other dates were available for feature construction, weak-label generation, model fitting, early stopping, or threshold selection. Manual adjudication was introduced only after these original model-development and threshold-selection procedures had been frozen. The resulting manual labels were subsequently reused only as an evaluation set for the post hoc supervision-source attribution models described in Section 3.4 and were not used for their fitting, model-capacity selection, or tuning. The 24 source fields were grouped according to their analytical roles, including transaction identifiers, gantry information, temporal attributes, monetary and component-array attributes, vehicle and transaction context, and operational-status indicators. Table 2 summarizes the representative fields in each group and specifies whether they were retained as model inputs, used only for local integrity checks, or reserved exclusively for independent evaluation.

A local two-pass quality control procedure was applied before feature construction. The source file was first scanned sequentially to verify the expected field structure without loading the complete dataset into memory. The observed header exactly matched the 24-field schema, and no malformed rows, duplicated column names, or rows containing surplus values were detected. A subsequent columnar audit confirmed 14,510,847 distinct transaction identifiers and the same number of distinct row hashes; thus, neither duplicated transaction identifiers nor fully duplicated records were present. The transaction timestamp covered the entire observation day, from 00:00:00 to 23:59:59, and all three timestamp fields were syntactically parseable.

The raw records were then converted into a compressed Parquet representation and subjected to refined integrity checks. Parseability alone was not treated as evidence of temporal validity. Entry and previous-gantry timestamps were considered plausible only when they were no earlier than 1 January 2000, did not exceed the observation period, and did not occur after the corresponding transaction timestamp. Under these criteria, 197,650 entry timestamps and 2,599,253 previous-gantry timestamps preceded the plausibility boundary; 438 and 706 timestamps, respectively, exceeded the observation period; and 475 entry timestamps and 986 previous-gantry timestamps occurred after the corresponding transaction time. These records were retained and represented through validity indicators rather than deleted, because invalid or placeholder timestamps constitute potentially informative evidence of inconsistency.

Monetary component strings were separated using the vertical-bar delimiter and converted into component counts and sums. To distinguish the total amounts stored in each transaction from their component-level values, three monetary residuals were calculated for transaction i:

(1) $$r_{i}^{pay}=F_{i}^{pay}-\sum_{k=1}^{K_{i}^{pay}}f_{ik}^{pay}$$

(2) $$r_{i}^{discount}=F_{i}^{discount}-\sum_{k=1}^{K_{i}^{discount}}f_{ik}^{discount}$$

(3) $$r_{i}^{net}=\left(F_{i}^{pay}-F_{i}^{discount}\right)-\sum_{k=1}^{K_{i}^{net}}f_{ik}^{net}$$

where $F_{i}^{pay}$ and $F_{i}^{discount}$ denote the recorded total payable and discount amounts for transaction i, respectively; $f_{ik}^{pay}$, $f_{ik}^{discount}$, and $f_{ik}^{net}$ denote the k-th payable, discount, and net-fee component; and $K_{i}^{pay}$, $K_{i}^{discount}$, and $K_{i}^{net}$ are the corresponding numbers of component values. A residual equal to zero indicates exact agreement between the recorded total and its component representation. The refined audit identified 78,409 payable-total mismatches, 8620 discount-total mismatches, and 78,203 net-total mismatches. No inconsistency was found among the lengths of the toll-interval and monetary component arrays. Missing component arrays, negative monetary values, and differences between the recorded and billing vehicle types were not removed or automatically labeled as anomalies. Their frequencies varied substantially across transaction media and operational states, indicating that they should be treated as context-dependent evidence rather than universal errors. The principal integrity checks, their operational definitions, and the observed numbers of affected records are summarized in Table 3. The table distinguishes deterministic inconsistencies from context-dependent conditions that were retained as analytical attributes rather than being automatically removed.

Direct identifiers were isolated from the analytical workflow. Transaction identifiers were used only for local duplicate checks, after which raw vehicle plates, transaction and passage identifiers, raw gantry identifiers, and gantry hexadecimal codes were excluded from the model-development file. Gantry identifiers were replaced with stable pseudonyms generated using a locally stored random salt, and only the non-identifying plate color suffix was retained. The random salt and private mapping table remained on the local computer. The fields trade_result, fee_calc_result, and special_type were also excluded from feature-reference estimation and weak-label generation. They were reserved as independent operational proxies for later evaluation, thereby preventing system-recorded outcomes from leaking into the anomaly score. Missing numerical attributes were retained rather than deterministically imputed, and categorical levels absent from the training reference were mapped to a dedicated unknown category during model application.

After privacy-preserving preprocessing, transaction i was represented by a multi-view analytical vector:

(4) $$x_{i}=\left[x_{i}^{M},x_{i}^{T},x_{i}^{S},x_{i}^{C}\right]$$

where $x_{i}^{M}$, $x_{i}^{T}$, $x_{i}^{S}$, and $x_{i}^{C}$ contain monetary, temporal, structural, and contextual–semantic attributes, respectively. The complete vector contained 46 model-input variables. A transaction anomaly is defined as a record that violates a deterministic field relationship, exhibits a statistically implausible deviation from comparable transactions, or contains an unusual combination of transaction attributes. Let $y_{i}\in \left\{0,\;1\right\}$ denote the unobserved anomaly state of transaction i, where 1 represents an anomalous transaction and 0 represents a normal transaction. Because manually adjudicated labels were unavailable during model development, $y_{i}$ could not be observed for feature construction, weak supervision, discriminator training, early stopping, or threshold selection. A manually adjudicated subset was subsequently constructed only after the original CAWS-ETC development and pre-specified evaluation procedures had been frozen. These labels were used for independent real-record evaluation and were later reused only as an evaluation set for the post hoc supervision-source attribution analysis described in Section 3.4. The analytical objective was therefore to construct a continuous consistency-based ranking score, whose construction is described in Section 3.2 and Section 3.3. The score was designed to express the strength of anomaly evidence rather than the calibrated probability of fraud, toll evasion, equipment failure, or legal wrongdoing.

### 3.2. Multi-View Consistency Modeling and Weak Label Generation

Each transaction was represented through four complementary consistency views. The monetary view comprised residuals between recorded totals and component sums, component availability, parsing status, fee magnitudes, and the discount ratio. The temporal view comprised timestamp-validity indicators, entry-to-transaction and previous-gantry-to-transaction durations, and their contextual deviations. The structural-pattern view described the availability, number, and alignment of the toll-interval and monetary component arrays, together with recurring missingness and validity patterns. The contextual-semantic view described transaction medium, original and billing vehicle types, vehicle class, plate color, and the empirical frequencies of vehicle-code combinations. These four views produced 46 model-input variables; the operational proxy fields defined in Section 3.1 were excluded from the feature set.

For records satisfying the timestamp-validity conditions described in Section 3.1, two elapsed-time attributes were calculated:

(5) $$∆t_{i}^{entry}=t_{i}^{trans}-t_{i}^{entry}$$

(6) $$∆t_{i}^{previous}=t_{i}^{trans}-t_{i}^{previous}$$

where $t_{i}^{trans}$ is the transaction timestamp, $t_{i}^{entry}$ is the recorded expressway-entry timestamp, and $t_{i}^{previous}$ is the previous-gantry timestamp. Accordingly, $\Delta t_{i}^{entry}$ represents the elapsed time from entry to the current transaction, whereas $\Delta t_{i}^{previous}$ represents the elapsed time from the previous gantry to the current transaction. When either timestamp failed the validity conditions, the corresponding duration was retained as missing rather than being assigned an artificial numerical value.

The data were partitioned before any context-dependent reference statistic was estimated. Gantries were stratified by their coarse G/S prefix and reproducibly assigned to training, validation, and test groups at nominal proportions of 70%, 15%, and 15%, using a fixed random seed of 20,221,213. The resulting groups contained 1768, 379, and 378 gantries, respectively. The observation day was divided into a training period from 00:00 to 17:59, a validation period from 18:00 to 20:59, and a test period from 21:00 to 23:59. The intersection of training gantries and the training period formed the core training subset (train_core), which contained 8,079,105 records. All contextual medians, median absolute deviations, category frequencies, and missingness-pattern frequencies were estimated exclusively from this subset and were frozen before application to any validation or test record.

The Cartesian combinations of the spatial and temporal partitions defined the analytical roles used in the experiments. The joint validation subset (validation_joint) comprised validation gantries during the validation period. The temporal-transfer test subset (test_temporal) comprised training gantries during the test period; the spatial-transfer test subset (test_spatial) comprised test gantries during the training period; and the joint-transfer test subset (test_joint) comprised test gantries during the test period. The remaining combinations were retained as auxiliary or secondary validation records. Table 4 reports the spatial group, temporal period, analytical purpose, and record count of each role.

Because the distributions of fee amounts and elapsed times were highly skewed, contextual deviations were quantified using a robust median-based distance. For a continuous attribute $z_{i}$ belonging to context $c_{i}$, the distance was calculated as:

(7) $$d_{i}^{MAD}\left(z\right)=\frac{\left|z_{i}-{\tilde{z}}_{c_{i}}\right|}{1.4826{MAD}_{c_{i}}\left(z\right)+1}$$

where $z_{i}$ is the observed value of the selected continuous attribute; $c_{i}$ denotes the context assigned to transaction i; ${\tilde{z}}_{c_{i}}$ is the median of the attribute among train_core records in context $c_{i}$; and ${MAD}_{c_{i}}\left(z\right)={median}_{l:c_{l}=c_{i}}\left|z_{l}-{\tilde{z}}_{c_{i}}\right|$ is the corresponding median absolute deviation (MAD). The factor 1.4826 gives a normal-consistent scaling of the median absolute deviation, whereas the additive constant 1 follows the implemented feature-construction procedure and prevents numerical instability when the empirical MAD is zero. All contextual statistics were estimated exclusively from train_core and were applied to the validation and test partitions without re-estimation.

Each labeling function mapped the available transaction attributes to one of three possible outputs:

(8) $$\lambda_{j}\left(x_{i}\right)\in \left\{-1,0,1\right\},\;\;j=1,2,\ldots .,J$$

where j indexes the labeling functions, J is the total number of candidate functions, −1 denotes abstention, 0 denotes normal evidence, and 1 denotes anomaly evidence. Abstention was used whenever a function’s required inputs were unavailable or its empirical context was insufficient.

Deterministic anomaly functions identified payable or net-total discrepancies, discount-total discrepancies, non-missing arrays that failed parsing, invalid entry timestamps, and conflicts between an available previous-gantry field and its timestamp. Contextual anomaly functions identified entry or previous-gantry duration deviations of at least 8 robust units, rare disagreements between original and billing vehicle types, rare out-of-dictionary code pairs, and uncommon missingness patterns containing additional suspicious evidence. Rare vehicle pairs and rare patterns were defined by at most 20 training observations. Conservative normal functions represented exact monetary consistency, plausible and non-extreme temporal relations, vehicle-type pairs observed at least 1000 times, and missingness patterns observed at least 10,000 times in train_core.

The candidate matrix initially contained ten anomaly-oriented and four normal-oriented functions. The monetary-array parsing function was retained as a deterministic integrity safeguard but produced no votes because every non-missing monetary array in the data was numerically parseable. Because this source had zero empirical coverage, it was excluded from probabilistic label-model fitting and consequently supplied no parser-specific weak supervision to the CAWS-ETC discriminator. Zero empirical coverage did not, however, invalidate the logical definition of the parsing check. The same predefined condition was therefore retained as a deterministic integrity safeguard for the rule-based positive-control comparators used in the controlled-intervention evaluation. Pairwise diagnostics further showed that the family-specific normal functions were highly overlapping and could conflict with legitimate anomaly evidence from another view. Because normality within one view did not establish global transaction normality, the four family-specific normal functions were combined into a conservative joint normal anchor:

(9) $$\lambda_{i}^{JN}=\{\begin{array}{c} 0,\lambda_{i,M}^{N}=\lambda_{i,T}^{N}=\lambda_{i,S}^{N}=\lambda_{i,C}^{N}=0\\ -1,\;otherwise \end{array}$$

where $\lambda_{i,M}^{N}$, $\lambda_{i,T}^{N}$, $\lambda_{i,S}^{N}$, and $\lambda_{i,C}^{N}$ denote the monetary, temporal, structural-pattern, and contextual–semantic normal functions for transaction i, respectively. The joint anchor $\lambda_{i}^{JN}$ emitted a normal vote only when all four normal conditions were simultaneously satisfied; otherwise, it abstained. The probabilistic label model consequently used nine active anomaly-oriented sources and one joint normal anchor. Table 5 summarizes the ten weak sources entered into the probabilistic label model. Full definitions and diagnostics for the complete candidate set are provided in the separate Supplementary Material Table S1.

### 3.3. Probabilistic Weak-Label Learning and Anomaly Scoring

The nine active anomaly-oriented sources and the joint normal anchor defined in Section 3.2 were integrated through a regularized binary latent-class model. Let $\lambda_{i}=\left(\lambda_{i1},\ldots ,\lambda_{iJ}\right)$ denote the selected weak-source outputs for transaction i. For labeling function j, define $\theta_{jlc}=P\left(\lambda_{ij}=l|y_{i}=c\right)$, where l∈{−1, 0, 1} is the observed weak vote and c∈{0, 1} is the latent class. Under the conditional-independence assumption, the observed-data log-likelihood is:

(10) $$L\left(\pi ,\Theta \right)=\sum_{i\in S_{LM}}log\left[\pi \prod_{j=1}^{J}\theta_{j,\lambda_{ij},1}+\left(1-\pi \right)\prod_{j=1}^{J}\theta_{j,\lambda_{ij},0}\right]$$

where $S_{LM}$ is the deterministic 500,000-record train_core sample used to fit the label model; $\pi =P\left(y_{i}=1\right)$ is the latent anomaly-class prevalence; and Θ is the collection of source-specific categorical probabilities. Expectation–maximization was used to estimate π and Θ. Orientation-preserving Dirichlet pseudo-counts were added during the parameter updates to avoid zero-probability estimates and latent-class switching.

Given the fitted prevalence and source-specific conditional probabilities, the posterior weak-label probability for transaction i was obtained as:

(11) $${\tilde{p}}_{i}=P\left(y_{i}=1|\lambda_{i}\right)=\frac{\pi \prod_{j=1}^{J}\theta_{j,\lambda_{ij},1}}{\pi \prod_{j=1}^{J}\theta_{j,\lambda_{ij},1}+\left(1-\pi \right)\prod_{j=1}^{J}\theta_{j,\lambda_{ij},0}}$$

where ${\tilde{p}}_{i}$ is the probabilistic weak-label score generated by the latent-class model. It represents the posterior probability under the fitted weak-source model and should not be interpreted as a manually verified probability of a real operational anomaly.

Three initializations were fitted, and the solution with the largest observed-data likelihood was retained after verifying that every anomaly-oriented function was more likely to vote under the latent anomaly state and that the joint normal anchor was more likely to vote under the latent normal state. All three fits converged after 58 iterations; the retained model yielded an estimated latent prevalence of 0.1852. This quantity was used only as a label-model parameter and was not interpreted as the true prevalence of anomalous ETC transactions.

The fitted model was applied to all 14,510,847 records. Posterior probabilities greater than or equal to 0.90 and less than or equal to 0.10 were designated as high-confidence anomaly and normal references, respectively. These original thresholds provided 682,628 anomaly candidates and 5,970,006 normal candidates in train_core; therefore, the pre-specified relaxed thresholds were not invoked. The remaining intermediate-probability records were excluded from discriminator training rather than being forced into either class.

The posterior probabilities were converted into high-confidence pseudo-labels for discriminator training:

(12) $${\hat{y}}_{i}=\{\begin{array}{c} 1,{\tilde{p}}_{i}\geq \tau_{A}\\ 0,{\tilde{p}}_{i}\leq \tau_{N}\\ \emptyset ,\tau_{N}<{\tilde{p}}_{i}<\tau_{A} \end{array},\tau_{A}=0.90,\tau_{N}=0.10$$

where ${\hat{y}}_{i}$ is the pseudo-label, $\tau_{A}$ and $\tau_{N}$ are the anomaly and normal confidence thresholds, and ∅ denotes exclusion from discriminator training. Records in the intermediate-probability region were retained for subsequent scoring but were not forced into either training class.

The high-confidence pseudo-labeled records were subsequently used to train a LightGBM discriminative model, hereafter referred to as the discriminator. A deterministic sample of 180,000 high-confidence anomaly records and 420,000 high-confidence normal records was selected from the core training subset. The joint validation subset supplied 25,251 high-confidence anomalies and 174,749 high-confidence normals for early stopping.

To account for both weak-label confidence and class imbalance, each selected training record received the following weight:

(13) $$c_{i}=min\left\{1,\;max\left[0.05,\;2\left|{\tilde{p}}_{i}-0.5\right|\right]\right\}$$

(14) $$w_{i}=c_{i}\frac{N_{sel}}{2N_{{\hat{y}}_{i}}}$$

where $c_{i}$ is the confidence component of the weight, $N_{sel}$ is the total number of selected high-confidence training records, and $N_{{\hat{y}}_{i}}$ is the number of selected records belonging to pseudo-class ${\hat{y}}_{i}$. The class factor assigns equal aggregate weight to the anomaly and normal classes. Because the selected records satisfied ${\tilde{p}}_{i}\geq 0.90$ or ${\tilde{p}}_{i}\leq 0.10$, their confidence components were close to one.

The discriminator was allowed a maximum of 2000 boosting iterations and used a learning rate of 0.03, 31 leaves, a minimum of 100 observations per leaf, and 80% feature subsampling. Validation-based early stopping selected iteration 157. Missing numerical values were retained as NaN and handled natively by the tree model, whereas categorical levels not observed during training were mapped to a dedicated __UNK__ level. Raw vehicle identifiers, original gantry identifiers, operational proxy fields, and labeling-function outputs were excluded from the model inputs. The fitted discriminator produced the following score for transaction i:

(15) $$s_{i}=f_{\theta }\left(x_{i}\right)$$

where $f_{\theta }$ denotes the fitted LightGBM discriminator with parameter set θ, and $s_{i}$ is the continuous CAWS-ETC anomaly-ranking score. Larger values indicate stronger learned inconsistency evidence. Because the training set was deliberately rebalanced and derived from weak labels, $s_{i}$ was not interpreted as a calibrated posterior probability of a real-world anomaly.

To support privacy-preserving operational screening, transaction scores were aggregated for pseudonymized gantry g and 15 min interval τ:

(16) $$B_{g,\tau }=\frac{1}{N_{g,\tau }}\sum_{i\in L_{g,\tau }}s_{i}$$

where $L_{g,\tau \;}$ is the set of transactions observed at gantry g during interval τ, and $N_{g,\tau }=|L_{g,\tau }|$ is the corresponding transaction count. $B_{g,\tau }$ is termed the gantry–time transaction anomaly burden. The primary visualization retained cells containing at least 10 transactions, while thresholds of 5 and 20 transactions were examined in the sensitivity analysis. All data processing, model fitting, evaluation, and visualization were implemented in Python 3.13. The label-model settings, pseudo-label selection criteria, LightGBM hyperparameters, training and validation sample sizes, and exclusion of operational proxy fields are summarized in Table 6.

### 3.4. Validation Strategy and Evaluation Protocol

The evaluation was organized into five complementary components. First, rule-aligned controlled interventions were used as positive controls to verify the recovery of mechanisms explicitly represented by the labeling functions. Second, temporal-, spatial-, and joint-transfer subsets were used to examine within-dataset transfer to later periods of the same observation day and to held-out gantries within the same provincial expressway network. These partitions were designed to reduce information leakage and assess internal transfer robustness; they do not constitute external validation across different dates, seasons, or regions. Third, blinded manual adjudication of unmodified transactions from the previously untouched joint-transfer subset was conducted as the principal real-record validation. Fourth, independent system-recorded outcomes were used as complementary association and high-score enrichment proxies without being treated as ground truth. Fifth, the rule-orthogonal challenge, retraining-based ablation, sensitivity analysis, tree-contribution analysis, and gantry–time aggregation were used to assess behavior beyond direct rule activation. The originally fitted CAWS-ETC and the four pre-specified comparator methods used in these analyses were frozen before manual sampling. A post hoc supervision-source attribution analysis was subsequently conducted to determine whether the ranking capability of the discriminative model specifically depended on probabilistic weak-label aggregation.

The rule-aligned challenge was implemented as a controlled feature-level intervention experiment rather than as a reconstruction of complete raw ETC transactions. Candidate base records were selected separately from validation_joint, test_temporal, test_spatial, and test_joint. Eligible records were required to have a weak-label probability of no more than 0.10 and to activate the joint normal anchor; mechanism-specific availability conditions were additionally imposed when a monetary sum or duration feature was required. Within each analytical role and intervention type, eligible record identifiers were deterministically ordered using a hash constructed from the record identifier, role, intervention type, and the fixed seed 20,221,213, after which the first 2000 records were retained. Thus, the same input data and seed reproduce the same base-record selection.

Nine rule-aligned mechanisms were applied at mild, moderate, and severe levels. For the payable/net-total mismatch, the imposed residual was max(1,|pay_fee|×f), where f was 0.01, 0.05, and 0.20, respectively; the payable and net residual features and their absolute values were set to this amount, and the corresponding component sums were reduced by the same amount. For the discount-total mismatch, the imposed residual was max(1,|pay_fee|×f), with f equal to 0.005, 0.02, and 0.10. Entry- and previous-duration outliers were assigned robust deviation values of 8.5, 12.0, and 20.0, together with increments of 0.50, 1.00, and 2.00 in the corresponding log-duration feature. The invalid-entry-time intervention set the entry-time validity indicator to false and the associated duration features to missing, whereas the previous-time-conflict intervention set previous-gantry presence and conflict indicators to true, previous-time validity to false, and the associated duration features to missing. These two validity interventions were binary; their field transformations were therefore identical across the three severity labels, which were retained to preserve the balanced factorial design.

For the semantic rare-pair intervention, the training pair count was set to 20, 5, and 0 for the three severity levels, and the vehicle-type disagreement indicator was activated; the moderate condition additionally used vehicle type code 255, whereas the severe condition used the dedicated __UNK__ category and activated the out-of-dictionary indicator. For the rare-missingness intervention, the corresponding pattern count was set to 20, 5, and 0, the payable- and net-fee component groups were marked unavailable, and their associated sums, residuals, and component-count features were set to missing. The parser-corruption stress condition activated the component-array parse-error indicator and set the group-count spread to 1, 2, and 3. The parser stress condition requires a distinction between empirical training coverage and deterministic rule availability. Although lf_a_group_parse had zero empirical coverage and was excluded from probabilistic label-model fitting, its predefined Boolean condition remained available as an explicit integrity safeguard. The hard-rule union and anomaly-vote fraction therefore retained this safeguard during controlled evaluation. Setting the parse-error indicator to true directly activated the safeguard in the intervention copy without requiring any parser-error examples during training. The same intervention also invalidated the monetary-normal condition and consequently removed the joint-normal anchor vote. Thus, although the parser anomaly source itself was absent from probabilistic fitting, the parser intervention changed the weak-evidence state by removing strong normal evidence. These rule and anchor effects were properties of the controlled evaluation design and should not be interpreted as learned generalization to an unseen parsing mechanism. Each selected base record generated one unchanged control and one intervention copy at each severity level. Consequently, the complete rule-aligned dataset contained 4 × 9 × 2000 × 3 × 2 = 432,000 records, corresponding to 216,000 matched control–intervention pairs. Pair identifiers were retained locally to verify that every pair contained exactly one control and one intervention. Exact feature-level transformations and eligibility requirements are provided in Supplementary Table S7. These constructed labels were known by design and were used as mechanism-recoverability controls rather than as estimates of the distribution of naturally occurring ETC anomalies.

Five methods constituted the pre-specified evaluation set: a hard-rule union, an unweighted anomaly-vote fraction, the probabilistic label model, an unsupervised Isolation Forest, and CAWS-ETC. Isolation Forest was fitted to 100,000 unlabeled train_core transactions using 200 trees and a maximum subsample of 10,000 observations per tree. None of the methods used transaction failure, fee-calculation status, or special-transaction indicators for fitting or threshold selection. For each method, a single decision threshold maximizing the F1-score was selected on the validation_joint controlled-intervention set. This threshold was then applied unchanged to the temporal-transfer, spatial-transfer, and joint-transfer test sets. Table 7 summarizes the methodological origin or representative introduction year, information sources, fitting configurations, outputs, threshold use, and interpretive roles of all seven methods. The first five constitute the pre-specified evaluation set, whereas the final two are post hoc attribution models introduced only for supervision-source analysis.

The controlled-intervention analysis reported the AUROC, AUPRC, precision, recall, F1-score, specificity, false-positive rate (FPR), and true-positive rate (TPR) at 1% FPR. Results were further stratified by intervention type and severity. Confidence intervals for the principal overall metrics were estimated using 100 bootstrap resamples. Low-prevalence stress tests were constructed from the test_joint rule-aligned intervention pool using a fixed total of 100,000 records per mixture. Target intervention prevalences of 1%, 3%, and 5% therefore corresponded to 1000, 3000, and 5000 intervention records and 99,000, 97,000, and 95,000 control records, respectively. Intervention and control records were sampled separately using deterministic random states derived from the fixed seed 20,221,213; sampling with replacement was permitted only when the requested number exceeded the available pool. The decision threshold remained the one selected on validation_joint and was not re-optimized for any target prevalence. These mixtures were designed solely to examine metric sensitivity to class imbalance and were not interpreted as estimates of the true operational anomaly prevalence.

To provide an evaluation independent of both controlled interventions and system-recorded operational proxies, a blinded manual audit was conducted on unmodified transactions from the previously untouched test_joint subset. The previously fitted CAWS-ETC model, the four pre-specified comparator methods, feature definitions, and all decision thresholds used in the blinded manual-validation experiment were frozen before manual sampling. The 158,896 joint-transfer transactions were divided into four tie-preserving CAWS-ETC score strata corresponding approximately to the highest 1%, 1–5%, 5–20%, and the remaining 80% of the score distribution. Three hundred transactions were sampled from each stratum using a fixed random seed, yielding 1200 records for independent review. Of these records, 1189 could be uniquely linked to the required raw transaction details, whereas 11 did not have a unique raw-detail match. Records lacking sufficient evidence were not forced into a binary classification.

Two reviewers independently examined the anonymized transactions using a common adjudication protocol. Both reviewers were blinded to the CAWS-ETC score, score stratum, probabilistic weak label, individual labeling-function outputs, comparator scores, model-derived validity flags, and the three operational proxy fields. Each transaction was classified as NORMAL, ANOMALY, or INDETERMINATE. An ANOMALY label required a directly verifiable inconsistency in the displayed transaction information, whereas an INDETERMINATE label was used when the available fields were insufficient for a defensible binary judgment. Initial inter-reviewer reliability was quantified using exact agreement and three-category Cohen’s κ. Disagreements were resolved by consensus between the two reviewers, while the original independent judgments were retained for reliability analysis.

Records with a final INDETERMINATE label were excluded from binary detection metrics. Because equal numbers of records were sampled from score strata with substantially different population sizes, inverse sampling-probability weights were used to recover population-representative performance estimates among determinate adjudications. For score stratum h, the sampling weight was defined as:

(17) $$w_{h}=\frac{N_{h}}{n_{h}}$$

where $N_{h}$ denotes the number of joint-transfer transactions in stratum *h* and $n_{h}$ denotes the number manually reviewed from that stratum. Sampling-weighted AUPRC was specified as the primary manual-validation metric. Weighted AUROC, precision, recall, F1-score, specificity, and false-positive rate were reported as complementary metrics. Importantly, all classification metrics used the thresholds previously selected on the validation_joint controlled-intervention set; the manually adjudicated labels were not used for model fitting, threshold selection, or score recalibration. Confidence intervals were estimated using 1000 stratified bootstrap resamples.

Independent validation used three system-recorded outcomes that had been excluded from all feature-reference estimation, weak-label construction, model fitting, and calibration: transaction failure (trade_result = 1), the presence of a special-transaction code, and a nonzero fee-calculation status. AUROC and AUPRC were calculated for their association with each method score in the three test roles. High-score enrichment was evaluated using score cutoffs derived from the test-score distributions. Because several methods produced tied scores, final enrichment estimates used inclusive tie-aware cutoffs rather than arbitrarily splitting identical score groups. These analyses quantify association and prioritization value; the operational fields were not interpreted as manually verified anomaly ground truth.

Let $Z_{i}^{\left(m\right)}\in \left\{0,1\right\}$ denote whether transaction i exhibits operational proxy m, where m represents transaction failure, the presence of a special-transaction code, or nonzero fee-calculation status. For a requested upper-score fraction q, the inclusive score set was defined as $T_{q}=\left\{i:s_{i}\geq c_{q}\right\}$, where $c_{q}$ is the empirical lower quantile at probability 1−q. All records tied at $c_{q}$ were retained. Proxy enrichment was calculated as:

(18) $${Lift}_{q,m}=\frac{\frac{1}{\left|T_{q}\right|}\sum_{i\in T_{q}}Z_{i}^{\left(m\right)}}{\frac{1}{N}\sum_{i=1}^{N}Z_{i}^{\left(m\right)}}$$

where the numerator is the proxy-event rate in the inclusive high-score set and the denominator is the proxy-event rate in the complete evaluation set. Because tied scores could cause the achieved fraction $|T_{q}|/N$ to differ from the requested fraction q, both fractions were reported.

Following inspection of the rule-aligned diagnostics, a separate rule-orthogonal feature-level challenge was constructed to test ranking beyond direct labeling-function activation. Candidate bases were again drawn from the four held-out roles using a weak-label probability of no more than 0.10 and an active joint normal anchor. For each role and each of the six challenge mechanisms, 1500 eligible base records were selected deterministically by hash ordering with seed 20,221,213. Before a control–intervention pair was created, both copies were transformed to a common non-anchor ambiguous reference state: pair_train_count was set to 999, its rarity score was recalculated as log[(Ntrain + 1)/1000], and the vehicle-type disagreement indicator was set to false. This normalization placed the semantic evidence immediately below the normal-anchor frequency requirement without crossing the anomaly threshold.

Three subthreshold severity levels were then defined using robust deviation values of 4.5, 6.0, and 7.5, all below the anomaly-labeling threshold of 8.0. The contextual-fee intervention assigned these values to both payable- and net-fee contextual deviations and increased their signed log-fee features by 0.10, 0.30, and 0.60. The entry- and previous-duration interventions assigned the same robust deviation values to the corresponding duration feature and increased its log-duration value by 0.20, 0.50, and 0.80. The semantic mid-frequency intervention activated vehicle-type disagreement while setting the pair count to 999, 200, and 21. The missingness-pattern intervention set the pattern count to 9999, 500, and 21 and introduced payable-group unavailability while keeping the pattern above the anomaly-rule threshold. Finally, the multiview intervention simultaneously combined the subthreshold payable-context and entry-duration deviations with pair counts of 500, 100, and 21 and pattern counts of 5000, 200, and 21.

After construction, all selected labeling functions were recomputed for every control and intervention record. The challenge was accepted only when no primary anomaly-oriented source fired for any record and when every record remained outside the joint normal anchor. The two members of each pair therefore shared the same all-abstaining/non-anchor weak-source state, while differing in the underlying continuous or contextual feature values. The complete challenge contained 4 × 6 × 1500 × 3 × 2 = 216,000 records, or 108,000 matched control–intervention pairs. The exact field modifications and mechanism-specific availability conditions are listed in Supplementary Table S7.

The manual adjudications were not used to construct labels, select training records, fit either attribution model, select model capacity, or tune any decision threshold. They were reused only as an independent evaluation set after the attribution models had been fitted.

A direct-rule LightGBM comparator was trained from binary labels constructed directly from the selected rule states. A transaction was assigned an anomaly training label when at least one selected anomaly-oriented source was active and the joint normal anchor was inactive. A normal training label was assigned when the joint normal anchor was active and no anomaly-oriented source was active. Records satisfying neither condition were excluded from discriminator training rather than being forced into either class.

For controlled component attribution, one deterministic shared sample of 600,000 core-training records, comprising 180,000 anomaly and 420,000 normal records, was used identically by the direct-rule LightGBM and a matched confidence-weighted LightGBM reference. Both models used the same 46 input features, LightGBM hyperparameters, random seed, and 157 boosting iterations, corresponding to the model capacity selected for the originally fitted CAWS-ETC. The direct-rule LightGBM used class-balanced weights only, whereas the matched confidence-weighted model additionally retained the posterior-confidence component defined in Equation (13). The originally fitted CAWS-ETC was not retrained and retained its original training-sample realization.

No new classification threshold was selected for this attribution analysis. AUROC and AUPRC were used as threshold-free ranking metrics on the rule-orthogonal transfer sets and on the 777 determinate manual labels. Paired AUPRC differences were estimated from 1000 bootstrap replicates, clustered by control–intervention pair for the joint rule-orthogonal challenge and stratified by the original manual-audit score strata for the sampling-weighted manual evaluation.

Robustness was further assessed through retraining-based feature-family ablation. Separate LightGBM models were fitted after removing the monetary, temporal, structural, or contextual–semantic feature family. Additional variants examined source-balanced anomaly sampling, training samples of 100,000, 300,000, and 600,000 records, weak-label confidence thresholds of 0.85/0.15, 0.90/0.10, and 0.95/0.05, and three combined sampling/model-seed configurations. Because the latter variants changed both sampled records and model random states, they were interpreted as combined stochastic sensitivity rather than pure model-seed sensitivity. Model interpretation used the tree-contribution measure in Equation (19). Finally, gantry–time burden distributions were compared after requiring at least 5, 10, or 20 transactions per aggregation cell.

Model interpretation was based on LightGBM tree-contribution values calculated for a deterministic sample of 50,000 test_joint records. Let $\phi_{ik}$ denote the signed contribution of feature k to the score of transaction i. Excluding the expected-value term, the normalized global contribution of feature k was calculated as:

(19) $$C_{k}=\frac{\sum_{i\in S_{\phi }}\left|\phi_{ik}\right|}{\sum_{h=1}^{P}\sum_{i\in S_{\phi }}\left|\phi_{ih}\right|}$$

where $S_{\phi }$ is the 50,000-record interpretation sample, P=46 is the number of model-input features, and $C_{k}$ is the share of the total mean absolute tree contribution attributable to feature k. Signed contributions were additionally summarized within feature-value deciles to examine the direction of their effects.

## 4. Results

### 4.1. Dataset Profile and Weak-Supervision Diagnostics

The quality control workflow retained 14,510,847 ETC gantry transactions recorded at 2525 gantries over the complete 24 h period of 13 December 2022. No duplicated transaction identifiers, conflicting identifier groups, or fully duplicated records were detected. The refined audit identified 78,409 payable-total mismatches, 8620 discount-total mismatches, and 78,203 net-total mismatches. No mismatch occurred among the lengths of the toll-interval and monetary component arrays. Temporal validity checks identified 197,650 entry timestamps and 2,599,253 previous-gantry timestamps preceding the year-2000 plausibility boundary. In addition, 475 entry timestamps and 986 previous-gantry timestamps occurred after the corresponding transaction time. These records were retained because the inconsistencies themselves supplied potentially useful sensing evidence.

The spatial and temporal partitioning preserved all transactions. The 2525 gantries were divided into 1768 training, 379 validation, and 378 test gantries. Their intersection with the training period produced 8,079,105 train_core records. All contextual medians, median absolute deviations, category frequencies, and missingness frequencies were estimated from this subset only. The final analytical representation consisted of 46 model-input features. The system-recorded transaction outcome, fee-calculation status, and special-transaction fields were retained exclusively for independent evaluation. The principal data-audit results and the record counts of the spatial–temporal partitions are reported in Table 8.

The candidate weak-source matrix initially contained ten anomaly-oriented and four normal-oriented labeling functions. The monetary-array parsing function generated no votes because all non-missing arrays were numerically parseable. The four broad normal functions were highly overlapping and could conflict with valid anomaly evidence from another information view. They were therefore replaced by one joint normal anchor, which emitted a normal vote only when the monetary, temporal, semantic, and missingness conditions were simultaneously satisfied. The final probabilistic model used nine active anomaly sources and one joint normal anchor.

Across the complete dataset, 10,639,650 transactions (73.322%) activated the joint normal anchor, 1,250,971 (8.621%) received at least one anomaly-oriented vote, and 2,620,226 (18.057%) remained outside both conditions and were therefore weakly ambiguous. The latent label model converged under all three initializations after 58 iterations. The retained solution estimated a latent anomaly-class prevalence of 0.1852. This parameter characterizes the fitted latent mixture and is not interpreted as the observed or true anomaly rate.

Application of the fitted label model yielded 682,628 high-confidence anomaly candidates and 5,970,006 high-confidence normal candidates within train_core at the 0.90/0.10 probability thresholds. A LightGBM discriminator was trained on 180,000 anomaly and 420,000 normal records and selected 157 boosting iterations through validation-based early stopping. The internal AUROC and AUPRC against high-confidence weak labels were approximately 1.0. These values indicate successful transfer of the weak evidence into the discriminator, rather than independently verified anomaly accuracy. Full-data inference covered all 14,510,847 transactions in 87.3 s and produced 233,476 pseudonymized gantry–15 min aggregation cells. Table 9 summarizes the fitted label-model diagnostics, high-confidence sample counts, LightGBM training configuration, internal weak-label agreement, and full-data inference efficiency.

**Figure 2:**
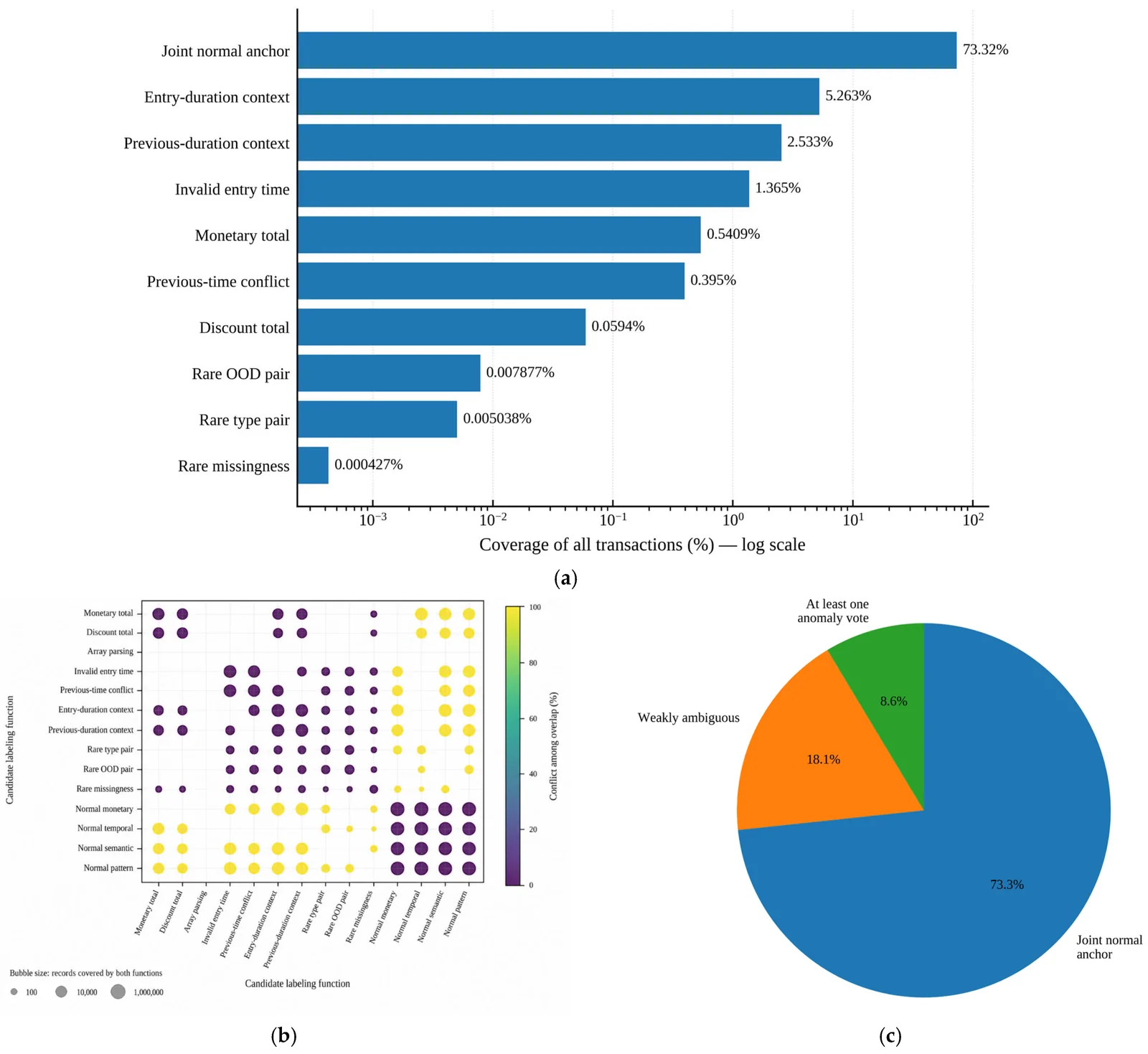
Weak-supervision diagnostics for the complete ETC dataset: (**a**) coverage of the active weak sources; (**b**) pairwise overlap and conflict among weak sources; and (**c**) distribution of joint-normal, anomaly-vote, and ambiguous weak-evidence states.

Figure 2 presents the empirical weak-supervision diagnostics and can be read as a summary of how frequently and jointly the programmatic evidence sources operate. In panel (a), coverage denotes the proportion of all transactions for which a weak source emits evidence; the logarithmic horizontal scale is used because source activation frequencies differ by several orders of magnitude. A larger bar therefore indicates more frequent activation, not necessarily a more accurate source. Panel (b) shows how pairs of weak sources interact: overlap indicates that two sources are activated on some of the same transactions, whereas conflict indicates that they provide opposing evidence on shared records. Panel (c) summarizes the resulting evidence states at the transaction level: 73.322% of records received the joint-normal anchor, 8.621% received at least one anomaly vote, and 18.057% remained weakly ambiguous. Thus, the figure shows that anomaly-oriented evidence is comparatively sparse and heterogeneous, while a substantial subset cannot be reduced to an unambiguous rule-based decision.

### 4.2. Anomaly Detection Performance and Within-Dataset Transferability

The first validation experiment used rule-aligned consistency interventions as positive controls. The intervention set contained 432,000 records formed from 216,000 matched control–intervention pairs across temporal, spatial, and joint transfer conditions. The hard-rule union and anomaly-vote fraction achieved perfect aggregate recovery because their controlled-evaluation definitions included the deterministic parser safeguard in addition to the nine primary anomaly-oriented sources; each rule-aligned intervention therefore directly activated at least one evaluated deterministic condition. The probabilistic label model also achieved perfect aggregate recovery, but the parser mechanism followed a different pathway: the zero-coverage parser source was absent from label-model fitting, whereas the injected parse-error state removed the joint-normal anchor vote and shifted the affected records away from the strongly normal weak-evidence state. These results verify the consistency of the intervention and scoring implementations. They should not be interpreted as independent evidence that the fitted models learned all corresponding real-world anomaly mechanisms.

**Figure 3:**
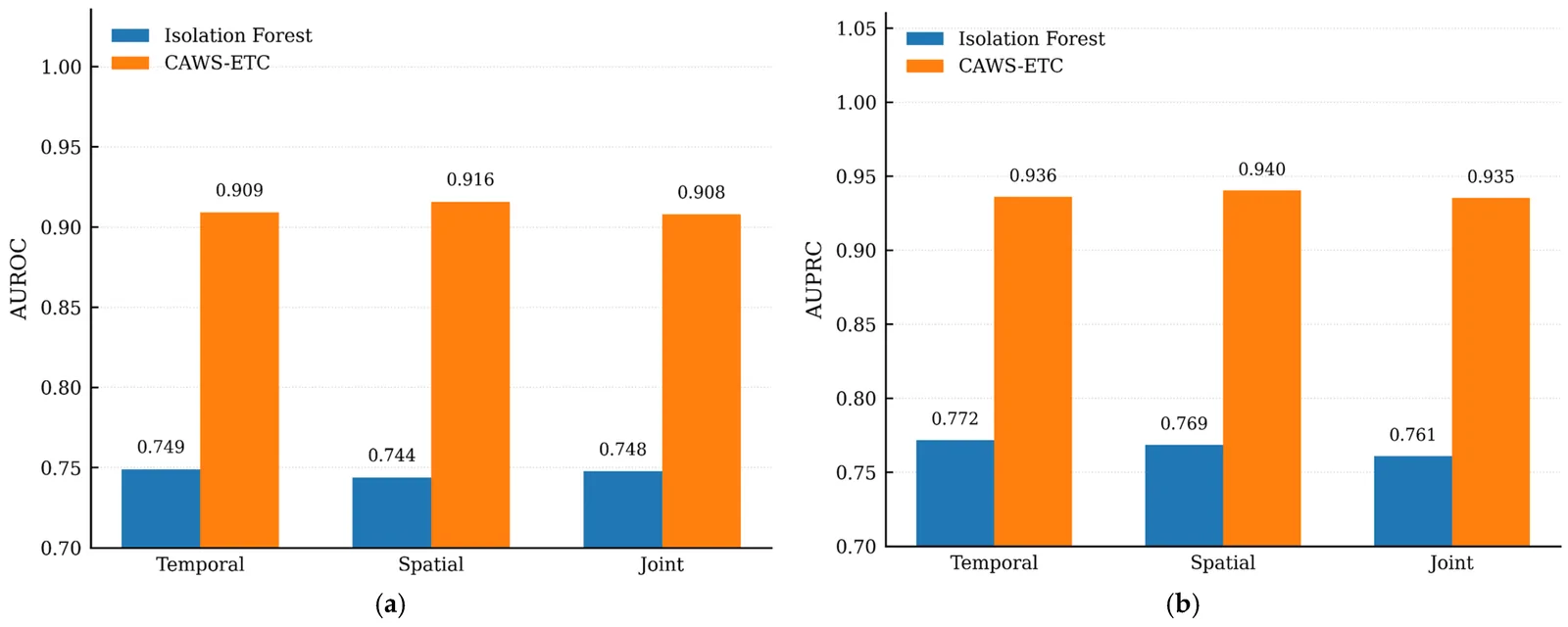
Transfer performance of CAWS-ETC and Isolation Forest under temporal, spatial, and joint transfer: (**a**) AUROC; and (**b**) AUPRC.

CAWS-ETC produced comparable ranking and classification results across the three held-out scenarios. Its AUROC values were 0.9090, 0.9158, and 0.9079 for temporal, spatial, and joint transfer, respectively, while the corresponding AUPRC values were 0.9361, 0.9404, and 0.9354. The F1-score ranged from 0.8601 to 0.8776. In contrast, Isolation Forest yielded AUPRC values between 0.7609 and 0.7718 and exhibited substantially higher false-positive rates. The limited variation among the three CAWS-ETC results indicates robustness to these within-day and within-network holdouts. Because all three test subsets were drawn from the same observation day and the same provincial expressway system, however, these results should not be interpreted as evidence of cross-day, seasonal, or cross-region transferability. Table 10 reports the complete method-level results for the temporal-, spatial-, and joint-transfer subsets, with the deterministic methods retained as rule-recovery positive controls rather than as independent data-driven competitors. Figure 3 visualizes the AUROC and AUPRC of CAWS-ETC and Isolation Forest across the three transfer settings.

For readers less familiar with anomaly-detection metrics, AUROC summarizes how well a score separates intervention and control records across possible thresholds, whereas AUPRC emphasizes the balance between retrieving anomalous records and limiting false alerts. For both metrics, larger values indicate stronger ranking performance. In Figure 3, each pair of bars compares CAWS-ETC with Isolation Forest under the same holdout condition. “Temporal” refers to later periods of the same observation day, “spatial” to held-out gantries within the same expressway network, and “joint” to their combination. The similar CAWS-ETC bars across the three settings therefore indicate stable within-dataset ranking under these holdouts, while their consistently higher values than Isolation Forest show the performance difference on this controlled benchmark. These comparisons should not be interpreted as evidence of cross-day or cross-region generalizability.

Performance nevertheless varied across anomaly mechanisms. Monetary-total mismatch, invalid entry time, entry-duration outlier, previous-duration outlier, rare semantic pairing, and rare missingness interventions were almost completely recovered. Previous-time conflicts remained strongly detectable in the joint-transfer set, with an AUPRC of 0.9469 and an F1-score of 0.9501. Discount-total mismatch was more difficult, yielding an AUPRC of 0.6695 and a recall of 0.3107. The parsing-corruption stress condition exposed the distinction between direct rule execution and learned transfer. The hard-rule union and anomaly-vote fraction perfectly separated the parser-control pairs because the predefined parser safeguard was explicitly retained in their controlled-evaluation definitions and was directly activated by the injected parse-error indicator. This result required no parser-error examples during training. The probabilistic label model also separated the pairs, but for a different reason: lf_a_group_parse itself had been excluded because of zero empirical coverage, whereas the parse-error intervention suppressed the joint-normal anchor and thereby shifted the intervention records away from the strongly normal weak-evidence state. In contrast, CAWS-ETC remained at chance-level ranking for this mechanism (AUROC = 0.5000; AUPRC = 0.5000; recall = 0.0340 at the frozen threshold), consistent with the absence of parser-specific training support. Isolation Forest likewise provided chance-level threshold-free ranking. Thus, perfect performance of the deterministic safeguards reflects direct execution of a predefined integrity condition rather than statistical generalization from observed parser errors.

The low-prevalence stress tests demonstrated the practical effect of class imbalance. At synthetic anomaly proportions of 1%, 3%, and 5%, CAWS-ETC achieved AUPRC values of 0.6945, 0.7565, and 0.7860, respectively. Recall remained between 0.7880 and 0.8148, whereas precision increased from 0.1864 at 1% prevalence to 0.5523 at 5%. Thus, high ranking performance does not eliminate false alerts when the operational anomaly rate is very low.

**Figure 4:**
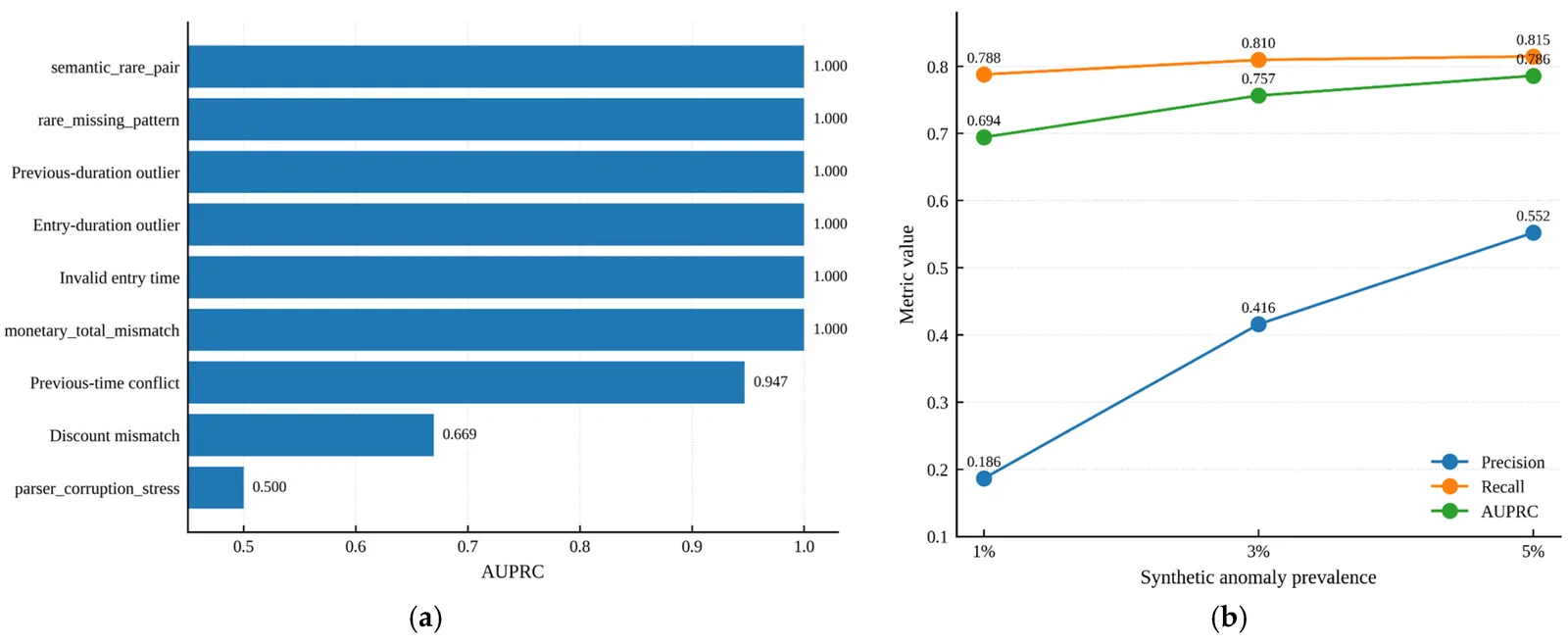
Mechanism-specific and prevalence-sensitive performance of CAWS-ETC: (**a**) joint-transfer AUPRC by intervention type; and (**b**) precision, recall, and AUPRC at synthetic anomaly proportions of 1%, 3%, and 5%.

Figure 4 compares CAWS-ETC performance across the nine rule-aligned intervention mechanisms and reports the change in precision, recall, and AUPRC when the synthetic anomaly proportion decreases from 5% to 1%. Extended role-, mechanism-, and severity-specific results are provided in the separate Supplementary Material Table S2. The complete field-level intervention definitions, severity values, deterministic sampling rules, and pair-construction checks used to generate the rule-aligned and rule-orthogonal datasets are provided in Supplementary Table S7.

Figure 4 provides two complementary views of model behavior. Panel (a) shows that performance is mechanism-dependent rather than uniform. Most rule-aligned mechanisms were ranked with AUPRC values close to 1, whereas discount-total mismatch was more difficult (AUPRC = 0.6695), and parser corruption remained at chance-level ranking (AUPRC = 0.5000). Panel (b) illustrates a different operational issue. When the synthetic anomaly proportion decreased from 5% to 1%, recall remained comparatively stable (approximately 0.79–0.81), but precision decreased from 0.5523 to 0.1864 and AUPRC decreased from 0.7860 to 0.6945. In practical terms, the same ranking model can therefore generate a substantially larger proportion of false alerts when true anomalies are rare, even if its ability to retrieve anomalous records changes relatively little. This is why CAWS-ETC is intended primarily for ranked review prioritization rather than automatic transaction adjudication.

The blinded manual audit provided a direct evaluation of unmodified real transactions. The two reviewers initially agreed on 1032 of the 1200 reviewed records, corresponding to 86.0% exact agreement and a three-category Cohen’s κ of 0.786. The remaining 168 records were resolved by consensus between the two reviewers. Final adjudication yielded 546 anomalous, 231 normal, and 423 indeterminate transactions. Consequently, 777 records with determinate NORMAL or ANOMALY labels were retained for binary performance evaluation. All 546 manually confirmed anomalies were categorized as temporal inconsistencies, whereas all 423 indeterminate records were retained as a separate outcome rather than being forced into either binary class.

Against the determinate manual labels, CAWS-ETC achieved a sampling-weighted AUROC of 0.7901 (95% confidence interval [CI]: 0.7061–0.8868) and an AUPRC of 0.7946 (95% CI: 0.7442–0.8599). At the decision threshold fixed before manual review, precision, recall, F1-score, and specificity were 0.6126, 0.8016, 0.6944, and 0.8036, respectively. Isolation Forest produced higher point estimates for manual-label ranking, with an AUROC of 0.9687 and AUPRC of 0.8665, but its frozen-threshold precision and specificity were 0.4105 and 0.4438, while recall reached 1.0000. Thus, the real-record audit did not support a universal ranking advantage of CAWS-ETC over the unsupervised comparator. Instead, the two models exhibited different operating characteristics on the manually confirmed temporal anomalies.

**Figure 5:**
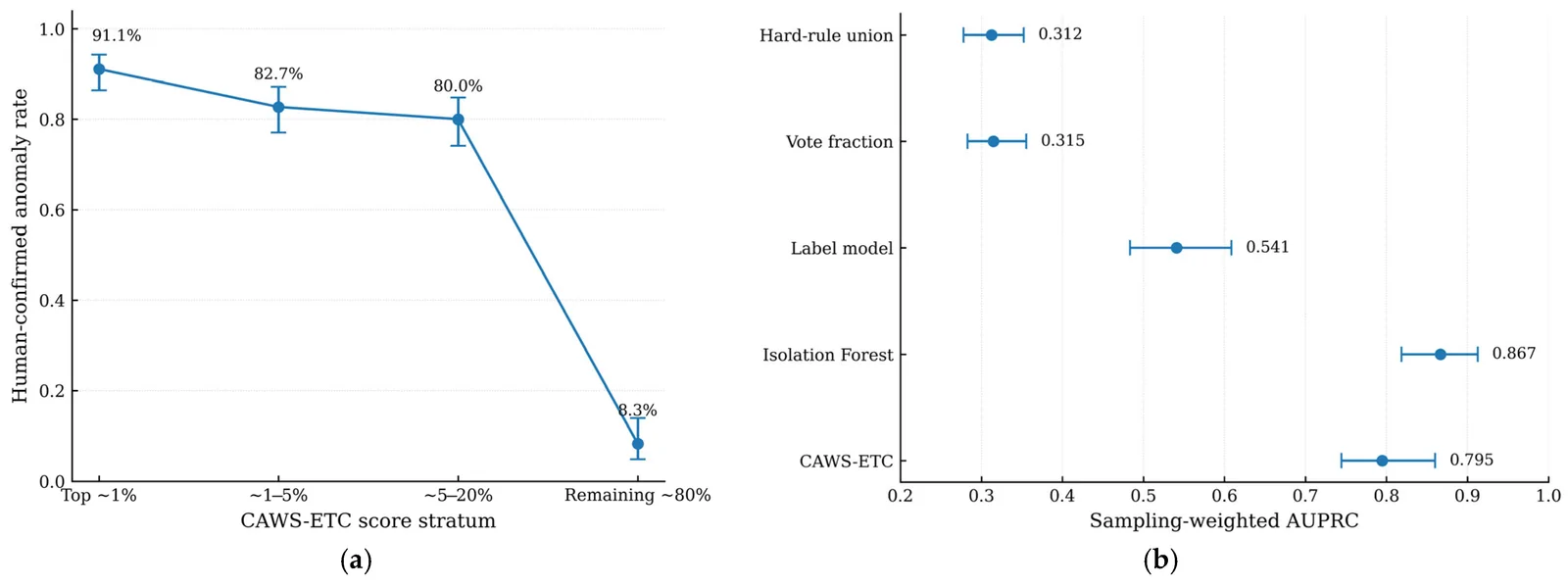
Independent blinded manual validation on unmodified joint-transfer transactions: (**a**) human-confirmed anomaly rate among determinate adjudications across tie-aware CAWS-ETC score strata, with 95% confidence intervals; and (**b**) sampling-weighted AUPRC of the five pre-specified methods evaluated in the blinded manual-validation experiment.

The score-stratified analysis nevertheless demonstrated strong prioritization by CAWS-ETC. Among records receiving determinate manual labels, the human-confirmed anomaly rate was 91.1% in the highest-score stratum, 82.7% in the approximately 1–5% stratum, 80.0% in the approximately 5–20% stratum, and 8.3% in the remaining approximately 80% of the score distribution. This marked separation indicates that unmodified real transactions with manually verifiable inconsistencies were strongly concentrated toward the upper end of the CAWS-ETC ranking. Table 11 summarizes the manual-validation results, and Figure 5 presents the score-stratum gradient and method-level AUPRC comparison.

Independent proxy evaluation provided a complementary source of evidence. No proxy field was used for feature-reference estimation, weak-label generation, model fitting, early stopping, or threshold selection. For transaction failure, CAWS-ETC achieved AUROC values of 0.9850, 0.9810, and 0.9796 across temporal, spatial, and joint testing. The corresponding values for nonzero fee-calculation status were 0.9875, 0.9823, and 0.9830. Association with the broader special-transaction indicator was weaker but remained substantial, with AUROC values ranging from 0.7869 to 0.8477. These results indicate that the consistency score concentrated records with operational exceptions, but they do not convert the proxies into manually verified anomaly labels.

Tie-aware enrichment analysis confirmed the prioritization value of the score. In the joint-transfer set, the inclusive top-score group nearest the requested 1% contained 1.109% of records and increased the transaction-failure, special-status, and nonzero fee-calculation rates by factors of 5.78, 4.14, and 5.55, respectively. The inclusive group nearest the requested 5% contained 5.127% of records and produced corresponding lifts of 5.28, 3.77, and 5.11. The achieved fraction is reported because identical high scores were never split arbitrarily. Detailed operational-proxy association and tie-aware enrichment results are provided in Supplementary Table S6 and Supplementary Figure S1.

Table 11 and Figure 5 report the five pre-specified methods that were fixed before manual sampling. The later post hoc supervision-source attribution models were not part of the manual-audit sampling design and are reported separately in Section 4.3 using the same determinate labels solely for threshold-free evaluation; the manual labels were not used for fitting or tuning those models.

### 4.3. Supervision-Source Attribution, Robustness, Interpretability, and Computational Performance

A rule-orthogonal challenge set was constructed to test whether the discriminator learned continuous or multiview structures beyond direct rule activation. The dataset contained 216,000 records covering six intervention mechanisms. Neither the normalized control records nor their paired intervention records triggered a primary anomaly labeling function, and both members of every pair remained in the same non-anchor ambiguous weak-evidence state. Under this stricter design, the hard-rule union and vote fraction remained at chance level. The probabilistic label model was also non-discriminative because the weak-source pattern was intentionally held constant.

CAWS-ETC retained AUPRC values of 0.9023, 0.9014, and 0.9018 under temporal, spatial, and joint transfer, compared with 0.7462, 0.7574, and 0.7377 for Isolation Forest. These pre-specified results demonstrated ranking capability beyond direct weak-source activation, but did not by themselves identify whether that capability arose from probabilistic aggregation or from the downstream discriminative learner. Performance was strongest for subthreshold entry-duration, mid-frequency missingness, and multiview interventions, each of which produced an AUPRC of 1.0 in the joint test. Semantic mid-frequency and previous-duration interventions yielded AUPRC values of 0.8865 and 0.8574. Contextual fee shifts remained undetected, with an AUPRC of 0.4951, indicating that the current model does not generalize to every rule-orthogonal mechanism.

The severity analysis produced the gradient that was absent from the rule-aligned experiment. In the joint-transfer condition, mild interventions yielded an AUPRC of 0.8046, whereas moderate and severe interventions yielded 0.9460 and 0.9455. The near equality of the two upper levels suggests score saturation after a sufficiently large multivariate deviation, but the clear separation from the mild condition confirms sensitivity below the hard-rule thresholds.

The threshold-based metrics in Table 12 require separate interpretation. Because each rule-orthogonal challenge stratum contains equal numbers of control and intervention records, the combination of precision = 0.5000 and recall = 1.0000 means that the frozen CAWS-ETC decision threshold classified all records in these strata as positive. This threshold had been selected on the rule-aligned validation_joint set and was deliberately applied without recalibration to the rule-orthogonal challenge. Therefore, these values indicate that the previously selected classification threshold does not transfer as a useful binary decision boundary to this shifted challenge distribution, even though the threshold-free AUROC and AUPRC values show that the continuous scores can still rank controls and interventions effectively.

**Table 12:** Rule-orthogonal challenge performance by method, mechanism, and severity. (A) Rule-orthogonal method-level ranking. (B) CAWS-ETC performance by challenge mechanism in the joint-transfer subset. (C) CAWS-ETC performance by severity in the joint-transfer subset.

**(A)**						
**Test Subset**		**Method**		**Records**	**AUROC**	**AUPRC**
Temporal-transfer test		Hard-rule union		54,000	0.5000	0.5000
Temporal-transfer test		Anomaly-vote fraction		54,000	0.5000	0.5000
Temporal-transfer test		Probabilistic label model		54,000	0.5000	0.5000
Temporal-transfer test		Isolation Forest		54,000	0.7561	0.7462
Temporal-transfer test		CAWS-ETC		54,000	0.8454	0.9023
Spatial-transfer test		Hard-rule union		54,000	0.5000	0.5000
Spatial-transfer test		Anomaly-vote fraction		54,000	0.5000	0.5000
Spatial-transfer test		Probabilistic label model		54,000	0.5000	0.5000
Spatial-transfer test		Isolation Forest		54,000	0.7622	0.7574
Spatial-transfer test		CAWS-ETC		54,000	0.8422	0.9014
Joint-transfer test		Hard-rule union		54,000	0.5000	0.5000
Joint-transfer test		Anomaly-vote fraction		54,000	0.5000	0.5000
Joint-transfer test		Probabilistic label model		54,000	0.5000	0.5000
Joint-transfer test		Isolation Forest		54,000	0.7595	0.7377
Joint-transfer test		CAWS-ETC		54,000	0.8444	0.9018
**(** **B** **)**						
**Challenge Mechanism**	**Records**	**AUROC**	**AUPRC**	**Precision**	**Recall**	**F1**
contextual_fee_shift	9000	0.4936	0.4951	0.5000	1.0000	0.6667
entry_duration_subthreshold	9000	1.0000	1.0000	0.5000	1.0000	0.6667
multiview_subthreshold	9000	1.0000	1.0000	0.5000	1.0000	0.6667
pattern_midfrequency_shift	9000	1.0000	1.0000	0.5000	1.0000	0.6667
previous_duration_subthreshold	9000	0.7406	0.8574	0.5000	1.0000	0.6667
semantic_midfrequency_shift	9000	0.8333	0.8865	0.5000	1.0000	0.6667
**(C)**						
**Severity**	**Records**	**AUROC**	**AUPRC**	**Precision**	**Recall**	**F1**
Mild	18,000	0.7019	0.8046	0.5000	1.0000	0.6667
Moderate	18,000	0.9161	0.9460	0.5000	1.0000	0.6667
Severe	18,000	0.9152	0.9455	0.5000	1.0000	0.6667

Note: Panel (A) reports the five pre-specified evaluation methods. The post hoc supervision-source attribution models are reported separately in Table 13.

**Table 13:** Post hoc supervision-source attribution using direct-rule and matched confidence-weighted LightGBM models.

**(A)**				
**Evaluation**	**Method**	**Records**	**AUROC**	**AUPRC**
Temporal rule-orthogonal	Direct-rule LightGBM	54,000	0.8889	0.9270
	Matched confidence-weighted LightGBM	54,000	0.8889	0.9270
	Originally fitted CAWS-ETC	54,000	0.8454	0.9023
Spatial rule-orthogonal	Direct-rule LightGBM	54,000	0.8898	0.9276
	Matched confidence-weighted LightGBM	54,000	0.8898	0.9276
	Originally fitted CAWS-ETC	54,000	0.8422	0.9014
Joint rule-orthogonal	Direct-rule LightGBM	54,000	0.8906	0.9280
	Matched confidence-weighted LightGBM	54,000	0.8906	0.9280
	Originally fitted CAWS-ETC	54,000	0.8444	0.9018
Manual determinate	Direct-rule LightGBM	777	0.7904	0.8033
	Matched confidence-weighted LightGBM	777	0.7904	0.8033
	Originally fitted CAWS-ETC	777	0.7901	0.7946
**(B)**				
**Evaluation**	**Paired Comparison**	**∆AUPRC**	**95% CI**	
Joint rule-orthogonal	Matched confidence-weighted—Direct-rule	+0.000002	−0.000007 to +0.000011	
Joint rule-orthogonal	Original CAWS-ETC—Direct-rule	−0.026235	−0.027563 to −0.024843	
Manual determinate	Matched confidence-weighted—Direct-rule	0.000000	0.000000 to 0.000000	
Manual determinate	Original CAWS-ETC—Direct-rule	−0.008720	−0.014305 to −0.002409	

Note: The direct-rule and matched confidence-weighted LightGBM models used identical training record IDs, binary classes, model-input features, LightGBM hyperparameters, random seed, and 157 boosting iterations. Manual metrics were corrected using inverse sampling-probability weights. The original CAWS-ETC retained its originally fitted training-sample realization; comparisons involving the original model are therefore interpreted as system-level rather than fully matched component comparisons.

**Figure 6:**
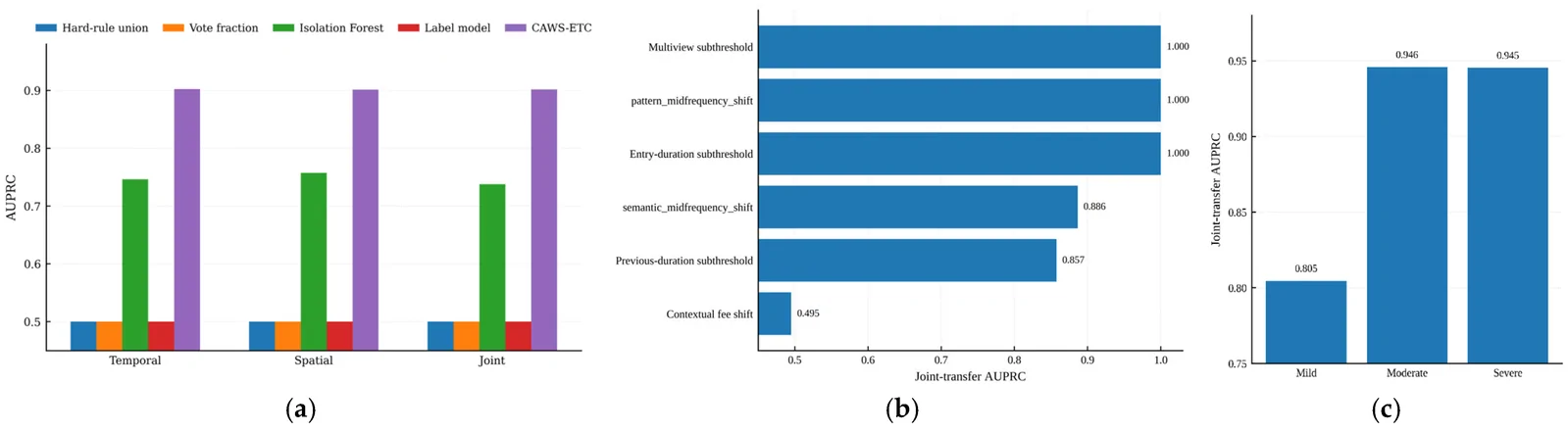
Rule-orthogonal challenge results for the pre-specified evaluation methods: (**a**) method-level AUPRC across transfer settings; (**b**) CAWS-ETC AUPRC by challenge mechanism; and (**c**) AUPRC by intervention severity.

Table 12 reports the method-level rule-orthogonal results across the three transfer conditions and the CAWS-ETC results by intervention mechanism and severity. Figure 6 summarizes these findings by contrasting the methods and displaying the severity gradient.

The post hoc supervision-source attribution analysis first examined whether probabilistic high-confidence selection changed the training references relative to the direct rule states. Across all 14,510,847 transactions, no mismatch was observed in either training eligibility or assigned binary class after application of the original 0.90/0.10 posterior thresholds. Thus, under the observed weak-source configuration, high-confidence probabilistic selection produced the same eligible binary references as the direct rule states.

In the rule-orthogonal challenge, the direct-rule LightGBM achieved AUPRC values of 0.9270, 0.9276, and 0.9280 under temporal, spatial, and joint transfer, respectively. The matched confidence-weighted LightGBM produced corresponding values of 0.9270, 0.9276, and 0.9280. In the joint-transfer subset, the paired AUPRC difference between the matched confidence-weighted model and direct-rule LightGBM was 0.000002 (95% CI: −0.000007 to 0.000011). Under identical records, labels, feature representation, hyperparameters, and model capacity, posterior-confidence weighting therefore produced no measurable incremental ranking benefit.

The same pattern was observed when the post hoc attribution models were evaluated against the 777 determinate manual labels. The direct-rule LightGBM and matched confidence-weighted LightGBM both achieved a sampling-weighted AUROC of 0.7904 and AUPRC of 0.8033. The corresponding paired weighted-AUPRC difference was 0.000000. These manual labels remained evaluation-only and were not used in fitting either attribution model.

The originally fitted CAWS-ETC achieved AUPRC values of 0.9018 on the joint rule-orthogonal challenge and 0.7946 against the determinate manual labels. These point estimates were lower than those of the direct-rule LightGBM. However, the originally fitted CAWS-ETC retained its original training-sample realization, whereas the two post hoc attribution models were fitted on one shared matched sample. The comparison with the original CAWS-ETC is therefore interpreted as a system-level comparison rather than a causal estimate of the effect of probabilistic aggregation. The fully matched direct-rule versus confidence-weighted comparison provides the appropriate component-level attribution.

The feature-family ablation program refitted all variants, including a full-feature reference model, under a common retraining protocol. The resulting full-reference AUPRC of 0.9185 is therefore used only as the within-ablation baseline and should not be numerically conflated with the AUPRC of 0.9018 obtained by the originally fitted CAWS-ETC model in the rule-orthogonal challenge.

Ablation experiments revealed different roles for the feature families. The retrained full model achieved a joint-challenge AUPRC of 0.9185. Removing temporal features reduced this value to 0.8076 and the F1-score from 0.8590 to 0.6739, confirming that temporal context was central to rule-orthogonal generalization. Removing monetary features caused only a modest challenge-set reduction to 0.9123, but it substantially weakened association with the operational proxies: joint-test AUPRC fell from 0.7909 to 0.4753 for transaction failure and from 0.7858 to 0.4761 for nonzero fee-calculation status. Semantic/contextual removal reduced challenge recall and lowered the transaction-failure proxy AUPRC to 0.6861. Structural-feature removal did not reduce challenge ranking, reflecting the absence of array-length inconsistencies and the limited empirical contribution of this family in the present dataset.

The proxy results also clarify why no single feature family should be selected solely by operational-status association. Removing temporal features improved joint-test proxy AUPRC to 0.9847 for transaction failure and 1.0000 for fee-calculation status, while its rule-orthogonal challenge AUPRC decreased to 0.8076. The operational proxies are therefore dominated by fee-related processes and do not represent the complete anomaly construct. A model selected only to maximize proxy agreement would underrepresent temporal and contextual inconsistencies.

Source-balanced anomaly sampling increased joint-challenge recall from 0.7611 to 0.7772 and slightly improved proxy AUPRC, while retaining an overall challenge AUPRC of 0.9161. This supports the use of source balancing as a robustness option, although the present analysis does not directly establish that it resolves the low recall for discount-total mismatch. Confidence thresholds of 0.85/0.15, 0.90/0.10, and 0.95/0.05 produced identical 300,000-record results because the weak probabilities were concentrated in three separate regions. Training-size results were not monotonic: joint-challenge AUPRC values were 0.8910, 0.8656, and 0.9185 for 100,000, 300,000, and 600,000 records. The combined sampling/model-seed variants ranged from 0.8333 to 0.8719 at 300,000 records, and one seed showed a marked decrease in proxy association. Consequently, stochastic robustness should not be overstated.

**Figure 7:**
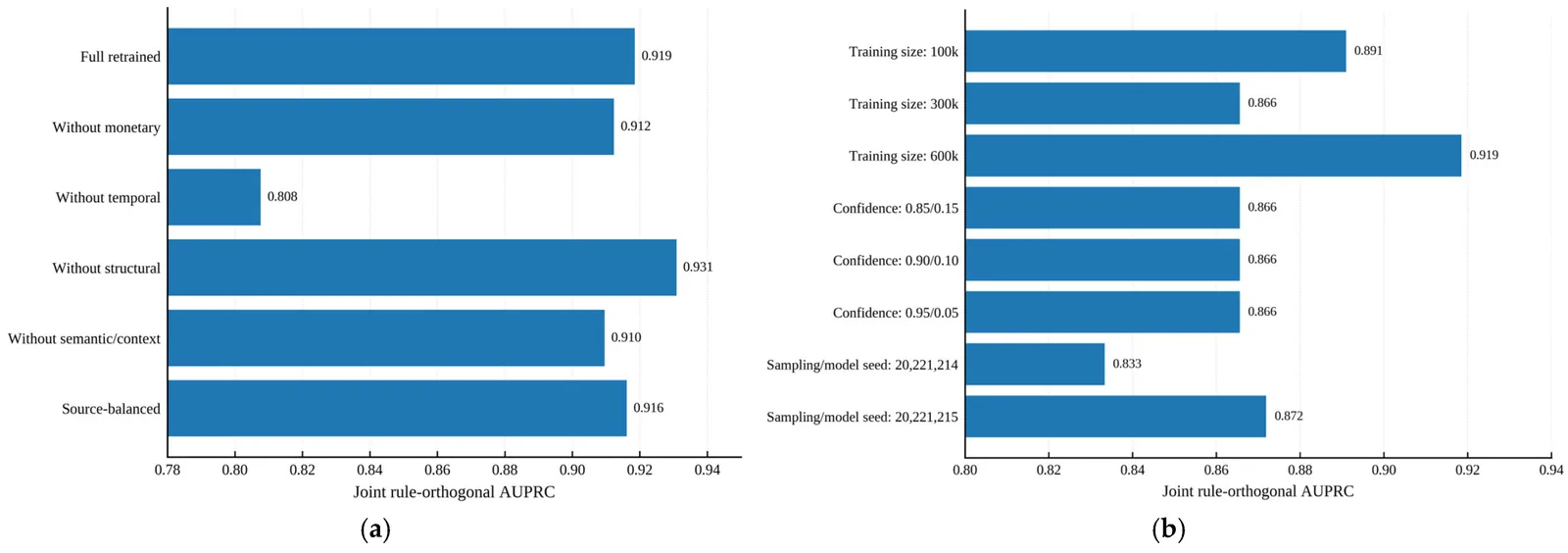
Ablation and sensitivity analysis based on the joint rule-orthogonal challenge AUPRC: (**a**) effects of removing individual feature families and applying source-balanced sampling; and (**b**) sensitivity to training-sample size, pseudo-label confidence thresholds, and combined sampling/model random seeds.

Figure 7 compares the full retrained model with the four feature-family ablations, source-balanced sampling, training-size variants, and the combined sampling/model-seed configurations. Extended challenge, proxy-association, and tie-aware enrichment results are provided in the separate Supplementary Material Table S3.

**Figure 8:**
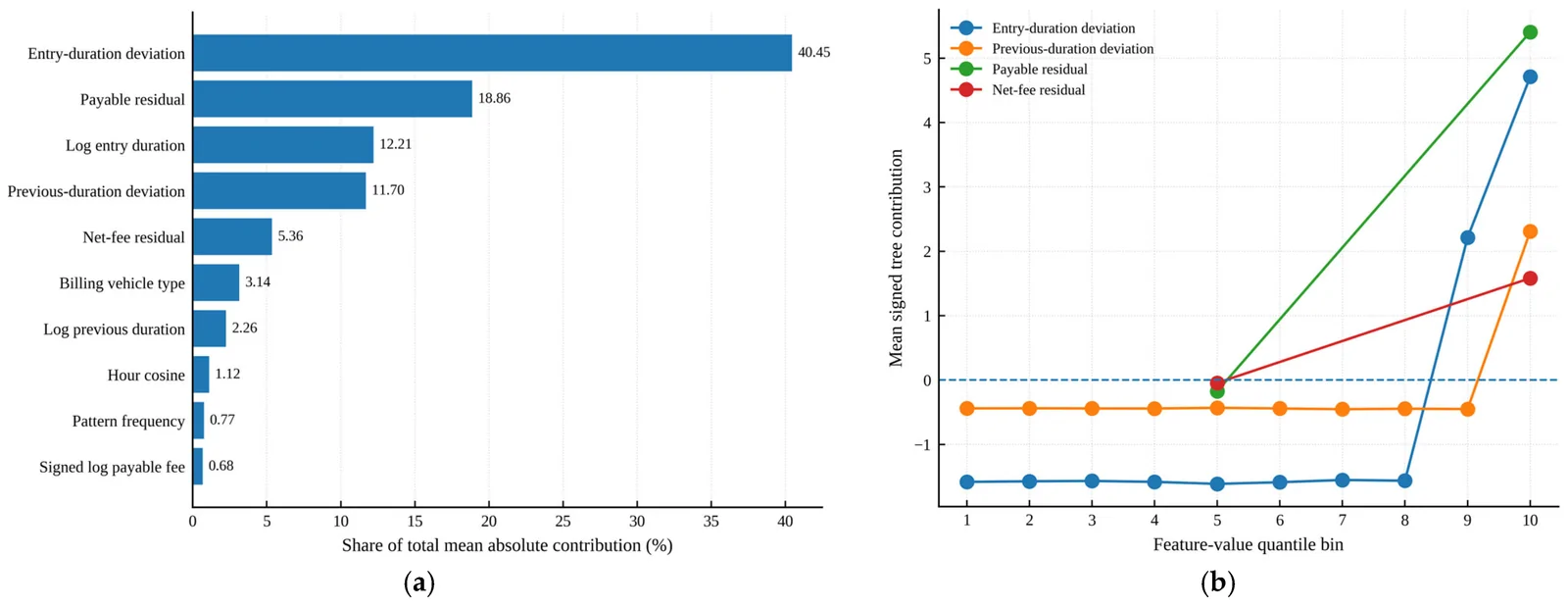
LightGBM tree-contribution analysis: (**a**) normalized global contributions of the ten leading features; and (**b**) binned signed contributions of selected monetary and temporal attributes. In panel (**b**), the horizontal dashed line at zero denotes the reference level of no signed contribution; values above and below zero indicate positive and negative contributions to the CAWS-ETC score, respectively.

Tree-contribution analysis was consistent with the ablation findings. Excluding the expected-value term, entry-duration deviation accounted for 40.45% of the total mean absolute contribution, followed by payable-amount residual (18.86%), log entry duration (12.21%), previous-duration deviation (11.70%), and net-amount residual (5.36%). The contribution of entry-duration deviation changed sharply from negative to positive in the upper two deciles, while nonzero payable and net residuals generated strong positive contributions. These patterns show that the model uses both continuous contextual deviations and deterministic monetary discrepancies. Figure 8 presents the ten features with the largest normalized mean absolute tree contributions and shows the binned signed contribution of the principal continuous attributes.

**Figure 9:**
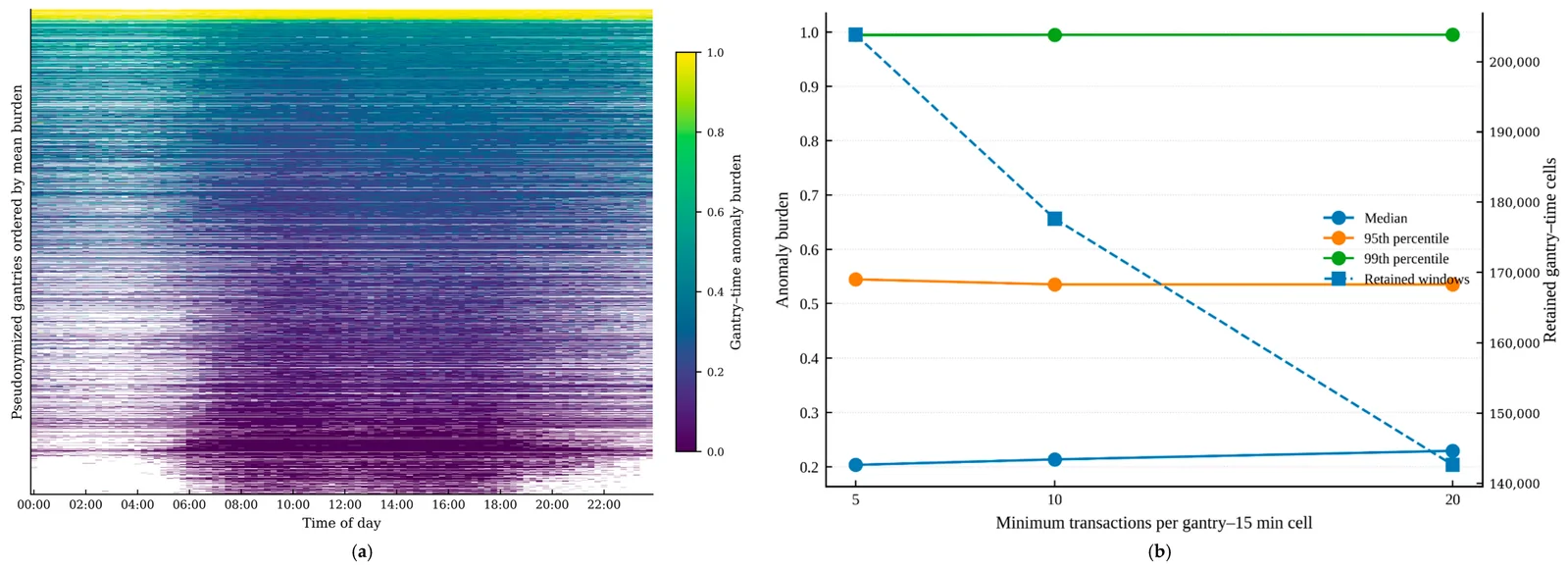
Gantry–time anomaly burden: (**a**) pseudonymized gantry-by-time heatmap for cells containing at least 10 transactions; and (**b**) sensitivity of the burden distribution to minimum cell sizes of 5, 10, and 20 transactions.

The gantry–time aggregation was stable after excluding very low-volume cells. Requiring at least 5, 10, and 20 transactions retained 203,857, 177,651, and 142,614 windows, respectively. The 95th-percentile burden remained between 0.5353 and 0.5446, and the 99th percentile remained near 0.995. A minimum of 10 transactions therefore provides a practical compromise between statistical stability and coverage for the main gantry–time visualization. Figure 9 presents the pseudonymized gantry–time anomaly-burden map after applying the minimum-count threshold of 10 transactions and compares the burden distributions obtained with thresholds of 5, 10, and 20 transactions.

## 5. Discussion

### 5.1. Methodological Findings and Comparison with Alternative Strategies

The principal methodological finding becomes more specific when the supervision-source attribution results are considered. Programmatic consistency supervision can be transferred by a discriminative LightGBM model into a continuous anomaly ranking without requiring manually verified anomaly labels during model construction. However, the ability to rank deviations beyond direct rule activation should not be attributed specifically to probabilistic weak-label aggregation. After high-confidence selection, the probabilistic and direct rule-created binary references were exactly equivalent in the present dataset, and the matched confidence-weighted and unweighted LightGBM models were nearly indistinguishable. The observed gain over non-learning hard-rule and vote-based scores therefore arose primarily from the discriminative transfer of rule-created supervision through continuous and multiview model features.

The post hoc attribution analysis does not replace the blinded manual audit, which remains the principal real-record validation of observable transaction inconsistencies. The two analyses answer different questions: manual adjudication evaluates whether the resulting ranking corresponds to verifiable abnormalities in unmodified real transactions, whereas the direct-rule comparison examines which training component generated the ranking behavior. Together, they show that real-record prioritization can be achieved without demonstrating an incremental ranking advantage from the probabilistic aggregation layer itself.

The comparison with the originally fitted CAWS-ETC requires a separate interpretation. Direct-rule LightGBM yielded higher AUPRC point estimates in the rule-orthogonal and manual-label evaluations, but the originally fitted CAWS-ETC retained its original training-sample realization. This difference therefore does not establish that probabilistic aggregation is detrimental. The fully matched comparison is the appropriate component-level test, and that comparison showed a negligible effect of posterior-confidence weighting. In the present dataset, the probabilistic layer should therefore be viewed primarily as a structured mechanism for source integration and uncertainty representation rather than as a demonstrated source of incremental ranking performance.

The four consistency views played complementary roles. Temporal context supplied the strongest generalization signal for subthreshold and multiview deviations. Monetary consistency was the dominant contributor to association with transaction failure and fee-calculation status. Semantic and missingness features improved the coverage of less frequent patterns, whereas the structural family contributed little in this particular dataset because no component-length mismatch was observed. The low incremental contribution of a family should not be interpreted as evidence that it is universally unnecessary; rather, it reflects the empirical error mechanisms present on the observation day.

The parser stress test further clarifies the boundary between deterministic safeguards and learned anomaly sensing. A predefined parser check can trigger perfectly when its Boolean condition is synthetically activated even if that condition never occurred in the fitting data; this is an execution property of the rule rather than evidence of statistical generalization. The probabilistic label model was indirectly affected because the parser intervention removed joint-normal evidence, despite exclusion of the zero-coverage parser anomaly source itself. By contrast, CAWS-ETC showed chance-level parser ranking because no empirical parser-error support was available from which the discriminator could learn a corresponding response. The experiment therefore supports retaining explicit integrity safeguards for structurally definable failure modes that are absent from the observed training distribution, while distinguishing such safeguards from learned anomaly-sensing capability.

The results also demonstrate the limitations of using operational fields as surrogate ground truth. Removing temporal features produced nearly perfect fee-related proxy association, despite reducing performance on the independent rule-orthogonal challenge. This divergence indicates that the proxies capture only part of the target construct. Transaction failure and fee-calculation status are closely related to monetary processing, while a broader sensing framework must also detect temporal, semantic, and structural irregularities. For this reason, the proxy analysis was used as independent enrichment evidence rather than as the sole model-selection criterion.

The comparison with Isolation Forest became more nuanced after manual validation. CAWS-ETC clearly outperformed the unsupervised baseline on the rule-aligned and rule-orthogonal controlled challenges, indicating stronger recovery of consistency mechanisms represented during weak supervision. This advantage, however, did not extend uniformly to the manually adjudicated real-record subset. Isolation Forest achieved higher sampling-weighted AUROC and AUPRC point estimates against the determinate manual labels, whereas CAWS-ETC provided higher precision and specificity at the frozen validation threshold. Specifically, Isolation Forest achieved perfect recall but a specificity of only 0.4438, whereas CAWS-ETC achieved a recall of 0.8016 together with a specificity of 0.8036. All manually confirmed anomalies in this subset were temporal inconsistencies. These findings suggest that isolation-based and consistency-aware weakly supervised models capture partly different anomaly structures and that comparative superiority should not be generalized across anomaly mechanisms.

The high-confidence weak-label fit should therefore be interpreted as knowledge transfer rather than conventional supervised accuracy. The output score is suitable for ranking and workload prioritization, but it is not calibrated as the probability of fraud, toll evasion, or equipment failure. This distinction also explains why tie-aware selection is necessary: several transactions can receive identical high scores because they share the same strong consistency pattern.

### 5.2. Practical Implications, Limitations, and Future Research

The proposed framework can support transaction-data auditing in three ways. First, it converts heterogeneous consistency indicators into a common ranking scale, enabling analysts to review the most informative records first. Second, the gantry–time burden aggregates transaction-level evidence without exposing vehicle identifiers and can reveal periods requiring further inspection. Third, the implementation is computationally feasible on a conventional computer: probabilistic label inference, LightGBM training, full-data scoring, and the complete robustness analysis were completed without a GPU.

For engineering deployment, CAWS-ETC should initially be implemented as a local decision-support layer rather than an autonomous adjudication module. A practical workflow can follow five steps. First, each incoming ETC data batch should undergo the same schema, duplicate, timestamp, monetary-component, and array-integrity checks used in model development, while direct identifiers remain in a protected local linkage environment and model-facing gantry identifiers are pseudonymized. Second, contextual reference statistics and weak-source definitions should be estimated from a designated local reference period and frozen before scoring the subsequent operational batch. Third, the validated LightGBM model can generate transaction-level scores, which can then be aggregated into pseudonymized gantry–time burdens. Fourth, review queues should be constructed using tie-aware high-score cutoffs or workload-defined top-percentile bands rather than interpreting the score as a calibrated probability of fraud or equipment failure. Fifth, analysts should inspect prioritized transactions using the available transaction context and, where available, independent operational evidence; confirmed cases may subsequently support retraining or recalibration only after a separate validation procedure. Weak-source coverage, score distributions, processing failures, and gantry–time burdens should also be monitored for drift. Because the present study evaluated batch processing rather than streaming latency and has not established multi-day or cross-region validity, an initial implementation should be treated as a locally validated audit pilot, with real-time deployment and model updates subject to additional validation.

The tie-aware enrichment results illustrate the potential reduction in manual review. In the joint-transfer condition, reviewing approximately the highest-scoring 1.1% of transactions increased the observed transaction-failure rate by 5.78 times and the nonzero fee-calculation rate by 5.55 times. These gains support prioritization, but they do not authorize automatic adjudication. A high score should trigger data-quality or operational review rather than a direct conclusion about a vehicle, user, or device.

The manual audit provides a more direct indication of practical review prioritization. Among transactions with determinate adjudications, the anomaly rate exceeded 80% throughout the highest approximately 20% of CAWS-ETC scores but decreased to 8.3% in the remaining approximately 80%. This separation indicates that the score can substantially concentrate manually verifiable transaction inconsistencies toward the upper part of the review queue. Nevertheless, the large number of indeterminate records shows that score-based prioritization cannot replace access to richer operational evidence when the transaction table alone is insufficient for adjudication.

Several limitations remain. The principal external-validity limitation is the restricted temporal and geographic scope of the dataset. All model development and evaluation were based on transactions collected during a single 24 h period on 13 December 2022 from one provincial expressway network in Shandong. Although the leakage-resistant design evaluates later within-day periods and held-out gantries, these partitions remain internal holdouts drawn from the same observation day and regional system. They therefore do not establish that the learned contextual reference distributions, weak-source behavior, or anomaly-ranking performance would remain stable on other days, across seasons, or in expressway systems from other regions. Accordingly, the temporal-, spatial-, and joint-transfer results reported in this study should be interpreted as evidence of within-dataset robustness rather than external spatiotemporal generalizability. The added manual audit substantially strengthens real-record validation but remains limited to a score-stratified subset of the joint-transfer data. Of the 1200 reviewed records, 423 remained indeterminate because the available transaction fields were insufficient for a defensible binary judgment, and all 546 confirmed anomalies were categorized as temporal inconsistencies. The manual experiment therefore provides stronger independent evidence for real temporal transaction errors than for monetary, structural, or semantic anomalies. The score-stratified design was corrected using inverse sampling-probability weights, but the estimated 27.9% weighted anomaly proportion applies only to determinate adjudications and should not be interpreted as the anomaly prevalence of the complete joint-transfer population. In addition, the latent-class label model assumes approximate conditional independence among the selected weak sources given the unobserved class. The system-recorded proxies remain association measures rather than ground truth, and no road-network topology, speed, image, device-log, or plate-recognition data were available for resolving ambiguous transactions. The model also remained ineffective for parsing corruption absent from training support and showed chance-level performance for contextual fee shifts.

The robustness analysis also identified sampling sensitivity. One combined sampling/model-seed configuration showed substantially weaker proxy association, and the current experiment changes the sampled records and the model seed simultaneously. A final reproducibility audit should separate these two sources of randomness by holding the sampled records fixed while varying the LightGBM seed, and then holding the model seed fixed while varying only the sample. Until that check is completed, the manuscript should not claim full stochastic invariance.

An additional limitation concerns attribution within the weak-supervision pipeline. In the present dataset, the original 0.90/0.10 posterior thresholds produced exactly the same eligible binary references as the direct rule states, and the matched confidence-weighted and unweighted LightGBM models were nearly indistinguishable. Consequently, the present study does not demonstrate an incremental ranking benefit from probabilistic weak-label aggregation or posterior-confidence weighting under the observed weak-source configuration. These components may become more consequential when weak sources overlap, conflict, or generate less separated posterior states, but this possibility requires evaluation on additional datasets rather than extrapolation from the present case.

The supervision-source attribution analysis was also post hoc. The direct-rule and matched confidence-weighted models shared an identical training sample, allowing their difference to isolate posterior-confidence weighting. In contrast, the originally fitted CAWS-ETC retained its original training-sample realization. Differences between the direct-rule comparator and the original CAWS-ETC should therefore be interpreted as system-level performance differences rather than as causal estimates of the effect of probabilistic aggregation.

External validation is therefore a priority for future research. The present framework and manual-adjudication protocol should be evaluated on multiple nonconsecutive observation dates spanning different seasons and on independent expressway networks from other regions. Such studies should distinguish frozen-model external transfer from any subsequent local recalibration and should use larger adjudicated samples, with particular emphasis on obtaining independent evidence for monetary, structural, and semantic inconsistencies. Additional road-network topology, device-log, image, or plate-recognition information could reduce the proportion of indeterminate records and enable more specific causal diagnosis. A sufficiently large adjudicated dataset could subsequently support explicit score calibration while preserving a separate untouched test set. Active learning could also be used to prioritize uncertain records for expert review, while deterministic safeguards should remain in parallel for failure modes absent from the weak-label training support, particularly parsing errors.

## 6. Conclusions

This study developed CAWS-ETC, a consistency-aware weakly supervised framework for anomaly sensing in large-scale ETC gantry transactions. The method integrates monetary, temporal, structural, and contextual-semantic relationships into weak evidence, estimates probabilistic labels without manually adjudicated anomalies during model development, and transfers this evidence into a lightweight LightGBM ranking model. The complete workflow was applied locally to 14,510,847 transactions from 2525 gantries.

Within the single-day Shandong dataset, CAWS-ETC maintained stable performance across held-out later-period, gantry, and joint-transfer conditions, with rule-aligned AUPRC values of 0.9354–0.9404 and rule-orthogonal values of approximately 0.90. The blinded manual audit provided independent real-record validation: two reviewers achieved 86.0% initial agreement, 168 disagreements were resolved by consensus, and 777 transactions received determinate binary labels. CAWS-ETC achieved a sampling-weighted AUPRC of 0.7946 against these labels, although Isolation Forest produced higher ranking point estimates on the manually confirmed temporal anomalies.

The post hoc supervision-source attribution analysis refined the interpretation of these results. Direct rule-created and high-confidence probabilistic training references were exactly equivalent after the 0.90/0.10 selection, while matched direct-rule and confidence-weighted LightGBM models produced essentially identical rankings. The evidence therefore supports programmatic consistency supervision combined with discriminative LightGBM transfer as the principal source of ranking capability beyond direct rule activation, but it does not establish an incremental performance benefit from probabilistic aggregation or posterior-confidence weighting in the present dataset.

Overall, the resulting score should be interpreted as a transaction-prioritization signal rather than as a calibrated probability of fraud, toll evasion, or equipment failure. However, the transfer results are confined to internal partitions of one observation day and one provincial expressway system and therefore do not establish performance on other days, across seasons, or in other regions. External multi-day, multi-season, and cross-region validation, together with broader manually adjudicated anomaly mechanisms, is required before the generalizability of CAWS-ETC can be established.

## Figures and Tables

**Table 1:** Comparative summary of recent studies relevant to ETC transaction analysis, anomaly detection, and weak supervision from 2021 to 2026.

Ref.	Year	Main Focus	Main Strength	Limitation Relevant to the Present Study
[7] Pang et al.	2021	Deep anomaly detection review	Provides a broad taxonomy of modern anomaly-detection paradigms	General anomaly-detection perspective without ETC-specific consistency knowledge
[8] Ruff et al.	2021	Deep and shallow anomaly detection	Unifies major theoretical perspectives on anomaly detection	Does not address domain rules or ETC transaction relationships
[9] Choi et al.	2021	Time-series anomaly detection	Provides systematic guidance for temporal anomaly modeling	Primarily focuses on time-series structures rather than heterogeneous transaction tables
[2] Zou et al.	2022	ETC-based expressway speed prediction	Demonstrates the value of spatial–temporal ETC information	Targets traffic-state inference rather than transaction-integrity anomalies
[5] Guo et al.	2022	Abnormal ETC data detection using DTW	Incorporates transaction-sequence structure into abnormal-data detection	Focuses on predefined sequential abnormal patterns
[10] Schmidl et al.	2022	Time-series anomaly-detection benchmarking	Provides systematic cross-method evaluation	Benchmark setting does not incorporate ETC-specific business consistency
[11] Han et al.	2022	General anomaly-detection benchmark	Offers broad cross-dataset method comparison	Generic benchmark datasets contain limited domain-specific transaction constraints
[19] Kim et al.	2022	Rigorous anomaly-detection evaluation	Identifies biases caused by evaluation protocols	Focuses mainly on evaluation methodology rather than ETC anomaly construction
[4] Zou et al.	2023	ETC missing-data restoration	Exploits multiple ETC attributes for data-quality recovery	Addresses missing information rather than heterogeneous internal inconsistencies
[1] Shahrier et al.	2024	ETC systems review	Provides comprehensive ETC and intelligent transportation system (ITS) context	Broad system-level review rather than transaction-level anomaly sensing
[3] Lai et al.	2024	ETC vehicle-arrival estimation	Demonstrates temporal inference from successive ETC observations	Assumes sufficiently reliable transaction sequences
[6] Zou et al.	2024	TL-XGBoost ETC anomaly detection	Combines domain structure with supervised tree learning	Requires road-network topology, capture information, and predefined abnormal classes
[20] Sørbø and Ruocco	2024	Anomaly-detection metric taxonomy	Clarifies strengths and biases of evaluation metrics	Focuses on evaluation metrics rather than anomaly-learning mechanisms
[12] Qiu et al.	2025	Self-supervised anomaly detection	Reduces dependence on manually labeled anomaly samples	Does not explicitly encode ETC business-rule relationships
[14] Martínez-Heredia and Ventura	2025	Weak supervision survey	Summarizes weak supervision under scarce verified labels	Focuses on predictive maintenance rather than ETC transaction sensing
[21] Taheri Sarteshnizi et al.	2026	Real-world traffic-volume anomaly detection	Provides visually inspected ground-truth labels and systematic evaluation on real traffic-volume data	Focuses on traffic-volume time series rather than heterogeneous ETC transaction tables and multiview consistency relations
[22] Wang et al.	2026	Weakly supervised tabular anomaly detection	Provides fine-grained and interpretable anomaly localization	Assumes coarse sample-level anomaly labels and generic benchmark supervision

Note: Foundational methodological references published before 2021 are retained in the narrative discussion because they define the underlying algorithms or evaluation concepts. The table focuses on recent literature from 2021 to 2026 as requested.

**Table 2:** Source-field groups and their roles in the CAWS-ETC workflow.

Information Group	Source Fields	Analytical Role	Status in Model Development
Transaction identifiers	trade_id; pass_id	Local duplicate/integrity checks and private record linkage only	Excluded before model development
Gantry information	gantry_id; gantry_order_num; gantry_hex; gantry_hex_opposite; last_gantry_hex	Spatial partitioning, pseudonymized aggregation, and availability indicators	Raw values excluded; stable pseudonyms/indicators retained
Temporal attributes	trans_time; en_time; last_gantry_time	Temporal partitioning, timestamp validity, elapsed-time features, and contextual deviations	Derived validities/durations retained
Monetary and component arrays	pay_fee; discount_fee; toll_interval_id; pay_fee_group; discount_fee_group; fee_group	Component counts/sums, residuals, parsing, and structural-pattern features	Derived features retained
Vehicle and transaction context	media_type; vehicle_plate; vehicle_type; vehicle_class; fee_vehicle_type	Context conditioning, code-pair frequency, plate-color suffix, and semantic consistency	Raw plate deleted; non-identifying/derived attributes retained
Operational-status indicators	trade_result; fee_calc_result; special_type	Independent proxy association and enrichment only	Excluded from features, weak labels, fitting, and calibration

Note: Raw identifiers remained local. Operational-status fields were excluded from model development.

**Table 3:** Data-integrity checks and observed outcomes in the dataset.

Integrity Check	Operational Criterion	Affected Records	Percent of All Records (%)	Analytical Treatment
CSV row structure	Row must match the 24-field schema	0	0.0000	No removal required
Duplicated transaction identifier	Repeated trade_id	0	0.0000	Checked locally; identifier then excluded
Fully duplicated record	Repeated complete-row hash	0	0.0000	No removal required
Entry timestamp before plausibility boundary	en_time < 1 January 2000	197,650	1.3621	Retained through validity indicator
Entry timestamp after observation period	en_time > 13 December 2022 23:59:59	438	0.0030	Retained through validity indicator
Entry timestamp after transaction	en_time > trans_time	475	0.0033	Retained through ordering indicator
Previous timestamp before plausibility boundary	last_gantry_time < 1 January 2000	2,599,253	17.9125	Conditioned on previous-gantry availability
Previous timestamp after observation period	last_gantry_time > 13 December 2022 23:59:59	706	0.0049	Conditioned on previous-gantry availability
Previous timestamp after transaction	last_gantry_time > trans_time	986	0.0068	Retained as conflict evidence
Payable-total mismatch	r_pay ! = 0 when component sum is comparable	78,409	0.5403	Retained as deterministic evidence
Discount-total mismatch	r_discount ! = 0 when component sum is comparable	8620	0.0594	Retained as deterministic evidence
Net-total mismatch	r_net ! = 0 when component sum is comparable	78,203	0.5389	Retained as deterministic evidence
Component-array length mismatch	Comparable interval/pay/discount/net arrays have unequal lengths	0	0.0000	Safeguard retained; no event observed
Missing monetary component arrays	At least one fee-component array unavailable	2,002,946	13.8031	Context-dependent; not a universal anomaly
Recorded/billing vehicle-type disagreement	vehicle_type ! = fee_vehicle_type	2,923,773	20.1489	Modeled through pair frequency; not a universal anomaly

Note: Percentages use 14,510,847 as denominator.

**Table 4:** Spatial–temporal data partitions and analytical roles.

Role	Implementation Code	Spatial Group	Time Period	Primary Purpose	Records	Gantries
Core training	train_core	Train	00:00–17:59	Estimate contextual statistics and fit weak/discriminative models	8,079,105	1768
Temporal validation	validation_temporal	Train	18:00–20:59	Secondary later-period validation on training gantries	1,367,903	1765
Temporal-transfer test	test_temporal	Train	21:00–23:59	Evaluate later within-day transfer on training gantries	779,896	1762
Spatial validation	validation_spatial	Validation	00:00–17:59	Secondary spatial validation during training period	1,765,005	379
Joint validation	validation_joint	Validation	18:00–20:59	Threshold selection and early stopping on held-out gantries and later period	289,517	379
Auxiliary	auxiliary	Validation	21:00–23:59	Retained for completeness; excluded from primary selection	163,093	379
Spatial-transfer test	test_spatial	Test	00:00–17:59	Evaluate transfer to held-out test gantries	1,628,850	378
Auxiliary	auxiliary	Test	18:00–20:59	Retained for completeness; excluded from primary selection	278,582	377
Joint-transfer test	test_joint	Test	21:00–23:59	Evaluate combined spatial and temporal transfer	158,896	377

Note: Contextual statistics were estimated only from the core training subset and then frozen.

**Table 5:** Weak sources entered into the probabilistic label model.

Weak Source	View/Family	Voting Condition	Output	Covered Records	Coverage (%)	Final Status
lf_a_monetary_total	Monetary	Payable or net residual is nonzero when components are comparable	1; otherwise −1	78,482	0.5409	Included
lf_a_discount_mismatch	Discount	Discount residual is nonzero when components are comparable	1; otherwise −1	8620	0.0594	Included
lf_a_entry_invalid	Entry	Entry timestamp violates plausibility or ordering criteria	1; otherwise −1	198,125	1.3654	Included
lf_a_previous_conflict	Previous	Previous gantry is present but previous timestamp is invalid	1; otherwise −1	57,325	0.3950	Included
lf_a_entry_context	Entry	Entry-duration robust deviation ≥ 8	1; otherwise −1	763,727	5.2631	Included
lf_a_previous_context	Previous	Previous-duration robust deviation ≥ 8	1; otherwise −1	367,626	2.5335	Included
lf_a_semantic_rare_pair	Semantic	Vehicle types disagree and training pair count ≤ 20	1; otherwise −1	731	0.0050	Included
lf_a_out_of_dictionary_rare	Out	Rare pair (count ≤ 20) contains an out-of-dictionary code	1; otherwise −1	1143	0.0079	Included
lf_a_rare_missing_pattern	Rare	Rare pattern (count ≤ 20) also contains suspicious evidence	1; otherwise −1	62	0.0004	Included
lf_n_joint_consistent	Joint normal anchor	All four component normal functions vote 0	0; otherwise −1	10,639,650	73.3220	Included

Note: Outputs are coded as −1 for abstention, 0 for normal evidence, and 1 for anomaly evidence. Detailed candidate-source diagnostics are provided in Table S1 of the separate Supplementary Material.

**Table 6:** Probabilistic label-model and LightGBM configuration.

Component	Item	Value	Purpose/Interpretation
Probabilistic label model	Active weak sources	9 anomaly-oriented + 1 joint normal anchor	Operational proxies excluded
Probabilistic label model	Fitting sample	500,000 core-training records	Deterministic sample
Probabilistic label model	Estimation	Expectation-maximization	Conditional-independence latent-class model
Probabilistic label model	Restarts/maximum iterations	3/100	Best observed likelihood retained
Probabilistic label model	Convergence tolerance	1 × 10^−6^	Relative log-likelihood change
Probabilistic label model	Dirichlet pseudocount scale	1.0	Orientation preserving
Pseudo-label selection	Anomaly/normal thresholds	≥0.90/≤0.10	Intermediate records excluded from discriminator training
Pseudo-label selection	Eligible core-training records	682,628 anomaly; 5,970,006 normal	Original thresholds sufficient
LightGBM discriminator	Training sample	600,000 records (180,000 anomaly; 420,000 normal)	Confidence and class-balanced weights
LightGBM discriminator	Validation sample	200,000 joint-validation records (25,251 anomaly; 174,749 normal)	Early stopping
LightGBM discriminator	Feature count	46	Proxy fields and labeling-function outputs excluded
LightGBM discriminator	Boosting/objective	Gradient boosting decision tree (GBDT)/binary	Central processing unit (CPU) execution
LightGBM discriminator	Candidate/selected iterations	2000/157	Validation-based early stopping
LightGBM discriminator	Learning rate/leaves	0.03/31	/
LightGBM discriminator	Minimum observations per leaf	100	/
LightGBM discriminator	Feature subsampling	0.80	/
LightGBM discriminator	L2 regularization/central processing unit (CPU) threads	1.0/4	Graphics processing unit (GPU) not used
Missing/category handling	Numerical/unseen category	NaN retained/__UNK__	No deterministic numerical imputation

**Table 7:** Comparator methods, methodological origins, and evaluation configurations.

Method	Methodological Origin/Representative Introduction Year	Information Used	Fitting Sample/Configuration	Output	Threshold Use	Interpretive Role
Hard-rule union	Study-defined deterministic control (this study)	Binary union of the nine primary anomaly-oriented sources plus the zero-coverage parser safeguard	No fitting	0/1 rule score	F1-maximizing threshold on joint validation	Deterministic rule-recovery positive control
Anomaly-vote fraction	Study-defined deterministic control (this study)	Fraction of the nine primary anomaly-oriented sources plus the parser safeguard voting anomalous	No fitting	Continuous vote fraction	F1-maximizing threshold on joint validation	Unweighted deterministic weak-source positive control
Probabilistic label model	Programmatic weak-supervision formulation, 2017 [13]	Nine active anomaly-oriented sources and joint normal anchor; zero-coverage parser source excluded	500,000 core-training records	Posterior weak-label probability	F1-maximizing threshold on joint validation	Generative weak-supervision comparator
Isolation Forest	2008 [16]	46 features; no weak labels/proxies	100,000 unlabeled core-training records; 200 trees; max_samples = 10,000	Unsupervised anomaly score	F1-maximizing threshold on joint validation	Data-driven unsupervised baseline
CAWS-ETC	Proposed in this study	46 features and high-confidence pseudo-labels	600,000 weighted pseudo-labeled core-training records	LightGBM anomaly-ranking score	F1-maximizing threshold for rule-aligned classification	Proposed method
Direct-rule LightGBM	Study-defined comparator; LightGBM introduced in 2017 [15]	46 features; binary labels created directly from selected rule states	600,000 shared matched records; 180,000 anomaly + 420,000 normal; 157 iterations	LightGBM ranking score	None for attribution analysis	Post hoc test of discriminative transfer from direct rule-created labels
Matched confidence-weighted LightGBM	Study-defined comparator; LightGBM introduced in 2017 [15]	Same 46 features, records, and binary classes; posterior-confidence weighting	Same 600,000 records and 157-iteration configuration	LightGBM ranking score	None for attribution analysis	Post hoc isolation of posterior-confidence weighting

Note: For canonical named algorithms, the representative introduction year follows the cited original or foundational publication. Hard-rule union, anomaly-vote fraction, CAWS-ETC, and the two post hoc LightGBM variants are study-defined methods or controls and therefore do not have an independent historical emergence year.

**Table 8:** Dataset audit, spatial partition, temporal partition, and analytical-role statistics. (A) Dataset audit statistics. (B) Spatial and analytical-role distribution.

**(A)**		
**Statistic**	**Value**	**Unit/Interpretation**
Observation date	13 December 2022	/
Observation period	00:00:00–23:59:59	/
Transactions	14,510,847	records
Distinct gantries	2525	gantries
Distinct transaction identifiers	14,510,847	identifiers
Malformed rows	0	records
Payable-total mismatches	78,409	records
Discount-total mismatches	8620	records
Net-total mismatches	78,203	records
Entry timestamps before 2000	197,650	records
Previous timestamps before 2000	2,599,253	records
**(B)**		
**Statistic**	**Count**	**Share of Total (%)**
Training gantries	1768	70.0200
Validation gantries	379	15.0100
Test gantries	378	14.9700
Core training records	8,079,105	55.6800
Joint validation records	289,517	1.9950
Temporal-transfer test records	779,896	5.3740
Spatial-transfer test records	1,628,850	11.2250
Joint-transfer test records	158,896	1.0950
Auxiliary records	441,675	3.0440

**Table 9:** Weak-label model, discriminator training, and complete-data inference diagnostics.

Component	Diagnostic	Value	Interpretation/Unit
Latent label model	Selected weak sources	10	9 anomaly sources + joint normal anchor
Latent label model	Converged restarts	3/3	58 iterations each
Latent label model	Estimated latent anomaly-class prevalence	0.1852	Model parameter; not observed anomaly rate
Weak-evidence states	Joint normal anchor	10,639,650	73.322%
Weak-evidence states	At least one anomaly vote	1,250,971	8.621%
Weak-evidence states	Weakly ambiguous	2,620,226	18.057%
Pseudo-label candidates	High-confidence anomaly	682,628	Core training, *p*~ ≥ 0.90
Pseudo-label candidates	High-confidence normal	5,970,006	Core training, *p*~ ≤ 0.10
Discriminator training	Training sample	600,000	180,000 anomaly + 420,000 normal
Discriminator training	Joint-validation sample	200,000	25,251 anomaly + 174,749 normal
Discriminator training	Selected boosting iteration	157	/
Internal weak-label agreement	Validation AUROC	1.0000	Implementation diagnostic
Internal weak-label agreement	Validation AUPRC	1.0000	Implementation diagnostic
Internal weak-label agreement	Validation log loss	0.0055	Against weak labels
Complete-data inference	Transactions scored	14,510,847	/
Complete-data inference	Gantry-15 min cells	233,476	/
Runtime	Weak-label learning/scoring	61.3880	s
Runtime	LightGBM training	16.8800	s
Runtime	Complete-data scoring/aggregation	87.3430	s

**Table 10:** Rule-aligned intervention performance under temporal, spatial, and joint transfer.

Test Subset	Method	Records	AUROC	AUPRC	Precision	Recall	F1	FPR	TPR at 1% FPR
Temporal-transfer test	Hard-rule union	108,000	1.0000	1.0000	1.0000	1.0000	1.0000	0.0000	1.0000
Temporal-transfer test	Anomaly-vote fraction	108,000	1.0000	1.0000	1.0000	1.0000	1.0000	0.0000	1.0000
Temporal-transfer test	Probabilistic label model	108,000	1.0000	1.0000	1.0000	1.0000	1.0000	0.0000	1.0000
Temporal-transfer test	Isolation Forest	108,000	0.7490	0.7718	0.5642	0.8849	0.6890	0.6836	0.1231
Temporal-transfer test	CAWS-ETC	108,000	0.9090	0.9361	0.9641	0.8052	0.8776	0.0299	0.6815
Spatial-transfer test	Hard-rule union	108,000	1.0000	1.0000	1.0000	1.0000	1.0000	0.0000	1.0000
Spatial-transfer test	Anomaly-vote fraction	108,000	1.0000	1.0000	1.0000	1.0000	1.0000	0.0000	1.0000
Spatial-transfer test	Probabilistic label model	108,000	1.0000	1.0000	1.0000	1.0000	1.0000	0.0000	1.0000
Spatial-transfer test	Isolation Forest	108,000	0.7438	0.7686	0.6519	0.6982	0.6743	0.3728	0.1218
Spatial-transfer test	CAWS-ETC	108,000	0.9158	0.9404	0.9797	0.7665	0.8601	0.0159	0.7078
Joint-transfer test	Hard-rule union	108,000	1.0000	1.0000	1.0000	1.0000	1.0000	0.0000	1.0000
Joint-transfer test	Anomaly-vote fraction	108,000	1.0000	1.0000	1.0000	1.0000	1.0000	0.0000	1.0000
Joint-transfer test	Probabilistic label model	108,000	1.0000	1.0000	1.0000	1.0000	1.0000	0.0000	1.0000
Joint-transfer test	Isolation Forest	108,000	0.7476	0.7609	0.5680	0.8731	0.6883	0.6640	0.0921
Joint-transfer test	CAWS-ETC	108,000	0.9079	0.9354	0.9586	0.8087	0.8773	0.0349	0.6833

Note: The hard-rule union and anomaly-vote fraction are deterministic positive controls. During controlled evaluation, both retained the zero-coverage parser safeguard; their perfect parser-stress recovery therefore reflects direct rule activation rather than learned training support. The probabilistic label model excluded the parser anomaly source but remained sensitive to the parser intervention because the induced parse-error state removed the joint-normal anchor vote.

**Table 11:** Independent blinded manual adjudication and real-record validation using the pre-specified evaluation methods. (A) Manual adjudication outcomes. (B) Sampling-weighted performance against determinate manual labels.

**(A)**						
**Item**			**Result**			
Reviewed unmodified transactions			1200			
Initial exact agreement			1032 (86.0%)			
Three-category Cohen’s κ			0.786			
Consensus-resolved disagreements			168			
Final anomaly			546			
Final normal			231			
Final indeterminate			423			
Determinate binary records			777			
Confirmed anomaly category			Temporal, 546/546			
**(B)**						
**Method**	**AUROC (95% CI)**	**AUPRC (95% CI)**	**Precision**	**Recall**	**F1**	**Specificity**
Hard-rule union	0.5494 (0.5300–0.5723)	0.3124 (0.2780–0.3522)	0.4658	0.1779	0.2574	0.9210
Vote fraction	0.5497 (0.5290–0.5742)	0.3148 (0.2829–0.3555)	0.4658	0.1779	0.2574	0.9210
Probabilistic label model	0.7985 (0.7500–0.8527)	0.5410 (0.4835–0.6085)	0.6483	0.8016	0.7168	0.8316
Isolation Forest	0.9687 (0.9544–0.9811)	0.8665 (0.8185–0.9125)	0.4105	1.0000	0.5821	0.4438
CAWS-ETC	0.7901 (0.7061–0.8868)	0.7946 (0.7442–0.8599)	0.6126	0.8016	0.6944	0.8036

## Data Availability

The data presented in this study are available from the corresponding author upon reasonable request, as the dataset will be used in future research.

## Supplementary Materials

The following supporting information can be downloaded at: https://www.mdpi.com/article/10.3390/s26185889/s1, Table S1: (A) Complete candidate labeling functions and empirical coverage. (B) Pairwise labeling-function overlap and conflict diagnostics; Table S2: (A) Full rule-aligned overall metrics by test role and method. (B) Rule-aligned metrics by role, method, and intervention mechanism. (C) Rule-aligned metrics by role, method, and severity. (D) Low-prevalence stress tests in the joint-transfer subset. (E) Validation-selected thresholds and validation metrics; Table S3: (A) Retrained variant configurations and internal weak-label diagnostics. (B) Full variant performance on the rule-orthogonal challenge. (C) Full variant association with independent operational proxies. (D) Tie-aware enrichment for all methods, roles, and proxies; Table S4: (A) Complete 46-feature input dictionary. (B) Global LightGBM tree-contribution summary. (C) Binned signed tree contributions. (D) Gantry-time burden sensitivity to minimum cell size; Table S5: (A) Weak-label posterior distributions by analytical role. (B) CAWS-ETC score distributions by analytical role. (C) Computational timings. (D) Software requirements specified by the workflow; Table S6: Independent proxy association and tie-aware high-score enrichment; Table S7: Independent proxy association and tie-aware high-score enrichment. (The parser safeguard had zero empirical coverage and was excluded from probabilistic anomaly-source fitting. It was nevertheless retained in the deterministic hard-rule and vote-fraction comparators. The parser intervention also removed the monetary-normal component of the joint normal anchor; consequently, label-model separation of parser-control pairs reflects loss of normal-anchor evidence rather than learning from parser-error examples); Figure S1: Tie-aware high-score enrichment in the joint-transfer subset: (a) requested and achieved high-score fractions after retaining all records tied at the selection cutoff; and (b) enrichment lifts for transaction failure, special-transaction status, and nonzero fee-calculation status at the inclusive Top-1% and Top-5% cutoffs.
