# Supplementary material for: Consistency-Aware Weakly Supervised Anomaly Sensing for Large-Scale Expressway ETC Gantry Transactions
Source: Sensors (Basel). 2026 Sep 17;26(18):5889. doi: 10.3390/s26185889 (PMC13611106; doi:10.3390/s26185889)
Supplement: Supplementary file 1 [file sensors-26-05889-s001.zip › sensors-4496026-supplementary.pdf]

Supplementary Materials: Tables S1–S7

Table S1A. Complete candidate labeling functions and empirical coverage.

Labeling function	Family	Polarity	Definition	Abstain count	Normal votes	Anomaly votes	Coverage (%)	Final use
lf_a_monetary_total	monetary	anomaly	Anomaly vote when payable or net-fee totals disagree with available components.	14,432,365	0	78,482	0.5409	Active anomaly source
lf_a_discount_mismatch	discount	anomaly	Anomaly vote when the total discount disagrees with its components.	14,502,227	0	8,620	0.0594	Active anomaly source
lf_a_group_parse	group	anomaly	Anomaly vote for a non-missing component array that cannot be parsed.	14,510,847	0	0	0.0000	Safeguard; excluded from latent model (zero coverage)
lf_a_entry_invalid	entry	anomaly	Anomaly vote for an entry timestamp outside the plausible interval or after the transaction.	14,312,722	0	198,125	1.3654	Active anomaly source
lf_a_previous_conflict	previous	anomaly	Anomaly vote when a previous gantry is present but its timestamp is invalid.	14,453,522	0	57,325	0.3950	Active anomaly source
lf_a_entry_context	entry	anomaly	Anomaly vote for an extreme entry-duration robust deviation.	13,747,120	0	763,727	5.2631	Active anomaly source
lf_a_previous_context	previous	anomaly	Anomaly vote for an extreme previous-gantry-duration robust deviation.	14,143,221	0	367,626	2.5335	Active anomaly source
lf_a_semantic_rare_pair	semantic	anomaly	Anomaly vote for a rare original/billing vehicle-type disagreement.	14,510,116	0	731	0.0050	Active anomaly source
lf_a_out_of_dictionary_rare	out	anomaly	Anomaly vote for a rare vehicle-code pair containing an out-of-dictionary code.	14,509,704	0	1,143	0.0079	Active anomaly source
lf_a_rare_missing_pattern	rare	anomaly	Anomaly vote for a rare missingness/validity pattern with suspicious evidence.	14,510,785	0	62	0.0004	Active anomaly source
lf_n_monetary_consistent	monetary	normal	Normal vote when all monetary arrays are available, parseable, aligned, and exactly consistent.	2,081,428	12,429,419	0	85.6561	Component normal source used only for joint anchor
lf_n_temporal_consistent	temporal	normal	Normal vote when timestamp relationships are valid and duration deviations are non-extreme.	2,387,230	12,123,617	0	83.5487	Component normal source used only for joint anchor
lf_n_common_semantic	common	normal	Normal vote for a vehicle-type combination frequently observed in the training reference.	15,035	14,495,812	0	99.8964	Component normal source used only for joint anchor
lf_n_common_pattern	common	normal	Normal vote for a common missingness/validity pattern in the training reference.	33,397	14,477,450	0	99.7698	Component normal source used only for joint anchor

Table S1B. Pairwise labeling-function overlap and conflict diagnostics.

Left source	Right source	Both covered	Agreements	Conflicts	Overlap (%)	Conflict among overlap (%)
lf_a_monetary_total	lf_a_discount_mismatch	8,620	8,620	0	0.0594	0.0000
lf_a_monetary_total	lf_a_group_parse	0	0	0	0.0000	0.0000
lf_a_monetary_total	lf_a_entry_invalid	0	0	0	0.0000	0.0000
lf_a_monetary_total	lf_a_previous_conflict	0	0	0	0.0000	0.0000
lf_a_monetary_total	lf_a_entry_context	4,582	4,582	0	0.0316	0.0000
lf_a_monetary_total	lf_a_previous_context	6,907	6,907	0	0.0476	0.0000
lf_a_monetary_total	lf_a_semantic_rare_pair	0	0	0	0.0000	0.0000
lf_a_monetary_total	lf_a_out_of_dictionary_rare	0	0	0	0.0000	0.0000
lf_a_monetary_total	lf_a_rare_missing_pattern	10	10	0	0.0001	0.0000
lf_a_monetary_total	lf_n_monetary_consistent	0	0	0	0.0000	0.0000
lf_a_monetary_total	lf_n_temporal_consistent	60,434	0	60,434	0.4165	100.0000
lf_a_monetary_total	lf_n_common_semantic	78,438	0	78,438	0.5405	100.0000
lf_a_monetary_total	lf_n_common_pattern	78,470	0	78,470	0.5408	100.0000
lf_a_discount_mismatch	lf_a_group_parse	0	0	0	0.0000	0.0000
lf_a_discount_mismatch	lf_a_entry_invalid	0	0	0	0.0000	0.0000
lf_a_discount_mismatch	lf_a_previous_conflict	0	0	0	0.0000	0.0000
lf_a_discount_mismatch	lf_a_entry_context	1,125	1,125	0	0.0078	0.0000
lf_a_discount_mismatch	lf_a_previous_context	2,362	2,362	0	0.0163	0.0000
lf_a_discount_mismatch	lf_a_semantic_rare_pair	0	0	0	0.0000	0.0000
lf_a_discount_mismatch	lf_a_out_of_dictionary_rare	0	0	0	0.0000	0.0000
lf_a_discount_mismatch	lf_a_rare_missing_pattern	10	10	0	0.0001	0.0000
lf_a_discount_mismatch	lf_n_monetary_consistent	0	0	0	0.0000	0.0000
lf_a_discount_mismatch	lf_n_temporal_consistent	4,607	0	4,607	0.0317	100.0000
lf_a_discount_mismatch	lf_n_common_semantic	8,613	0	8,613	0.0594	100.0000
lf_a_discount_mismatch	lf_n_common_pattern	8,609	0	8,609	0.0593	100.0000
lf_a_group_parse	lf_a_entry_invalid	0	0	0	0.0000	0.0000
lf_a_group_parse	lf_a_previous_conflict	0	0	0	0.0000	0.0000
lf_a_group_parse	lf_a_entry_context	0	0	0	0.0000	0.0000

lf_a_group_parse	lf_a_previous_context	0	0	0	0.0000	0.0000
lf_a_group_parse	lf_a_semantic_rare_pair	0	0	0	0.0000	0.0000
lf_a_group_parse	lf_a_out_of_dictionary_rare	0	0	0	0.0000	0.0000
lf_a_group_parse	lf_a_rare_missing_pattern	0	0	0	0.0000	0.0000
lf_a_group_parse	lf_n_monetary_consistent	0	0	0	0.0000	0.0000
lf_a_group_parse	lf_n_temporal_consistent	0	0	0	0.0000	0.0000
lf_a_group_parse	lf_n_common_semantic	0	0	0	0.0000	0.0000
lf_a_group_parse	lf_n_common_pattern	0	0	0	0.0000	0.0000
lf_a_entry_invalid	lf_a_previous_conflict	44,268	44,268	0	0.3051	0.0000
lf_a_entry_invalid	lf_a_entry_context	0	0	0	0.0000	0.0000
lf_a_entry_invalid	lf_a_previous_context	872	872	0	0.0060	0.0000
lf_a_entry_invalid	lf_a_semantic_rare_pair	108	108	0	0.0007	0.0000
lf_a_entry_invalid	lf_a_out_of_dictionary_rare	484	484	0	0.0033	0.0000
lf_a_entry_invalid	lf_a_rare_missing_pattern	29	29	0	0.0002	0.0000
lf_a_entry_invalid	lf_n_monetary_consistent	2,998	0	2,998	0.0207	100.0000
lf_a_entry_invalid	lf_n_temporal_consistent	0	0	0	0.0000	0.0000
lf_a_entry_invalid	lf_n_common_semantic	197,006	0	197,006	1.3576	100.0000
lf_a_entry_invalid	lf_n_common_pattern	186,087	0	186,087	1.2824	100.0000
lf_a_previous_conflict	lf_a_entry_context	7,707	7,707	0	0.0531	0.0000
lf_a_previous_conflict	lf_a_previous_context	0	0	0	0.0000	0.0000
lf_a_previous_conflict	lf_a_semantic_rare_pair	223	223	0	0.0015	0.0000
lf_a_previous_conflict	lf_a_out_of_dictionary_rare	588	588	0	0.0041	0.0000
lf_a_previous_conflict	lf_a_rare_missing_pattern	26	26	0	0.0002	0.0000
lf_a_previous_conflict	lf_n_monetary_consistent	7,062	0	7,062	0.0487	100.0000
lf_a_previous_conflict	lf_n_temporal_consistent	0	0	0	0.0000	0.0000
lf_a_previous_conflict	lf_n_common_semantic	56,086	0	56,086	0.3865	100.0000
lf_a_previous_conflict	lf_n_common_pattern	36,749	0	36,749	0.2533	100.0000
lf_a_entry_context	lf_a_previous_context	152,106	152,106	0	1.0482	0.0000
lf_a_entry_context	lf_a_semantic_rare_pair	147	147	0	0.0010	0.0000
lf_a_entry_context	lf_a_out_of_dictionary_rare	456	456	0	0.0031	0.0000

lf_a_entry_context	lf_a_rare_missing_pattern	6	6	0	0.0000	0.0000
lf_a_entry_context	lf_n_monetary_consistent	523,512	0	523,512	3.6077	100.0000
lf_a_entry_context	lf_n_temporal_consistent	0	0	0	0.0000	0.0000
lf_a_entry_context	lf_n_common_semantic	761,036	0	761,036	5.2446	100.0000
lf_a_entry_context	lf_n_common_pattern	752,736	0	752,736	5.1874	100.0000
lf_a_previous_context	lf_a_semantic_rare_pair	57	57	0	0.0004	0.0000
lf_a_previous_context	lf_a_out_of_dictionary_rare	197	197	0	0.0014	0.0000
lf_a_previous_context	lf_a_rare_missing_pattern	34	34	0	0.0002	0.0000
lf_a_previous_context	lf_n_monetary_consistent	250,198	0	250,198	1.7242	100.0000
lf_a_previous_context	lf_n_temporal_consistent	0	0	0	0.0000	0.0000
lf_a_previous_context	lf_n_common_semantic	365,861	0	365,861	2.5213	100.0000
lf_a_previous_context	lf_n_common_pattern	363,746	0	363,746	2.5067	100.0000
lf_a_semantic_rare_pair	lf_a_out_of_dictionary_rare	299	299	0	0.0021	0.0000
lf_a_semantic_rare_pair	lf_a_rare_missing_pattern	2	2	0	0.0000	0.0000
lf_a_semantic_rare_pair	lf_n_monetary_consistent	334	0	334	0.0023	100.0000
lf_a_semantic_rare_pair	lf_n_temporal_consistent	298	0	298	0.0021	100.0000
lf_a_semantic_rare_pair	lf_n_common_semantic	0	0	0	0.0000	0.0000
lf_a_semantic_rare_pair	lf_n_common_pattern	342	0	342	0.0024	100.0000
lf_a_out_of_dictionary_rare	lf_a_rare_missing_pattern	3	3	0	0.0000	0.0000
lf_a_out_of_dictionary_rare	lf_n_monetary_consistent	0	0	0	0.0000	0.0000
lf_a_out_of_dictionary_rare	lf_n_temporal_consistent	15	0	15	0.0001	100.0000
lf_a_out_of_dictionary_rare	lf_n_common_semantic	0	0	0	0.0000	0.0000
lf_a_out_of_dictionary_rare	lf_n_common_pattern	864	0	864	0.0060	100.0000
lf_a_rare_missing_pattern	lf_n_monetary_consistent	32	0	32	0.0002	100.0000
lf_a_rare_missing_pattern	lf_n_temporal_consistent	1	0	1	0.0000	100.0000
lf_a_rare_missing_pattern	lf_n_common_semantic	57	0	57	0.0004	100.0000
lf_a_rare_missing_pattern	lf_n_common_pattern	0	0	0	0.0000	0.0000
lf_n_monetary_consistent	lf_n_temporal_consistent	10,645,033	10,645,033	0	73.3591	0.0000
lf_n_monetary_consistent	lf_n_common_semantic	12,422,674	12,422,674	0	85.6096	0.0000
lf_n_monetary_consistent	lf_n_common_pattern	12,421,587	12,421,587	0	85.6021	0.0000

lf_n_temporal_consistent	lf_n_common_semantic	12,114,243	12,114,243	0	83.4841	0.0000
lf_n_temporal_consistent	lf_n_common_pattern	12,120,314	12,120,314	0	83.5259	0.0000
lf_n_common_semantic	lf_n_common_pattern	14,463,737	14,463,737	0	99.6753	0.0000

Table S2A. Full rule-aligned overall metrics by test role and method.

Role	Method	Records	Threshold	AUROC	AUPRC	Precision	Recall	F1	Specificity	FPR	TPR@1% FPR	AUROC CI low	AUROC CI high	AUPRC CI low	AUPRC CI high	F1 CI low	F1 CI high
test_temporal	Hard-rule union	108,000	1.0000	1.0000	1.0000	1.0000	1.0000	1.0000	1.0000	0.0000	1.0000	1.0000	1.0000	1.0000	1.0000	1.0000	1.0000
test_temporal	Anomaly-vote fraction	108,000	0.1000	1.0000	1.0000	1.0000	1.0000	1.0000	1.0000	0.0000	1.0000	1.0000	1.0000	1.0000	1.0000	1.0000	1.0000
test_temporal	Probabilistic label model	108,000	0.5686	1.0000	1.0000	1.0000	1.0000	1.0000	1.0000	0.0000	1.0000	1.0000	1.0000	1.0000	1.0000	1.0000	1.0000
test_temporal	Isolation Forest	108,000	0.5071	0.7490	0.7718	0.5642	0.8849	0.6890	0.3164	0.6836	0.1231	0.7426	0.7515	0.7683	0.7769	0.6835	0.6894
test_temporal	CAWS-ETC	108,000	0.0071	0.9090	0.9361	0.9641	0.8052	0.8776	0.9701	0.0299	0.6815	0.9058	0.9111	0.9339	0.9377	0.8748	0.8809
test_spatial	Hard-rule union	108,000	1.0000	1.0000	1.0000	1.0000	1.0000	1.0000	1.0000	0.0000	1.0000	1.0000	1.0000	1.0000	1.0000	1.0000	1.0000
test_spatial	Anomaly-vote fraction	108,000	0.1000	1.0000	1.0000	1.0000	1.0000	1.0000	1.0000	0.0000	1.0000	1.0000	1.0000	1.0000	1.0000	1.0000	1.0000
test_spatial	Probabilistic label model	108,000	0.5686	1.0000	1.0000	1.0000	1.0000	1.0000	1.0000	0.0000	1.0000	1.0000	1.0000	1.0000	1.0000	1.0000	1.0000
test_spatial	Isolation Forest	108,000	0.5071	0.7438	0.7686	0.6519	0.6982	0.6743	0.6272	0.3728	0.1218	0.7407	0.7495	0.7656	0.7753	0.6700	0.6781
test_spatial	CAWS-ETC	108,000	0.0071	0.9158	0.9404	0.9797	0.7665	0.8601	0.9841	0.0159	0.7078	0.9124	0.9171	0.9378	0.9412	0.8550	0.8618
test_joint	Hard-rule union	108,000	1.0000	1.0000	1.0000	1.0000	1.0000	1.0000	1.0000	0.0000	1.0000	1.0000	1.0000	1.0000	1.0000	1.0000	1.0000
test_joint	Anomaly-vote fraction	108,000	0.1000	1.0000	1.0000	1.0000	1.0000	1.0000	1.0000	0.0000	1.0000	1.0000	1.0000	1.0000	1.0000	1.0000	1.0000
test_joint	Probabilistic label model	108,000	0.5686	1.0000	1.0000	1.0000	1.0000	1.0000	1.0000	0.0000	1.0000	1.0000	1.0000	1.0000	1.0000	1.0000	1.0000
test_joint	Isolation Forest	108,000	0.5071	0.7476	0.7609	0.5680	0.8731	0.6883	0.3360	0.6640	0.0921	0.7423	0.7501	0.7568	0.7663	0.6836	0.6891
test_joint	CAWS-ETC	108,000	0.0071	0.9079	0.9354	0.9586	0.8087	0.8773	0.9651	0.0349	0.6833	0.9065	0.9114	0.9344	0.9379	0.8749	0.8811
test_temporal	Hard-rule union	108,000	1.0000	1.0000	1.0000	1.0000	1.0000	1.0000	1.0000	0.0000	1.0000	1.0000	1.0000	1.0000	1.0000	1.0000	1.0000

Table S2B. Rule-aligned metrics by role, method, and intervention mechanism.

Role	Method	Intervention type	Records	Threshold	AUROC	AUPRC	Precision	Recall	F1	Specificity	FPR	TPR@1%FPR
test_temporal	Hard-rule union	discount_total_mismatch	12,000	1.0000	1.0000	1.0000	1.0000	1.0000	1.0000	1.0000	0.0000	1.0000
test_temporal	Hard-rule union	entry_duration_outlier	12,000	1.0000	1.0000	1.0000	1.0000	1.0000	1.0000	1.0000	0.0000	1.0000
test_temporal	Hard-rule union	entry_time_invalid	12,000	1.0000	1.0000	1.0000	1.0000	1.0000	1.0000	1.0000	0.0000	1.0000
test_temporal	Hard-rule union	monetary_total_mismatch	12,000	1.0000	1.0000	1.0000	1.0000	1.0000	1.0000	1.0000	0.0000	1.0000

test_temporal	Hard-rule union	parser_corruption_stress	12,000	1.0000	1.0000	1.0000	1.0000	1.0000	1.0000	1.0000	0.0000	1.0000
test_temporal	Hard-rule union	previous_duration_outlier	12,000	1.0000	1.0000	1.0000	1.0000	1.0000	1.0000	1.0000	0.0000	1.0000
test_temporal	Hard-rule union	previous_time_conflict	12,000	1.0000	1.0000	1.0000	1.0000	1.0000	1.0000	1.0000	0.0000	1.0000
test_temporal	Hard-rule union	rare_missing_pattern	12,000	1.0000	1.0000	1.0000	1.0000	1.0000	1.0000	1.0000	0.0000	1.0000
test_temporal	Hard-rule union	semantic_rare_pair	12,000	1.0000	1.0000	1.0000	1.0000	1.0000	1.0000	1.0000	0.0000	1.0000
test_temporal	Anomaly-vote fraction	discount_total_mismatch	12,000	0.1000	1.0000	1.0000	1.0000	1.0000	1.0000	1.0000	0.0000	1.0000
test_temporal	Anomaly-vote fraction	entry_duration_outlier	12,000	0.1000	1.0000	1.0000	1.0000	1.0000	1.0000	1.0000	0.0000	1.0000
test_temporal	Anomaly-vote fraction	entry_time_invalid	12,000	0.1000	1.0000	1.0000	1.0000	1.0000	1.0000	1.0000	0.0000	1.0000
test_temporal	Anomaly-vote fraction	monetary_total_mismatch	12,000	0.1000	1.0000	1.0000	1.0000	1.0000	1.0000	1.0000	0.0000	1.0000
test_temporal	Anomaly-vote fraction	parser_corruption_stress	12,000	0.1000	1.0000	1.0000	1.0000	1.0000	1.0000	1.0000	0.0000	1.0000
test_temporal	Anomaly-vote fraction	previous_duration_outlier	12,000	0.1000	1.0000	1.0000	1.0000	1.0000	1.0000	1.0000	0.0000	1.0000
test_temporal	Anomaly-vote fraction	previous_time_conflict	12,000	0.1000	1.0000	1.0000	1.0000	1.0000	1.0000	1.0000	0.0000	1.0000
test_temporal	Anomaly-vote fraction	rare_missing_pattern	12,000	0.1000	1.0000	1.0000	1.0000	1.0000	1.0000	1.0000	0.0000	1.0000
test_temporal	Anomaly-vote fraction	semantic_rare_pair	12,000	0.1000	1.0000	1.0000	1.0000	1.0000	1.0000	1.0000	0.0000	1.0000
test_temporal	Probabilistic label model	discount_total_mismatch	12,000	0.5686	1.0000	1.0000	1.0000	1.0000	1.0000	1.0000	0.0000	1.0000
test_temporal	Probabilistic label model	entry_duration_outlier	12,000	0.5686	1.0000	1.0000	1.0000	1.0000	1.0000	1.0000	0.0000	1.0000
test_temporal	Probabilistic label model	entry_time_invalid	12,000	0.5686	1.0000	1.0000	1.0000	1.0000	1.0000	1.0000	0.0000	1.0000
test_temporal	Probabilistic label model	monetary_total_mismatch	12,000	0.5686	1.0000	1.0000	1.0000	1.0000	1.0000	1.0000	0.0000	1.0000
test_temporal	Probabilistic label model	parser_corruption_stress	12,000	0.5686	1.0000	1.0000	1.0000	1.0000	1.0000	1.0000	0.0000	1.0000
test_temporal	Probabilistic label model	previous_duration_outlier	12,000	0.5686	1.0000	1.0000	1.0000	1.0000	1.0000	1.0000	0.0000	1.0000
test_temporal	Probabilistic label model	previous_time_conflict	12,000	0.5686	1.0000	1.0000	1.0000	1.0000	1.0000	1.0000	0.0000	1.0000
test_temporal	Probabilistic label model	rare_missing_pattern	12,000	0.5686	1.0000	1.0000	1.0000	1.0000	1.0000	1.0000	0.0000	1.0000
test_temporal	Probabilistic label model	semantic_rare_pair	12,000	0.5686	1.0000	1.0000	1.0000	1.0000	1.0000	1.0000	0.0000	1.0000
test_temporal	Isolation Forest	discount_total_mismatch	12,000	0.5071	0.5989	0.5660	0.5419	0.8073	0.6485	0.3175	0.6825	0.0118
test_temporal	Isolation Forest	entry_duration_outlier	12,000	0.5071	0.6166	0.5835	0.5483	0.8340	0.6616	0.3130	0.6870	0.0138
test_temporal	Isolation Forest	entry_time_invalid	12,000	0.5071	0.9030	0.8485	0.5882	1.0000	0.7407	0.3000	0.7000	0.0845
test_temporal	Isolation Forest	monetary_total_mismatch	12,000	0.5071	0.5558	0.5340	0.5269	0.7692	0.6254	0.3095	0.6905	0.0103
test_temporal	Isolation Forest	parser_corruption_stress	12,000	0.5071	0.5000	0.5000	0.5000	0.6970	0.5823	0.3030	0.6970	0.0005
test_temporal	Isolation Forest	previous_duration_outlier	12,000	0.5071	0.6201	0.5817	0.5589	0.8567	0.6765	0.3240	0.6760	0.0157
test_temporal	Isolation Forest	previous_time_conflict	12,000	0.5071	0.9881	0.9751	0.5993	1.0000	0.7495	0.3315	0.6685	0.4380
test_temporal	Isolation Forest	rare_missing_pattern	12,000	0.5071	0.9832	0.9632	0.5945	1.0000	0.7457	0.3180	0.6820	0.2415

test_temporal	Isolation Forest	semantic_rare_pair	12,000	0.5071	0.9639	0.9369	0.5992	1.0000	0.7493	0.3310	0.6690	0.1830
test_temporal	CAWS-ETC	discount_total_mismatch	12,000	0.0071	0.7123	0.6848	0.9121	0.3062	0.4584	0.9705	0.0295	0.0205
test_temporal	CAWS-ETC	entry_duration_outlier	12,000	0.0071	1.0000	1.0000	0.9704	1.0000	0.9850	0.9695	0.0305	1.0000
test_temporal	CAWS-ETC	entry_time_invalid	12,000	0.0071	1.0000	1.0000	0.9657	1.0000	0.9826	0.9645	0.0355	1.0000
test_temporal	CAWS-ETC	monetary_total_mismatch	12,000	0.0071	1.0000	1.0000	0.9690	1.0000	0.9843	0.9680	0.0320	1.0000
test_temporal	CAWS-ETC	parser_corruption_stress	12,000	0.0071	0.5000	0.5000	0.5000	0.0250	0.0476	0.9750	0.0250	0.0095
test_temporal	CAWS-ETC	previous_duration_outlier	12,000	0.0071	1.0000	1.0000	0.9728	1.0000	0.9862	0.9720	0.0280	1.0000
test_temporal	CAWS-ETC	previous_time_conflict	12,000	0.0071	0.9777	0.9486	0.9683	0.9160	0.9414	0.9700	0.0300	0.1070
test_temporal	CAWS-ETC	rare_missing_pattern	12,000	0.0071	1.0000	1.0000	0.9737	1.0000	0.9867	0.9730	0.0270	1.0000
test_temporal	CAWS-ETC	semantic_rare_pair	12,000	0.0071	1.0000	1.0000	0.9690	1.0000	0.9843	0.9680	0.0320	1.0000
test_spatial	Hard-rule union	discount_total_mismatch	12,000	1.0000	1.0000	1.0000	1.0000	1.0000	1.0000	1.0000	0.0000	1.0000
test_spatial	Hard-rule union	entry_duration_outlier	12,000	1.0000	1.0000	1.0000	1.0000	1.0000	1.0000	1.0000	0.0000	1.0000
test_spatial	Hard-rule union	entry_time_invalid	12,000	1.0000	1.0000	1.0000	1.0000	1.0000	1.0000	1.0000	0.0000	1.0000
test_spatial	Hard-rule union	monetary_total_mismatch	12,000	1.0000	1.0000	1.0000	1.0000	1.0000	1.0000	1.0000	0.0000	1.0000
test_spatial	Hard-rule union	parser_corruption_stress	12,000	1.0000	1.0000	1.0000	1.0000	1.0000	1.0000	1.0000	0.0000	1.0000
test_spatial	Hard-rule union	previous_duration_outlier	12,000	1.0000	1.0000	1.0000	1.0000	1.0000	1.0000	1.0000	0.0000	1.0000
test_spatial	Hard-rule union	previous_time_conflict	12,000	1.0000	1.0000	1.0000	1.0000	1.0000	1.0000	1.0000	0.0000	1.0000
test_spatial	Hard-rule union	rare_missing_pattern	12,000	1.0000	1.0000	1.0000	1.0000	1.0000	1.0000	1.0000	0.0000	1.0000
test_spatial	Hard-rule union	semantic_rare_pair	12,000	1.0000	1.0000	1.0000	1.0000	1.0000	1.0000	1.0000	0.0000	1.0000
test_spatial	Anomaly-vote fraction	discount_total_mismatch	12,000	0.1000	1.0000	1.0000	1.0000	1.0000	1.0000	1.0000	0.0000	1.0000
test_spatial	Anomaly-vote fraction	entry_duration_outlier	12,000	0.1000	1.0000	1.0000	1.0000	1.0000	1.0000	1.0000	0.0000	1.0000
test_spatial	Anomaly-vote fraction	entry_time_invalid	12,000	0.1000	1.0000	1.0000	1.0000	1.0000	1.0000	1.0000	0.0000	1.0000
test_spatial	Anomaly-vote fraction	monetary_total_mismatch	12,000	0.1000	1.0000	1.0000	1.0000	1.0000	1.0000	1.0000	0.0000	1.0000
test_spatial	Anomaly-vote fraction	parser_corruption_stress	12,000	0.1000	1.0000	1.0000	1.0000	1.0000	1.0000	1.0000	0.0000	1.0000
test_spatial	Anomaly-vote fraction	previous_duration_outlier	12,000	0.1000	1.0000	1.0000	1.0000	1.0000	1.0000	1.0000	0.0000	1.0000
test_spatial	Anomaly-vote fraction	previous_time_conflict	12,000	0.1000	1.0000	1.0000	1.0000	1.0000	1.0000	1.0000	0.0000	1.0000
test_spatial	Anomaly-vote fraction	rare_missing_pattern	12,000	0.1000	1.0000	1.0000	1.0000	1.0000	1.0000	1.0000	0.0000	1.0000
test_spatial	Anomaly-vote fraction	semantic_rare_pair	12,000	0.1000	1.0000	1.0000	1.0000	1.0000	1.0000	1.0000	0.0000	1.0000
test_spatial	Probabilistic label model	discount_total_mismatch	12,000	0.5686	1.0000	1.0000	1.0000	1.0000	1.0000	1.0000	0.0000	1.0000
test_spatial	Probabilistic label model	entry_duration_outlier	12,000	0.5686	1.0000	1.0000	1.0000	1.0000	1.0000	1.0000	0.0000	1.0000
test_spatial	Probabilistic label model	entry_time_invalid	12,000	0.5686	1.0000	1.0000	1.0000	1.0000	1.0000	1.0000	0.0000	1.0000

test_spatial	Probabilistic label model	monetary_total_mismatch	12,000	0.5686	1.0000	1.0000	1.0000	1.0000	1.0000	1.0000	0.0000	1.0000
test_spatial	Probabilistic label model	parser_corruption_stress	12,000	0.5686	1.0000	1.0000	1.0000	1.0000	1.0000	1.0000	0.0000	1.0000
test_spatial	Probabilistic label model	previous_duration_outlier	12,000	0.5686	1.0000	1.0000	1.0000	1.0000	1.0000	1.0000	0.0000	1.0000
test_spatial	Probabilistic label model	previous_time_conflict	12,000	0.5686	1.0000	1.0000	1.0000	1.0000	1.0000	1.0000	0.0000	1.0000
test_spatial	Probabilistic label model	rare_missing_pattern	12,000	0.5686	1.0000	1.0000	1.0000	1.0000	1.0000	1.0000	0.0000	1.0000
test_spatial	Probabilistic label model	semantic_rare_pair	12,000	0.5686	1.0000	1.0000	1.0000	1.0000	1.0000	1.0000	0.0000	1.0000
test_spatial	Isolation Forest	discount_total_mismatch	12,000	0.5071	0.5882	0.5589	0.5681	0.4815	0.5212	0.6340	0.3660	0.0128
test_spatial	Isolation Forest	entry_duration_outlier	12,000	0.5071	0.5917	0.5705	0.5755	0.5092	0.5403	0.6245	0.3755	0.0143
test_spatial	Isolation Forest	entry_time_invalid	12,000	0.5071	0.9233	0.8687	0.7265	1.0000	0.8416	0.6235	0.3765	0.0880
test_spatial	Isolation Forest	monetary_total_mismatch	12,000	0.5071	0.5415	0.5274	0.5349	0.4417	0.4838	0.6160	0.3840	0.0108
test_spatial	Isolation Forest	parser_corruption_stress	12,000	0.5071	0.5000	0.5000	0.5000	0.3930	0.4401	0.6070	0.3930	0.0005
test_spatial	Isolation Forest	previous_duration_outlier	12,000	0.5071	0.6031	0.5682	0.5724	0.4585	0.5092	0.6575	0.3425	0.0162
test_spatial	Isolation Forest	previous_time_conflict	12,000	0.5071	0.9910	0.9809	0.7267	1.0000	0.8418	0.6240	0.3760	0.6710
test_spatial	Isolation Forest	rare_missing_pattern	12,000	0.5071	0.9838	0.9619	0.7286	1.0000	0.8430	0.6275	0.3725	0.2675
test_spatial	Isolation Forest	semantic_rare_pair	12,000	0.5071	0.9774	0.9528	0.7302	1.0000	0.8441	0.6305	0.3695	0.2372
test_spatial	CAWS-ETC	discount_total_mismatch	12,000	0.0071	0.7682	0.6952	0.7146	0.0513	0.0958	0.9795	0.0205	0.0125
test_spatial	CAWS-ETC	entry_duration_outlier	12,000	0.0071	1.0000	1.0000	0.9843	1.0000	0.9921	0.9840	0.0160	1.0000
test_spatial	CAWS-ETC	entry_time_invalid	12,000	0.0071	1.0000	1.0000	0.9843	1.0000	0.9921	0.9840	0.0160	1.0000
test_spatial	CAWS-ETC	monetary_total_mismatch	12,000	0.0071	1.0000	1.0000	0.9833	1.0000	0.9916	0.9830	0.0170	1.0000
test_spatial	CAWS-ETC	parser_corruption_stress	12,000	0.0071	0.5000	0.5000	0.5000	0.0135	0.0263	0.9865	0.0135	0.0005
test_spatial	CAWS-ETC	previous_duration_outlier	12,000	0.0071	1.0000	1.0000	0.9847	1.0000	0.9923	0.9845	0.0155	1.0000
test_spatial	CAWS-ETC	previous_time_conflict	12,000	0.0071	0.9888	0.9716	0.9812	0.8340	0.9016	0.9840	0.0160	0.6380
test_spatial	CAWS-ETC	rare_missing_pattern	12,000	0.0071	1.0000	1.0000	0.9833	1.0000	0.9916	0.9830	0.0170	1.0000
test_spatial	CAWS-ETC	semantic_rare_pair	12,000	0.0071	1.0000	1.0000	0.9886	1.0000	0.9943	0.9885	0.0115	1.0000
test_joint	Hard-rule union	discount_total_mismatch	12,000	1.0000	1.0000	1.0000	1.0000	1.0000	1.0000	1.0000	0.0000	1.0000
test_joint	Hard-rule union	entry_duration_outlier	12,000	1.0000	1.0000	1.0000	1.0000	1.0000	1.0000	1.0000	0.0000	1.0000
test_joint	Hard-rule union	entry_time_invalid	12,000	1.0000	1.0000	1.0000	1.0000	1.0000	1.0000	1.0000	0.0000	1.0000
test_joint	Hard-rule union	monetary_total_mismatch	12,000	1.0000	1.0000	1.0000	1.0000	1.0000	1.0000	1.0000	0.0000	1.0000
test_joint	Hard-rule union	parser_corruption_stress	12,000	1.0000	1.0000	1.0000	1.0000	1.0000	1.0000	1.0000	0.0000	1.0000
test_joint	Hard-rule union	previous_duration_outlier	12,000	1.0000	1.0000	1.0000	1.0000	1.0000	1.0000	1.0000	0.0000	1.0000
test_joint	Hard-rule union	previous_time_conflict	12,000	1.0000	1.0000	1.0000	1.0000	1.0000	1.0000	1.0000	0.0000	1.0000

test_joint	Hard-rule union	rare_missing_pattern	12,000	1.0000	1.0000	1.0000	1.0000	1.0000	1.0000	1.0000	0.0000	1.0000
test_joint	Hard-rule union	semantic_rare_pair	12,000	1.0000	1.0000	1.0000	1.0000	1.0000	1.0000	1.0000	0.0000	1.0000
test_joint	Anomaly-vote fraction	discount_total_mismatch	12,000	0.1000	1.0000	1.0000	1.0000	1.0000	1.0000	1.0000	0.0000	1.0000
test_joint	Anomaly-vote fraction	entry_duration_outlier	12,000	0.1000	1.0000	1.0000	1.0000	1.0000	1.0000	1.0000	0.0000	1.0000
test_joint	Anomaly-vote fraction	entry_time_invalid	12,000	0.1000	1.0000	1.0000	1.0000	1.0000	1.0000	1.0000	0.0000	1.0000
test_joint	Anomaly-vote fraction	monetary_total_mismatch	12,000	0.1000	1.0000	1.0000	1.0000	1.0000	1.0000	1.0000	0.0000	1.0000
test_joint	Anomaly-vote fraction	parser_corruption_stress	12,000	0.1000	1.0000	1.0000	1.0000	1.0000	1.0000	1.0000	0.0000	1.0000
test_joint	Anomaly-vote fraction	previous_duration_outlier	12,000	0.1000	1.0000	1.0000	1.0000	1.0000	1.0000	1.0000	0.0000	1.0000
test_joint	Anomaly-vote fraction	previous_time_conflict	12,000	0.1000	1.0000	1.0000	1.0000	1.0000	1.0000	1.0000	0.0000	1.0000
test_joint	Anomaly-vote fraction	rare_missing_pattern	12,000	0.1000	1.0000	1.0000	1.0000	1.0000	1.0000	1.0000	0.0000	1.0000
test_joint	Anomaly-vote fraction	semantic_rare_pair	12,000	0.1000	1.0000	1.0000	1.0000	1.0000	1.0000	1.0000	0.0000	1.0000
test_joint	Probabilistic label model	discount_total_mismatch	12,000	0.5686	1.0000	1.0000	1.0000	1.0000	1.0000	1.0000	0.0000	1.0000
test_joint	Probabilistic label model	entry_duration_outlier	12,000	0.5686	1.0000	1.0000	1.0000	1.0000	1.0000	1.0000	0.0000	1.0000
test_joint	Probabilistic label model	entry_time_invalid	12,000	0.5686	1.0000	1.0000	1.0000	1.0000	1.0000	1.0000	0.0000	1.0000
test_joint	Probabilistic label model	monetary_total_mismatch	12,000	0.5686	1.0000	1.0000	1.0000	1.0000	1.0000	1.0000	0.0000	1.0000
test_joint	Probabilistic label model	parser_corruption_stress	12,000	0.5686	1.0000	1.0000	1.0000	1.0000	1.0000	1.0000	0.0000	1.0000
test_joint	Probabilistic label model	previous_duration_outlier	12,000	0.5686	1.0000	1.0000	1.0000	1.0000	1.0000	1.0000	0.0000	1.0000
test_joint	Probabilistic label model	previous_time_conflict	12,000	0.5686	1.0000	1.0000	1.0000	1.0000	1.0000	1.0000	0.0000	1.0000
test_joint	Probabilistic label model	rare_missing_pattern	12,000	0.5686	1.0000	1.0000	1.0000	1.0000	1.0000	1.0000	0.0000	1.0000
test_joint	Probabilistic label model	semantic_rare_pair	12,000	0.5686	1.0000	1.0000	1.0000	1.0000	1.0000	1.0000	0.0000	1.0000
test_joint	Isolation Forest	discount_total_mismatch	12,000	0.5071	0.6074	0.5735	0.5458	0.8008	0.6491	0.3335	0.6665	0.0117
test_joint	Isolation Forest	entry_duration_outlier	12,000	0.5071	0.6213	0.5855	0.5564	0.8117	0.6602	0.3530	0.6470	0.0197
test_joint	Isolation Forest	entry_time_invalid	12,000	0.5071	0.9096	0.8417	0.5947	1.0000	0.7459	0.3185	0.6815	0.0600
test_joint	Isolation Forest	monetary_total_mismatch	12,000	0.5071	0.5597	0.5377	0.5289	0.7408	0.6171	0.3400	0.6600	0.0123
test_joint	Isolation Forest	parser_corruption_stress	12,000	0.5071	0.5000	0.5000	0.5000	0.6620	0.5697	0.3380	0.6620	0.0035
test_joint	Isolation Forest	previous_duration_outlier	12,000	0.5071	0.6221	0.5798	0.5589	0.8427	0.6721	0.3350	0.6650	0.0125
test_joint	Isolation Forest	previous_time_conflict	12,000	0.5071	0.9844	0.9581	0.5977	1.0000	0.7482	0.3270	0.6730	0.3175
test_joint	Isolation Forest	rare_missing_pattern	12,000	0.5071	0.9833	0.9534	0.6044	1.0000	0.7534	0.3455	0.6545	0.1840
test_joint	Isolation Forest	semantic_rare_pair	12,000	0.5071	0.9606	0.9178	0.6001	1.0000	0.7500	0.3335	0.6665	0.0692
test_joint	CAWS-ETC	discount_total_mismatch	12,000	0.0071	0.6997	0.6695	0.8897	0.3107	0.4605	0.9615	0.0385	0.0223
test_joint	CAWS-ETC	entry_duration_outlier	12,000	0.0071	1.0000	1.0000	0.9732	1.0000	0.9864	0.9725	0.0275	1.0000

test_joint	CAWS-ETC	entry_time_invalid	12,000	0.0071	1.0000	1.0000	0.9695	1.0000	0.9845	0.9685	0.0315	1.0000
test_joint	CAWS-ETC	monetary_total_mismatch	12,000	0.0071	1.0000	1.0000	0.9583	1.0000	0.9787	0.9565	0.0435	1.0000
test_joint	CAWS-ETC	parser_corruption_stress	12,000	0.0071	0.5000	0.5000	0.5000	0.0340	0.0637	0.9660	0.0340	0.0005
test_joint	CAWS-ETC	previous_duration_outlier	12,000	0.0071	1.0000	1.0000	0.9657	1.0000	0.9826	0.9645	0.0355	1.0000
test_joint	CAWS-ETC	previous_time_conflict	12,000	0.0071	0.9777	0.9469	0.9674	0.9335	0.9501	0.9685	0.0315	0.1250
test_joint	CAWS-ETC	rare_missing_pattern	12,000	0.0071	1.0000	1.0000	0.9648	1.0000	0.9821	0.9635	0.0365	1.0000
test_joint	CAWS-ETC	semantic_rare_pair	12,000	0.0071	1.0000	1.0000	0.9657	1.0000	0.9826	0.9645	0.0355	1.0000

Table S2C. Rule-aligned metrics by role, method, and severity.

Role	Method	Severity	Records	Threshold	AUROC	AUPRC	Precision	Recall	F1	Specificity	FPR	TPR@1%FPR
test_temporal	Hard-rule union	mild	36,000	1.0000	1.0000	1.0000	1.0000	1.0000	1.0000	1.0000	0.0000	1.0000
test_temporal	Hard-rule union	moderate	36,000	1.0000	1.0000	1.0000	1.0000	1.0000	1.0000	1.0000	0.0000	1.0000
test_temporal	Hard-rule union	severe	36,000	1.0000	1.0000	1.0000	1.0000	1.0000	1.0000	1.0000	0.0000	1.0000
test_temporal	Anomaly-vote fraction	mild	36,000	0.1000	1.0000	1.0000	1.0000	1.0000	1.0000	1.0000	0.0000	1.0000
test_temporal	Anomaly-vote fraction	moderate	36,000	0.1000	1.0000	1.0000	1.0000	1.0000	1.0000	1.0000	0.0000	1.0000
test_temporal	Anomaly-vote fraction	severe	36,000	0.1000	1.0000	1.0000	1.0000	1.0000	1.0000	1.0000	0.0000	1.0000
test_temporal	Probabilistic label model	mild	36,000	0.5686	1.0000	1.0000	1.0000	1.0000	1.0000	1.0000	0.0000	1.0000
test_temporal	Probabilistic label model	moderate	36,000	0.5686	1.0000	1.0000	1.0000	1.0000	1.0000	1.0000	0.0000	1.0000
test_temporal	Probabilistic label model	severe	36,000	0.5686	1.0000	1.0000	1.0000	1.0000	1.0000	1.0000	0.0000	1.0000
test_temporal	Isolation Forest	mild	36,000	0.5071	0.7226	0.7517	0.5566	0.8580	0.6752	0.3164	0.6836	0.1129
test_temporal	Isolation Forest	moderate	36,000	0.5071	0.7414	0.7664	0.5624	0.8787	0.6859	0.3164	0.6836	0.1177
test_temporal	Isolation Forest	severe	36,000	0.5071	0.7828	0.7962	0.5732	0.9180	0.7057	0.3164	0.6836	0.1387
test_temporal	CAWS-ETC	mild	36,000	0.0071	0.9088	0.9360	0.9641	0.8052	0.8775	0.9701	0.0299	0.6815
test_temporal	CAWS-ETC	moderate	36,000	0.0071	0.9089	0.9361	0.9641	0.8052	0.8775	0.9701	0.0299	0.6815
test_temporal	CAWS-ETC	severe	36,000	0.0071	0.9093	0.9363	0.9641	0.8053	0.8776	0.9701	0.0299	0.6815
test_spatial	Hard-rule union	mild	36,000	1.0000	1.0000	1.0000	1.0000	1.0000	1.0000	1.0000	0.0000	1.0000
test_spatial	Hard-rule union	moderate	36,000	1.0000	1.0000	1.0000	1.0000	1.0000	1.0000	1.0000	0.0000	1.0000
test_spatial	Hard-rule union	severe	36,000	1.0000	1.0000	1.0000	1.0000	1.0000	1.0000	1.0000	0.0000	1.0000
test_spatial	Anomaly-vote fraction	mild	36,000	0.1000	1.0000	1.0000	1.0000	1.0000	1.0000	1.0000	0.0000	1.0000
test_spatial	Anomaly-vote fraction	moderate	36,000	0.1000	1.0000	1.0000	1.0000	1.0000	1.0000	1.0000	0.0000	1.0000
test_spatial	Anomaly-vote fraction	severe	36,000	0.1000	1.0000	1.0000	1.0000	1.0000	1.0000	1.0000	0.0000	1.0000
test_spatial	Probabilistic label model	mild	36,000	0.5686	1.0000	1.0000	1.0000	1.0000	1.0000	1.0000	0.0000	1.0000
test_spatial	Probabilistic label model	moderate	36,000	0.5686	1.0000	1.0000	1.0000	1.0000	1.0000	1.0000	0.0000	1.0000

test_spatial	Probabilistic label model	severe	36,000	0.5686	1.0000	1.0000	1.0000	1.0000	1.0000	1.0000	0.0000	1.0000
test_spatial	Isolation Forest	mild	36,000	0.5071	0.7234	0.7535	0.6434	0.6728	0.6578	0.6272	0.3728	0.1048
test_spatial	Isolation Forest	moderate	36,000	0.5071	0.7371	0.7642	0.6496	0.6912	0.6698	0.6272	0.3728	0.1153
test_spatial	Isolation Forest	severe	36,000	0.5071	0.7709	0.7874	0.6621	0.7306	0.6946	0.6272	0.3728	0.1452
test_spatial	CAWS-ETC	mild	36,000	0.0071	0.9157	0.9403	0.9797	0.7663	0.8600	0.9841	0.0159	0.7078
test_spatial	CAWS-ETC	moderate	36,000	0.0071	0.9158	0.9404	0.9797	0.7666	0.8602	0.9841	0.0159	0.7078
test_spatial	CAWS-ETC	severe	36,000	0.0071	0.9160	0.9406	0.9797	0.7667	0.8602	0.9841	0.0159	0.7078
test_joint	Hard-rule union	mild	36,000	1.0000	1.0000	1.0000	1.0000	1.0000	1.0000	1.0000	0.0000	1.0000
test_joint	Hard-rule union	moderate	36,000	1.0000	1.0000	1.0000	1.0000	1.0000	1.0000	1.0000	0.0000	1.0000
test_joint	Hard-rule union	severe	36,000	1.0000	1.0000	1.0000	1.0000	1.0000	1.0000	1.0000	0.0000	1.0000
test_joint	Anomaly-vote fraction	mild	36,000	0.1000	1.0000	1.0000	1.0000	1.0000	1.0000	1.0000	0.0000	1.0000
test_joint	Anomaly-vote fraction	moderate	36,000	0.1000	1.0000	1.0000	1.0000	1.0000	1.0000	1.0000	0.0000	1.0000
test_joint	Anomaly-vote fraction	severe	36,000	0.1000	1.0000	1.0000	1.0000	1.0000	1.0000	1.0000	0.0000	1.0000
test_joint	Probabilistic label model	mild	36,000	0.5686	1.0000	1.0000	1.0000	1.0000	1.0000	1.0000	0.0000	1.0000
test_joint	Probabilistic label model	moderate	36,000	0.5686	1.0000	1.0000	1.0000	1.0000	1.0000	1.0000	0.0000	1.0000
test_joint	Probabilistic label model	severe	36,000	0.5686	1.0000	1.0000	1.0000	1.0000	1.0000	1.0000	0.0000	1.0000
test_joint	Isolation Forest	mild	36,000	0.5071	0.7205	0.7411	0.5601	0.8455	0.6738	0.3360	0.6640	0.0834
test_joint	Isolation Forest	moderate	36,000	0.5071	0.7397	0.7554	0.5661	0.8664	0.6848	0.3360	0.6640	0.0888
test_joint	Isolation Forest	severe	36,000	0.5071	0.7825	0.7854	0.5774	0.9074	0.7058	0.3360	0.6640	0.1042
test_joint	CAWS-ETC	mild	36,000	0.0071	0.9076	0.9353	0.9586	0.8087	0.8773	0.9651	0.0349	0.6833
test_joint	CAWS-ETC	moderate	36,000	0.0071	0.9078	0.9354	0.9586	0.8087	0.8773	0.9651	0.0349	0.6833
test_joint	CAWS-ETC	severe	36,000	0.0071	0.9082	0.9356	0.9586	0.8087	0.8773	0.9651	0.0349	0.6833

Table S2D. Low-prevalence stress tests in the joint-transfer subset.

Method	Synthetic prevalence	Records	Threshold	AUROC	AUPRC	Precision	Recall	F1	Specificity	FPR	TPR@1%FPR
Hard-rule union	0.0100	100,000	1.0000	1.0000	1.0000	1.0000	1.0000	1.0000	1.0000	0.0000	1.0000
Anomaly-vote fraction	0.0100	100,000	0.1000	1.0000	1.0000	1.0000	1.0000	1.0000	1.0000	0.0000	1.0000
Probabilistic label model	0.0100	100,000	0.5686	1.0000	1.0000	1.0000	1.0000	1.0000	1.0000	0.0000	1.0000
Isolation Forest	0.0100	100,000	0.5071	0.7302	0.0486	0.0128	0.8550	0.0252	0.3340	0.6660	0.0830
CAWS-ETC	0.0100	100,000	0.0071	0.8990	0.6945	0.1864	0.7880	0.3015	0.9653	0.0347	0.6650
Hard-rule union	0.0300	100,000	1.0000	1.0000	1.0000	1.0000	1.0000	1.0000	1.0000	0.0000	1.0000
Anomaly-vote fraction	0.0300	100,000	0.1000	1.0000	1.0000	1.0000	1.0000	1.0000	1.0000	0.0000	1.0000
Probabilistic label model	0.0300	100,000	0.5686	1.0000	1.0000	1.0000	1.0000	1.0000	1.0000	0.0000	1.0000

Isolation Forest	0.0300	100,000	0.5071	0.7453	0.1317	0.0389	0.8693	0.0744	0.3353	0.6647	0.0960
CAWS-ETC	0.0300	100,000	0.0071	0.9065	0.7565	0.4159	0.8097	0.5495	0.9648	0.0352	0.6803
Hard-rule union	0.0500	100,000	1.0000	1.0000	1.0000	1.0000	1.0000	1.0000	1.0000	0.0000	1.0000
Anomaly-vote fraction	0.0500	100,000	0.1000	1.0000	1.0000	1.0000	1.0000	1.0000	1.0000	0.0000	1.0000
Probabilistic label model	0.0500	100,000	0.5686	1.0000	1.0000	1.0000	1.0000	1.0000	1.0000	0.0000	1.0000
Isolation Forest	0.0500	100,000	0.5071	0.7440	0.1934	0.0646	0.8710	0.1202	0.3357	0.6643	0.0860
CAWS-ETC	0.0500	100,000	0.0071	0.9092	0.7860	0.5523	0.8148	0.6583	0.9652	0.0348	0.6824

Table S2E. Validation-selected thresholds and validation metrics.

Method	Score column	Selected threshold	AUROC	AUPRC	Precision	Recall	F1	Specificity	FPR	TPR@1%FPR
Hard-rule union	score_hard_rule	1.0000	1.0000	1.0000	1.0000	1.0000	1.0000	1.0000	0.0000	1.0000
Anomaly-vote fraction	score_vote_fraction	0.1000	1.0000	1.0000	1.0000	1.0000	1.0000	1.0000	0.0000	1.0000
Probabilistic label model	score_label_model	0.5686	1.0000	1.0000	1.0000	1.0000	1.0000	1.0000	0.0000	1.0000
Isolation Forest	score_isolation_forest	0.5071	0.7452	0.7753	0.6127	0.7850	0.6883	0.5039	0.4961	0.1894
CAWS-ETC	score_caws_etc	0.0071	0.9113	0.9373	0.9755	0.7958	0.8765	0.9800	0.0200	0.7267

Note: For the parser-corruption stress condition, perfect hard-rule and vote-fraction recovery results from explicit activation of the retained deterministic parser safeguard. The probabilistic label model excluded the zero-coverage parser anomaly source; its separation of the paired records is attributable to loss of the joint-normal anchor. These results therefore represent rule/anchor recovery rather than learned parser-error generalization.

Table S3A. Retrained variant configurations and internal weak-label diagnostics.

Variant	Variant family	Features	Training rows	Validation rows	Anomaly threshold	Normal threshold	Random seed	Source balanced	Best iteration	Weak-label AUROC	Weak-label AUPRC
full_retrained	reference	46	600,000	150,000	0.9000	0.1000	20,221,213	False	26	1.0000	1.0000
without_monetary	ablation	36	600,000	150,000	0.9000	0.1000	20,221,213	False	69	0.9939	0.9771
without_temporal	ablation	37	600,000	150,000	0.9000	0.1000	20,221,213	False	614	0.8151	0.6477
without_structural	ablation	35	600,000	150,000	0.9000	0.1000	20,221,213	False	122	1.0000	1.0000
without_semantic_context	ablation	30	600,000	150,000	0.9000	0.1000	20,221,213	False	349	1.0000	1.0000
source_balanced	sampling	46	600,000	150,000	0.9000	0.1000	20,221,213	True	6	1.0000	1.0000
train_rows_100000	training_size	46	100,000	150,000	0.9000	0.1000	20,221,213	False	42	1.0000	1.0000
train_rows_300000	training_size	46	300,000	150,000	0.9000	0.1000	20,221,213	False	4	1.0000	1.0000
confidence_0.85_0.15	confidence_threshold	46	300,000	150,000	0.8500	0.1500	20,221,213	False	4	1.0000	1.0000
confidence_0.95_0.05	confidence_threshold	46	300,000	150,000	0.9500	0.0500	20,221,213	False	4	1.0000	1.0000
seed_20221214	random_seed	46	300,000	150,000	0.9000	0.1000	20,221,214	False	1	1.0000	1.0000

seed_20221215	random_seed	46	300,000	150,000	0.9000	0.1000	20,221,215	False	6	1.0000	1.0000

Table S3B. Full variant performance on the rule-orthogonal challenge.

Variant	Variant family	Role	Threshold	Records	AUROC	AUPRC	Precision	Recall	F1
full_retrained	reference	test_temporal	0.3891	54,000	0.8846	0.9208	0.9888	0.7619	0.8606
full_retrained	reference	test_spatial	0.3891	54,000	0.8886	0.9247	0.9936	0.7396	0.8480
full_retrained	reference	test_joint	0.3891	54,000	0.8813	0.9185	0.9859	0.7611	0.8590
without_monetary	ablation	test_temporal	0.3279	54,000	0.8668	0.9132	0.9956	0.7509	0.8561
without_monetary	ablation	test_spatial	0.3279	54,000	0.8645	0.9113	0.9921	0.7432	0.8498
without_monetary	ablation	test_joint	0.3279	54,000	0.8664	0.9123	0.9925	0.7477	0.8529
without_temporal	ablation	test_temporal	0.3455	54,000	0.7462	0.8093	0.5994	0.7809	0.6782
without_temporal	ablation	test_spatial	0.3455	54,000	0.7399	0.8045	0.6310	0.7206	0.6728
without_temporal	ablation	test_joint	0.3455	54,000	0.7432	0.8076	0.5864	0.7920	0.6739
without_structural	ablation	test_temporal	0.3213	54,000	0.8908	0.9293	1.0000	0.7774	0.8747
without_structural	ablation	test_spatial	0.3213	54,000	0.8881	0.9281	0.9999	0.7774	0.8747
without_structural	ablation	test_joint	0.3213	54,000	0.8943	0.9308	0.9994	0.7773	0.8745
without_semantic_context	ablation	test_temporal	0.0008	54,000	0.8724	0.9085	0.9701	0.6922	0.8079
without_semantic_context	ablation	test_spatial	0.0008	54,000	0.8862	0.9173	0.9776	0.6936	0.8115
without_semantic_context	ablation	test_joint	0.0008	54,000	0.8736	0.9095	0.9759	0.6872	0.8065
source_balanced	sampling	test_temporal	0.4814	54,000	0.8875	0.9161	0.9999	0.7773	0.8747
source_balanced	sampling	test_spatial	0.4814	54,000	0.8887	0.9234	0.9999	0.7776	0.8749
source_balanced	sampling	test_joint	0.4814	54,000	0.8874	0.9161	0.9996	0.7772	0.8745
train_rows_100000	training_size	test_temporal	0.2586	54,000	0.8433	0.8905	0.9980	0.6670	0.7996
train_rows_100000	training_size	test_spatial	0.2586	54,000	0.8423	0.8915	0.9972	0.6676	0.7997
train_rows_100000	training_size	test_joint	0.2586	54,000	0.8439	0.8910	0.9973	0.6672	0.7995
train_rows_300000	training_size	test_temporal	0.4968	54,000	0.8320	0.8668	0.9972	0.6672	0.7995
train_rows_300000	training_size	test_spatial	0.4968	54,000	0.8327	0.8802	0.9987	0.6667	0.7996
train_rows_300000	training_size	test_joint	0.4968	54,000	0.8304	0.8656	0.9965	0.6677	0.7997
confidence_0.85_0.15	confidence_threshold	test_temporal	0.4968	54,000	0.8320	0.8668	0.9972	0.6672	0.7995
confidence_0.85_0.15	confidence_threshold	test_spatial	0.4968	54,000	0.8327	0.8802	0.9987	0.6667	0.7996
confidence_0.85_0.15	confidence_threshold	test_joint	0.4968	54,000	0.8304	0.8656	0.9965	0.6677	0.7997

confidence_0.95_0.05	confidence_threshold	test_temporal	0.4968	54,000	0.8320	0.8668	0.9972	0.6672	0.7995
confidence_0.95_0.05	confidence_threshold	test_spatial	0.4968	54,000	0.8327	0.8802	0.9987	0.6667	0.7996
confidence_0.95_0.05	confidence_threshold	test_joint	0.4968	54,000	0.8304	0.8656	0.9965	0.6677	0.7997
seed_20221214	random_seed	test_temporal	0.5150	54,000	0.8333	0.8333	1.0000	0.6667	0.8000
seed_20221214	random_seed	test_spatial	0.5150	54,000	0.8333	0.8333	1.0000	0.6667	0.8000
seed_20221214	random_seed	test_joint	0.5150	54,000	0.8333	0.8333	1.0000	0.6667	0.8000
seed_20221215	random_seed	test_temporal	0.4627	54,000	0.8315	0.8730	0.9884	0.6649	0.7950
seed_20221215	random_seed	test_spatial	0.4627	54,000	0.8324	0.8824	0.9907	0.6658	0.7964
seed_20221215	random_seed	test_joint	0.4627	54,000	0.8294	0.8719	0.9837	0.6647	0.7934

Table S3C. Full variant association with independent operational proxies.

Variant	Variant family	Role	Proxy	Records	AUROC	AUPRC
full_retrained	reference	test_temporal	trade_failure	200,000	0.9789	0.8385
full_retrained	reference	test_temporal	special_present	200,000	0.8370	0.7208
full_retrained	reference	test_temporal	fee_calc_nonzero	200,000	0.9842	0.8281
full_retrained	reference	test_spatial	trade_failure	200,000	0.9736	0.7782
full_retrained	reference	test_spatial	special_present	200,000	0.7729	0.6243
full_retrained	reference	test_spatial	fee_calc_nonzero	200,000	0.9776	0.7694
full_retrained	reference	test_joint	trade_failure	158,896	0.9733	0.7909
full_retrained	reference	test_joint	special_present	158,896	0.8150	0.6734
full_retrained	reference	test_joint	fee_calc_nonzero	158,896	0.9788	0.7858
without_monetary	ablation	test_temporal	trade_failure	200,000	0.8597	0.4913
without_monetary	ablation	test_temporal	special_present	200,000	0.7335	0.4520
without_monetary	ablation	test_temporal	fee_calc_nonzero	200,000	0.8637	0.4889
without_monetary	ablation	test_spatial	trade_failure	200,000	0.8802	0.4873
without_monetary	ablation	test_spatial	special_present	200,000	0.6728	0.4227
without_monetary	ablation	test_spatial	fee_calc_nonzero	200,000	0.8858	0.4871
without_monetary	ablation	test_joint	trade_failure	158,896	0.8473	0.4753
without_monetary	ablation	test_joint	special_present	158,896	0.7121	0.4453
without_monetary	ablation	test_joint	fee_calc_nonzero	158,896	0.8524	0.4761
without_temporal	ablation	test_temporal	trade_failure	200,000	0.9952	0.9860
without_temporal	ablation	test_temporal	special_present	200,000	0.8699	0.8435

without_temporal	ablation	test_temporal	fee_calc_nonzero	200,000	1.0000	1.0000
without_temporal	ablation	test_spatial	trade_failure	200,000	0.9956	0.9847
without_temporal	ablation	test_spatial	special_present	200,000	0.7943	0.7609
without_temporal	ablation	test_spatial	fee_calc_nonzero	200,000	1.0000	1.0000
without_temporal	ablation	test_joint	trade_failure	158,896	0.9955	0.9847
without_temporal	ablation	test_joint	special_present	158,896	0.8406	0.8122
without_temporal	ablation	test_joint	fee_calc_nonzero	158,896	1.0000	1.0000
without_structural	ablation	test_temporal	trade_failure	200,000	0.9818	0.8594
without_structural	ablation	test_temporal	special_present	200,000	0.8295	0.7340
without_structural	ablation	test_temporal	fee_calc_nonzero	200,000	0.9841	0.8483
without_structural	ablation	test_spatial	trade_failure	200,000	0.9760	0.7886
without_structural	ablation	test_spatial	special_present	200,000	0.7602	0.6234
without_structural	ablation	test_spatial	fee_calc_nonzero	200,000	0.9785	0.7817
without_structural	ablation	test_joint	trade_failure	158,896	0.9755	0.8094
without_structural	ablation	test_joint	special_present	158,896	0.8066	0.6846
without_structural	ablation	test_joint	fee_calc_nonzero	158,896	0.9783	0.8043
without_semantic_context	ablation	test_temporal	trade_failure	200,000	0.9426	0.7060
without_semantic_context	ablation	test_temporal	special_present	200,000	0.8373	0.6306
without_semantic_context	ablation	test_temporal	fee_calc_nonzero	200,000	0.9464	0.7036
without_semantic_context	ablation	test_spatial	trade_failure	200,000	0.9608	0.7556
without_semantic_context	ablation	test_spatial	special_present	200,000	0.8082	0.6265
without_semantic_context	ablation	test_spatial	fee_calc_nonzero	200,000	0.9643	0.7571
without_semantic_context	ablation	test_joint	trade_failure	158,896	0.9374	0.6861
without_semantic_context	ablation	test_joint	special_present	158,896	0.8219	0.6162
without_semantic_context	ablation	test_joint	fee_calc_nonzero	158,896	0.9411	0.6863
source_balanced	sampling	test_temporal	trade_failure	200,000	0.9671	0.8290
source_balanced	sampling	test_temporal	special_present	200,000	0.8489	0.7176
source_balanced	sampling	test_temporal	fee_calc_nonzero	200,000	0.9747	0.8178
source_balanced	sampling	test_spatial	trade_failure	200,000	0.9654	0.7773
source_balanced	sampling	test_spatial	special_present	200,000	0.7923	0.6296
source_balanced	sampling	test_spatial	fee_calc_nonzero	200,000	0.9696	0.7636

source_balanced	sampling	test_joint	trade_failure	158,896	0.9633	0.8065
source_balanced	sampling	test_joint	special_present	158,896	0.8261	0.6831
source_balanced	sampling	test_joint	fee_calc_nonzero	158,896	0.9710	0.8004
train_rows_100000	training_size	test_temporal	trade_failure	200,000	0.9708	0.8310
train_rows_100000	training_size	test_temporal	special_present	200,000	0.8348	0.7208
train_rows_100000	training_size	test_temporal	fee_calc_nonzero	200,000	0.9730	0.8088
train_rows_100000	training_size	test_spatial	trade_failure	200,000	0.9721	0.8029
train_rows_100000	training_size	test_spatial	special_present	200,000	0.7815	0.6453
train_rows_100000	training_size	test_spatial	fee_calc_nonzero	200,000	0.9733	0.7820
train_rows_100000	training_size	test_joint	trade_failure	158,896	0.9656	0.7880
train_rows_100000	training_size	test_joint	special_present	158,896	0.8098	0.6770
train_rows_100000	training_size	test_joint	fee_calc_nonzero	158,896	0.9683	0.7735
train_rows_300000	training_size	test_temporal	trade_failure	200,000	0.9704	0.8401
train_rows_300000	training_size	test_temporal	special_present	200,000	0.8457	0.7200
train_rows_300000	training_size	test_temporal	fee_calc_nonzero	200,000	0.9753	0.8273
train_rows_300000	training_size	test_spatial	trade_failure	200,000	0.9676	0.7928
train_rows_300000	training_size	test_spatial	special_present	200,000	0.7921	0.6370
train_rows_300000	training_size	test_spatial	fee_calc_nonzero	200,000	0.9707	0.7792
train_rows_300000	training_size	test_joint	trade_failure	158,896	0.9664	0.8208
train_rows_300000	training_size	test_joint	special_present	158,896	0.8237	0.6867
train_rows_300000	training_size	test_joint	fee_calc_nonzero	158,896	0.9717	0.8139
confidence_0.85_0.15	confidence_threshold	test_temporal	trade_failure	200,000	0.9704	0.8401
confidence_0.85_0.15	confidence_threshold	test_temporal	special_present	200,000	0.8457	0.7200
confidence_0.85_0.15	confidence_threshold	test_temporal	fee_calc_nonzero	200,000	0.9753	0.8273
confidence_0.85_0.15	confidence_threshold	test_spatial	trade_failure	200,000	0.9676	0.7928
confidence_0.85_0.15	confidence_threshold	test_spatial	special_present	200,000	0.7921	0.6370
confidence_0.85_0.15	confidence_threshold	test_spatial	fee_calc_nonzero	200,000	0.9707	0.7792
confidence_0.85_0.15	confidence_threshold	test_joint	trade_failure	158,896	0.9664	0.8208
confidence_0.85_0.15	confidence_threshold	test_joint	special_present	158,896	0.8237	0.6867
confidence_0.85_0.15	confidence_threshold	test_joint	fee_calc_nonzero	158,896	0.9717	0.8139
confidence_0.95_0.05	confidence_threshold	test_temporal	trade_failure	200,000	0.9704	0.8401

confidence_0.95_0.05	confidence_threshold	test_temporal	special_present	200,000	0.8457	0.7200
confidence_0.95_0.05	confidence_threshold	test_temporal	fee_calc_nonzero	200,000	0.9753	0.8273
confidence_0.95_0.05	confidence_threshold	test_spatial	trade_failure	200,000	0.9676	0.7928
confidence_0.95_0.05	confidence_threshold	test_spatial	special_present	200,000	0.7921	0.6370
confidence_0.95_0.05	confidence_threshold	test_spatial	fee_calc_nonzero	200,000	0.9707	0.7792
confidence_0.95_0.05	confidence_threshold	test_joint	trade_failure	158,896	0.9664	0.8208
confidence_0.95_0.05	confidence_threshold	test_joint	special_present	158,896	0.8237	0.6867
confidence_0.95_0.05	confidence_threshold	test_joint	fee_calc_nonzero	158,896	0.9717	0.8139
seed_20221214	random_seed	test_temporal	trade_failure	200,000	0.8499	0.4382
seed_20221214	random_seed	test_temporal	special_present	200,000	0.7541	0.4001
seed_20221214	random_seed	test_temporal	fee_calc_nonzero	200,000	0.8534	0.4300
seed_20221214	random_seed	test_spatial	trade_failure	200,000	0.8762	0.4478
seed_20221214	random_seed	test_spatial	special_present	200,000	0.7245	0.3907
seed_20221214	random_seed	test_spatial	fee_calc_nonzero	200,000	0.8791	0.4384
seed_20221214	random_seed	test_joint	trade_failure	158,896	0.8368	0.4119
seed_20221214	random_seed	test_joint	special_present	158,896	0.7311	0.3883
seed_20221214	random_seed	test_joint	fee_calc_nonzero	158,896	0.8402	0.4065
seed_20221215	random_seed	test_temporal	trade_failure	200,000	0.9730	0.8409
seed_20221215	random_seed	test_temporal	special_present	200,000	0.8482	0.7238
seed_20221215	random_seed	test_temporal	fee_calc_nonzero	200,000	0.9785	0.8343
seed_20221215	random_seed	test_spatial	trade_failure	200,000	0.9706	0.7979
seed_20221215	random_seed	test_spatial	special_present	200,000	0.7944	0.6420
seed_20221215	random_seed	test_spatial	fee_calc_nonzero	200,000	0.9745	0.7951
seed_20221215	random_seed	test_joint	trade_failure	158,896	0.9688	0.8188
seed_20221215	random_seed	test_joint	special_present	158,896	0.8249	0.6899
seed_20221215	random_seed	test_joint	fee_calc_nonzero	158,896	0.9748	0.8187

Table S3D. Tie-aware enrichment for all methods, roles, and proxies.

Role	Proxy	Method	Requested fraction	Inclusive cutoff	Selected records	Achieved fraction	Baseline rate	Selected proxy rate	Lift
test_temporal	trade_failure	Hard-rule union	0.0100	1.0000	72,558	0.0930	0.1580	0.4044	2.5587
test_temporal	trade_failure	Hard-rule union	0.0500	1.0000	72,558	0.0930	0.1580	0.4044	2.5587

test_temporal	trade_failure	Hard-rule union	0.1000	0.0000	779,896	1.0000	0.1580	0.1580	1.0000
test_temporal	trade_failure	Anomaly-vote fraction	0.0100	0.2000	8,019	0.0103	0.1580	0.7672	4.8547
test_temporal	trade_failure	Anomaly-vote fraction	0.0500	0.1000	72,558	0.0930	0.1580	0.4044	2.5587
test_temporal	trade_failure	Anomaly-vote fraction	0.1000	0.0000	779,896	1.0000	0.1580	0.1580	1.0000
test_temporal	trade_failure	Probabilistic label model	0.0100	1.0000	8,013	0.0103	0.1580	0.7675	4.8568
test_temporal	trade_failure	Probabilistic label model	0.0500	1.0000	50,927	0.0653	0.1580	0.3205	2.0279
test_temporal	trade_failure	Probabilistic label model	0.1000	0.5686	238,926	0.3064	0.1580	0.5100	3.2274
test_temporal	trade_failure	Isolation Forest	0.0100	0.9925	7,822	0.0100	0.1580	0.8479	5.3653
test_temporal	trade_failure	Isolation Forest	0.0500	0.9724	39,007	0.0500	0.1580	0.8264	5.2294
test_temporal	trade_failure	Isolation Forest	0.1000	0.9406	78,014	0.1000	0.1580	0.7429	4.7011
test_temporal	trade_failure	CAWS-ETC	0.0100	0.9956	8,123	0.0104	0.1580	0.9607	6.0795
test_temporal	trade_failure	CAWS-ETC	0.0500	0.9955	39,475	0.0506	0.1580	0.8795	5.5654
test_temporal	trade_failure	CAWS-ETC	0.1000	0.9954	120,723	0.1548	0.1580	0.9515	6.0208
test_temporal	special_present	Hard-rule union	0.0100	1.0000	72,558	0.0930	0.2079	0.4299	2.0683
test_temporal	special_present	Hard-rule union	0.0500	1.0000	72,558	0.0930	0.2079	0.4299	2.0683
test_temporal	special_present	Hard-rule union	0.1000	0.0000	779,896	1.0000	0.2079	0.2079	1.0000
test_temporal	special_present	Anomaly-vote fraction	0.0100	0.2000	8,019	0.0103	0.2079	0.7861	3.7821
test_temporal	special_present	Anomaly-vote fraction	0.0500	0.1000	72,558	0.0930	0.2079	0.4299	2.0683
test_temporal	special_present	Anomaly-vote fraction	0.1000	0.0000	779,896	1.0000	0.2079	0.2079	1.0000
test_temporal	special_present	Probabilistic label model	0.0100	1.0000	8,013	0.0103	0.2079	0.7865	3.7837
test_temporal	special_present	Probabilistic label model	0.0500	1.0000	50,927	0.0653	0.2079	0.3476	1.6721
test_temporal	special_present	Probabilistic label model	0.1000	0.5686	238,926	0.3064	0.2079	0.5282	2.5412
test_temporal	special_present	Isolation Forest	0.0100	0.9925	7,822	0.0100	0.2079	0.8653	4.1627
test_temporal	special_present	Isolation Forest	0.0500	0.9724	39,007	0.0500	0.2079	0.8441	4.0611
test_temporal	special_present	Isolation Forest	0.1000	0.9406	78,014	0.1000	0.2079	0.7687	3.6981
test_temporal	special_present	CAWS-ETC	0.0100	0.9956	8,123	0.0104	0.2079	0.9791	4.7103
test_temporal	special_present	CAWS-ETC	0.0500	0.9955	39,475	0.0506	0.2079	0.8925	4.2940
test_temporal	special_present	CAWS-ETC	0.1000	0.9954	120,723	0.1548	0.2079	0.9575	4.6067
test_temporal	fee_calc_nonzero	Hard-rule union	0.0100	1.0000	72,558	0.0930	0.1520	0.3574	2.3518
test_temporal	fee_calc_nonzero	Hard-rule union	0.0500	1.0000	72,558	0.0930	0.1520	0.3574	2.3518
test_temporal	fee_calc_nonzero	Hard-rule union	0.1000	0.0000	779,896	1.0000	0.1520	0.1520	1.0000

test_temporal	fee_calc_nonzero	Anomaly-vote fraction	0.0100	0.2000	8,019	0.0103	0.1520	0.6687	4.4000
test_temporal	fee_calc_nonzero	Anomaly-vote fraction	0.0500	0.1000	72,558	0.0930	0.1520	0.3574	2.3518
test_temporal	fee_calc_nonzero	Anomaly-vote fraction	0.1000	0.0000	779,896	1.0000	0.1520	0.1520	1.0000
test_temporal	fee_calc_nonzero	Probabilistic label model	0.0100	1.0000	8,013	0.0103	0.1520	0.6689	4.4016
test_temporal	fee_calc_nonzero	Probabilistic label model	0.0500	1.0000	50,927	0.0653	0.1520	0.3025	1.9906
test_temporal	fee_calc_nonzero	Probabilistic label model	0.1000	0.5686	238,926	0.3064	0.1520	0.4961	3.2642
test_temporal	fee_calc_nonzero	Isolation Forest	0.0100	0.9925	7,822	0.0100	0.1520	0.8062	5.3049
test_temporal	fee_calc_nonzero	Isolation Forest	0.0500	0.9724	39,007	0.0500	0.1520	0.7862	5.1737
test_temporal	fee_calc_nonzero	Isolation Forest	0.1000	0.9406	78,014	0.1000	0.1520	0.7147	4.7028
test_temporal	fee_calc_nonzero	CAWS-ETC	0.0100	0.9956	8,123	0.0104	0.1520	0.8737	5.7491
test_temporal	fee_calc_nonzero	CAWS-ETC	0.0500	0.9955	39,475	0.0506	0.1520	0.8115	5.3399
test_temporal	fee_calc_nonzero	CAWS-ETC	0.1000	0.9954	120,723	0.1548	0.1520	0.9303	6.1219
test_spatial	trade_failure	Hard-rule union	0.0100	1.0000	146,137	0.0897	0.1381	0.3910	2.8322
test_spatial	trade_failure	Hard-rule union	0.0500	1.0000	146,137	0.0897	0.1381	0.3910	2.8322
test_spatial	trade_failure	Hard-rule union	0.1000	0.0000	1,628,850	1.0000	0.1381	0.1381	1.0000
test_spatial	trade_failure	Anomaly-vote fraction	0.0100	0.2000	26,055	0.0160	0.1381	0.5766	4.1768
test_spatial	trade_failure	Anomaly-vote fraction	0.0500	0.1000	146,137	0.0897	0.1381	0.3910	2.8322
test_spatial	trade_failure	Anomaly-vote fraction	0.1000	0.0000	1,628,850	1.0000	0.1381	0.1381	1.0000
test_spatial	trade_failure	Probabilistic label model	0.0100	1.0000	25,605	0.0157	0.1381	0.5737	4.1555
test_spatial	trade_failure	Probabilistic label model	0.0500	1.0000	95,097	0.0584	0.1381	0.3566	2.5834
test_spatial	trade_failure	Probabilistic label model	0.1000	0.5686	438,158	0.2690	0.1381	0.5082	3.6814
test_spatial	trade_failure	Isolation Forest	0.0100	0.9919	16,318	0.0100	0.1381	0.6779	4.9104
test_spatial	trade_failure	Isolation Forest	0.0500	0.9508	81,461	0.0500	0.1381	0.6528	4.7284
test_spatial	trade_failure	Isolation Forest	0.1000	0.9003	162,900	0.1000	0.1381	0.6344	4.5950
test_spatial	trade_failure	CAWS-ETC	0.0100	0.9956	20,763	0.0127	0.1381	0.9315	6.7471
test_spatial	trade_failure	CAWS-ETC	0.0500	0.9955	81,596	0.0501	0.1381	0.8119	5.8808
test_spatial	trade_failure	CAWS-ETC	0.1000	0.9954	238,337	0.1463	0.1381	0.8975	6.5010
test_spatial	special_present	Hard-rule union	0.0100	1.0000	146,137	0.0897	0.2195	0.4338	1.9761
test_spatial	special_present	Hard-rule union	0.0500	1.0000	146,137	0.0897	0.2195	0.4338	1.9761
test_spatial	special_present	Hard-rule union	0.1000	0.0000	1,628,850	1.0000	0.2195	0.2195	1.0000
test_spatial	special_present	Anomaly-vote fraction	0.0100	0.2000	26,055	0.0160	0.2195	0.5883	2.6798

test_spatial	special_present	Anomaly-vote fraction	0.0500	0.1000	146,137	0.0897	0.2195	0.4338	1.9761
test_spatial	special_present	Anomaly-vote fraction	0.1000	0.0000	1,628,850	1.0000	0.2195	0.2195	1.0000
test_spatial	special_present	Probabilistic label model	0.0100	1.0000	25,605	0.0157	0.2195	0.5834	2.6577
test_spatial	special_present	Probabilistic label model	0.0500	1.0000	95,097	0.0584	0.2195	0.3951	1.7999
test_spatial	special_present	Probabilistic label model	0.1000	0.5686	438,158	0.2690	0.2195	0.5397	2.4585
test_spatial	special_present	Isolation Forest	0.0100	0.9919	16,318	0.0100	0.2195	0.7611	3.4673
test_spatial	special_present	Isolation Forest	0.0500	0.9508	81,461	0.0500	0.2195	0.7038	3.2062
test_spatial	special_present	Isolation Forest	0.1000	0.9003	162,900	0.1000	0.2195	0.7036	3.2052
test_spatial	special_present	CAWS-ETC	0.0100	0.9956	20,763	0.0127	0.2195	0.9481	4.3192
test_spatial	special_present	CAWS-ETC	0.0500	0.9955	81,596	0.0501	0.2195	0.8342	3.8001
test_spatial	special_present	CAWS-ETC	0.1000	0.9954	238,337	0.1463	0.2195	0.9085	4.1388
test_spatial	fee_calc_nonzero	Hard-rule union	0.0100	1.0000	146,137	0.0897	0.1330	0.3474	2.6108
test_spatial	fee_calc_nonzero	Hard-rule union	0.0500	1.0000	146,137	0.0897	0.1330	0.3474	2.6108
test_spatial	fee_calc_nonzero	Hard-rule union	0.1000	0.0000	1,628,850	1.0000	0.1330	0.1330	1.0000
test_spatial	fee_calc_nonzero	Anomaly-vote fraction	0.0100	0.2000	26,055	0.0160	0.1330	0.5348	4.0195
test_spatial	fee_calc_nonzero	Anomaly-vote fraction	0.0500	0.1000	146,137	0.0897	0.1330	0.3474	2.6108
test_spatial	fee_calc_nonzero	Anomaly-vote fraction	0.1000	0.0000	1,628,850	1.0000	0.1330	0.1330	1.0000
test_spatial	fee_calc_nonzero	Probabilistic label model	0.0100	1.0000	25,605	0.0157	0.1330	0.5426	4.0784
test_spatial	fee_calc_nonzero	Probabilistic label model	0.0500	1.0000	95,097	0.0584	0.1330	0.3429	2.5770
test_spatial	fee_calc_nonzero	Probabilistic label model	0.1000	0.5686	438,158	0.2690	0.1330	0.4946	3.7174
test_spatial	fee_calc_nonzero	Isolation Forest	0.0100	0.9919	16,318	0.0100	0.1330	0.6585	4.9496
test_spatial	fee_calc_nonzero	Isolation Forest	0.0500	0.9508	81,461	0.0500	0.1330	0.6260	4.7054
test_spatial	fee_calc_nonzero	Isolation Forest	0.1000	0.9003	162,900	0.1000	0.1330	0.6167	4.6349
test_spatial	fee_calc_nonzero	CAWS-ETC	0.0100	0.9956	20,763	0.0127	0.1330	0.8809	6.6212
test_spatial	fee_calc_nonzero	CAWS-ETC	0.0500	0.9955	81,596	0.0501	0.1330	0.7491	5.6306
test_spatial	fee_calc_nonzero	CAWS-ETC	0.1000	0.9954	238,337	0.1463	0.1330	0.8769	6.5910
test_joint	trade_failure	Hard-rule union	0.0100	1.0000	15,916	0.1002	0.1533	0.3500	2.2836
test_joint	trade_failure	Hard-rule union	0.0500	1.0000	15,916	0.1002	0.1533	0.3500	2.2836
test_joint	trade_failure	Hard-rule union	0.1000	1.0000	15,916	0.1002	0.1533	0.3500	2.2836
test_joint	trade_failure	Anomaly-vote fraction	0.0100	0.2000	1,649	0.0104	0.1533	0.7495	4.8910
test_joint	trade_failure	Anomaly-vote fraction	0.0500	0.1000	15,916	0.1002	0.1533	0.3500	2.2836

test_joint	trade_failure	Anomaly-vote fraction	0.1000	0.1000	15,916	0.1002	0.1533	0.3500	2.2836
test_joint	trade_failure	Probabilistic label model	0.0100	1.0000	1,612	0.0101	0.1533	0.7463	4.8696
test_joint	trade_failure	Probabilistic label model	0.0500	1.0000	11,174	0.0703	0.1533	0.3089	2.0159
test_joint	trade_failure	Probabilistic label model	0.1000	0.9999	15,914	0.1002	0.1533	0.3499	2.2835
test_joint	trade_failure	Isolation Forest	0.0100	0.9954	1,591	0.0100	0.1533	0.5563	3.6297
test_joint	trade_failure	Isolation Forest	0.0500	0.9740	7,952	0.0500	0.1533	0.6957	4.5394
test_joint	trade_failure	Isolation Forest	0.1000	0.9425	15,895	0.1000	0.1533	0.6831	4.4574
test_joint	trade_failure	CAWS-ETC	0.0100	0.9956	1,762	0.0111	0.1533	0.8859	5.7809
test_joint	trade_failure	CAWS-ETC	0.0500	0.9955	8,146	0.0513	0.1533	0.8087	5.2772
test_joint	trade_failure	CAWS-ETC	0.1000	0.9954	24,500	0.1542	0.1533	0.9213	6.0120
test_joint	special_present	Hard-rule union	0.0100	1.0000	15,916	0.1002	0.2203	0.4067	1.8458
test_joint	special_present	Hard-rule union	0.0500	1.0000	15,916	0.1002	0.2203	0.4067	1.8458
test_joint	special_present	Hard-rule union	0.1000	1.0000	15,916	0.1002	0.2203	0.4067	1.8458
test_joint	special_present	Anomaly-vote fraction	0.0100	0.2000	1,649	0.0104	0.2203	0.7683	3.4871
test_joint	special_present	Anomaly-vote fraction	0.0500	0.1000	15,916	0.1002	0.2203	0.4067	1.8458
test_joint	special_present	Anomaly-vote fraction	0.1000	0.1000	15,916	0.1002	0.2203	0.4067	1.8458
test_joint	special_present	Probabilistic label model	0.0100	1.0000	1,612	0.0101	0.2203	0.7643	3.4686
test_joint	special_present	Probabilistic label model	0.0500	1.0000	11,174	0.0703	0.2203	0.3677	1.6689
test_joint	special_present	Probabilistic label model	0.1000	0.9999	15,914	0.1002	0.2203	0.4067	1.8457
test_joint	special_present	Isolation Forest	0.0100	0.9954	1,591	0.0100	0.2203	0.7228	3.2805
test_joint	special_present	Isolation Forest	0.0500	0.9740	7,952	0.0500	0.2203	0.7534	3.4193
test_joint	special_present	Isolation Forest	0.1000	0.9425	15,895	0.1000	0.2203	0.7469	3.3898
test_joint	special_present	CAWS-ETC	0.0100	0.9956	1,762	0.0111	0.2203	0.9126	4.1418
test_joint	special_present	CAWS-ETC	0.0500	0.9955	8,146	0.0513	0.2203	0.8300	3.7668
test_joint	special_present	CAWS-ETC	0.1000	0.9954	24,500	0.1542	0.2203	0.9304	4.2228
test_joint	fee_calc_nonzero	Hard-rule union	0.0100	1.0000	15,916	0.1002	0.1485	0.3180	2.1418
test_joint	fee_calc_nonzero	Hard-rule union	0.0500	1.0000	15,916	0.1002	0.1485	0.3180	2.1418
test_joint	fee_calc_nonzero	Hard-rule union	0.1000	1.0000	15,916	0.1002	0.1485	0.3180	2.1418
test_joint	fee_calc_nonzero	Anomaly-vote fraction	0.0100	0.2000	1,649	0.0104	0.1485	0.6901	4.6484
test_joint	fee_calc_nonzero	Anomaly-vote fraction	0.0500	0.1000	15,916	0.1002	0.1485	0.3180	2.1418
test_joint	fee_calc_nonzero	Anomaly-vote fraction	0.1000	0.1000	15,916	0.1002	0.1485	0.3180	2.1418

test_joint	fee_calc_nonzero	Probabilistic label model	0.0100	1.0000	1,612	0.0101	0.1485	0.7053	4.7509
test_joint	fee_calc_nonzero	Probabilistic label model	0.0500	1.0000	11,174	0.0703	0.1485	0.2986	2.0110
test_joint	fee_calc_nonzero	Probabilistic label model	0.1000	0.9999	15,914	0.1002	0.1485	0.3180	2.1417
test_joint	fee_calc_nonzero	Isolation Forest	0.0100	0.9954	1,591	0.0100	0.1485	0.5431	3.6579
test_joint	fee_calc_nonzero	Isolation Forest	0.0500	0.9740	7,952	0.0500	0.1485	0.6639	4.4716
test_joint	fee_calc_nonzero	Isolation Forest	0.1000	0.9425	15,895	0.1000	0.1485	0.6640	4.4724
test_joint	fee_calc_nonzero	CAWS-ETC	0.0100	0.9956	1,762	0.0111	0.1485	0.8235	5.5469
test_joint	fee_calc_nonzero	CAWS-ETC	0.0500	0.9955	8,146	0.0513	0.1485	0.7591	5.1134
test_joint	fee_calc_nonzero	CAWS-ETC	0.1000	0.9954	24,500	0.1542	0.1485	0.9059	6.1018

Table S4A. Complete 46-feature input dictionary.

No.	Feature	View/family	Data type	Definition
1	gantry_prefix	Contextual-semantic	Categorical	Coarse G/S gantry family; raw gantry identifier excluded.
2	hour_sin	Temporal	Numeric	Sine encoding of transaction hour.
3	hour_cos	Temporal	Numeric	Cosine encoding of transaction hour.
4	signed_log_pay_fee	Monetary	Numeric	Signed log1p transform of payable fee.
5	signed_log_discount_fee	Monetary	Numeric	Signed log1p transform of discount fee.
6	signed_log_net_fee	Monetary	Numeric	Signed log1p transform of net fee.
7	discount_rate	Monetary	Numeric	Discount fee divided by payable fee when payable fee is positive.
8	abs_pay_residual	Monetary	Numeric	Absolute payable-total residual.
9	abs_discount_residual	Monetary	Numeric	Absolute discount-total residual.
10	abs_net_residual	Monetary	Numeric	Absolute net-total residual.
11	toll_interval_count	Structural-pattern	Numeric	Number of toll-interval components.
12	pay_group_count	Structural-pattern	Numeric	Number of payable-fee components.
13	discount_group_count	Structural-pattern	Numeric	Number of discount components.
14	fee_group_count	Structural-pattern	Numeric	Number of net-fee components.
15	group_count_spread	Structural-pattern	Numeric	Maximum minus minimum available component-array count.
16	pay_group_missing	Structural-pattern	Binary	Payable component array missing.
17	discount_group_missing	Structural-pattern	Binary	Discount component array missing.
18	fee_group_missing	Structural-pattern	Binary	Net-fee component array missing.
19	any_group_parse_error	Structural-pattern	Binary	At least one non-missing component array cannot be parsed.
20	entry_time_valid	Temporal	Binary	Entry timestamp satisfies plausibility and ordering criteria.

21	previous_time_valid	Temporal	Binary	Previous timestamp satisfies plausibility and ordering criteria.
22	log_entry_duration	Temporal	Numeric	log1p of valid entry-to-transaction duration.
23	log_previous_duration	Temporal	Numeric	log1p of valid previous-to-transaction duration.
24	last_gantry_present	Structural-pattern	Binary	Previous-gantry field is available.
25	opposite_gantry_present	Structural-pattern	Binary	Opposite-gantry field is available.
26	previous_time_conflict	Temporal	Binary	Previous gantry is present but timestamp invalid.
27	media_type	Contextual-semantic	Categorical	Transaction medium category.
28	vehicle_type	Contextual-semantic	Categorical	Recorded vehicle-type code.
29	fee_vehicle_type	Contextual-semantic	Categorical	Billing vehicle-type code.
30	vehicle_class	Contextual-semantic	Categorical	Vehicle-class code.
31	vehicle_type_disagreement	Contextual-semantic	Binary	Recorded and billing vehicle types differ.
32	plate_color	Contextual-semantic	Categorical	Non-identifying plate-color suffix.
33	vehicle_type_out_of_dictionary	Contextual-semantic	Binary	Recorded vehicle type outside documented code set.
34	fee_vehicle_type_out_of_dictionary	Contextual-semantic	Binary	Billing vehicle type outside documented code set.
35	vehicle_class_out_of_dictionary	Contextual-semantic	Binary	Vehicle class outside documented code set.
36	pay_context_z	Monetary	Numeric	Robust contextual deviation of payable fee.
37	discount_context_z	Monetary	Numeric	Robust contextual deviation of discount fee.
38	net_context_z	Monetary	Numeric	Robust contextual deviation of net fee.
39	entry_duration_z	Temporal	Numeric	Robust contextual deviation of entry duration.
40	previous_duration_z	Temporal	Numeric	Robust contextual deviation of previous-gantry duration.
41	pair_train_count	Contextual-semantic	Numeric	Training frequency of vehicle-type pair.
42	pair_rarity_score	Contextual-semantic	Numeric	Log inverse-frequency score of vehicle-type pair.
43	class_train_count	Contextual-semantic	Numeric	Training frequency of vehicle-class code.
44	class_rarity_score	Contextual-semantic	Numeric	Log inverse-frequency score of vehicle-class code.
45	pattern_train_count	Structural-pattern	Numeric	Training frequency of missingness/validity pattern.
46	pattern_rarity_score	Structural-pattern	Numeric	Log inverse-frequency score of missingness/validity pattern.

Table S4B. Global LightGBM tree-contribution summary.

Rank	Feature	Mean absolute contribution	Mean signed contribution	Share of total absolute contribution (%)
1	entry_duration_z	2.0561	-0.5372	40.4514
2	abs_pay_residual	0.9587	0.6632	18.8622
3	log_entry_duration	0.6204	-0.1556	12.2055

4	previous_duration_z	0.5947	-0.1724	11.7003
5	abs_net_residual	0.2723	0.1957	5.3575
6	fee_vehicle_type	0.1597	-0.0371	3.1414
7	log_previous_duration	0.1147	-0.0351	2.2575
8	hour_cos	0.0568	0.0568	1.1166
9	pattern_train_count	0.0393	0.0217	0.7729
10	signed_log_pay_fee	0.0345	0.0027	0.6789
11	vehicle_type	0.0312	0.0013	0.6143
12	pay_group_count	0.0276	0.0068	0.5430
13	pair_train_count	0.0268	-0.0013	0.5272
14	abs_discount_residual	0.0176	0.0163	0.3460
15	hour_sin	0.0126	-0.0045	0.2478
16	signed_log_net_fee	0.0072	0.0047	0.1423
17	signed_log_discount_fee	0.0067	-0.0025	0.1314
18	previous_time_valid	0.0065	-0.0007	0.1270
19	discount_rate	0.0060	-0.0028	0.1182
20	toll_interval_count	0.0054	0.0005	0.1065
21	pair_rarity_score	0.0053	-0.0020	0.1041
22	class_train_count	0.0034	0.0003	0.0667
23	vehicle_class	0.0028	-0.0011	0.0556
24	plate_color	0.0021	-0.0008	0.0413
25	vehicle_type_disagreement	0.0021	0.0003	0.0409
26	last_gantry_present	0.0021	0.0013	0.0407
27	pay_context_z	0.0020	0.0010	0.0395
28	media_type	0.0019	0.0001	0.0366
29	pattern_rarity_score	0.0016	-0.0001	0.0316
30	discount_context_z	0.0016	0.0010	0.0308
31	class_rarity_score	0.0012	-0.0004	0.0230
32	net_context_z	0.0008	0.0002	0.0166
33	previous_time_conflict	0.0005	-0.0000	0.0095
34	gantry_prefix	0.0004	0.0001	0.0085

35	vehicle_class_out_of_dictionary	0.0002	0.0001	0.0034
36	opposite_gantry_present	0.0002	0.0001	0.0031
37	fee_vehicle_type_out_of_dictionary	0.0000	0.0000	0.0000
38	vehicle_type_out_of_dictionary	0.0000	0.0000	0.0000
39	any_group_parse_error	0.0000	0.0000	0.0000
40	fee_group_missing	0.0000	0.0000	0.0000
41	discount_group_missing	0.0000	0.0000	0.0000
42	pay_group_missing	0.0000	0.0000	0.0000
43	group_count_spread	0.0000	0.0000	0.0000
44	fee_group_count	0.0000	0.0000	0.0000
45	entry_time_valid	0.0000	0.0000	0.0000
46	discount_group_count	0.0000	0.0000	0.0000

Table S4C. Binned signed tree contributions.

Feature	Quantile bin	Records	Mean feature value	Mean signed contribution
entry_duration_z	1	4,937	0.0803	-1.5825
entry_duration_z	2	4,937	0.2372	-1.5732
entry_duration_z	3	4,937	0.3926	-1.5678
entry_duration_z	4	4,937	0.5300	-1.5826
entry_duration_z	5	4,944	0.6503	-1.6133
entry_duration_z	6	4,930	0.7958	-1.5878
entry_duration_z	7	4,937	1.3882	-1.5526
entry_duration_z	8	4,937	2.4514	-1.5641
entry_duration_z	9	4,937	4.5348	2.2133
entry_duration_z	10	4,938	136.8756	4.7104
abs_pay_residual	5	42,332	0.0000	-0.1745
abs_pay_residual	10	195	1,431.559	5.4052
log_entry_duration	1	4,939	3.7184	-0.5867
log_entry_duration	2	4,934	6.9155	-0.5484
log_entry_duration	3	4,938	7.7070	-0.5272

log_entry_duration	4	4,937	8.2415	-0.5060
log_entry_duration	5	4,937	8.6651	-0.4866
log_entry_duration	6	4,938	9.0414	-0.4803
log_entry_duration	7	4,936	9.3876	-0.4603
log_entry_duration	8	4,939	9.7306	-0.1435
log_entry_duration	9	4,936	10.1340	0.8857
log_entry_duration	10	4,938	11.4174	1.2131
previous_duration_z	1	4,214	0.0741	-0.4405
previous_duration_z	2	4,218	0.2363	-0.4391
previous_duration_z	3	4,210	0.4054	-0.4418
previous_duration_z	4	4,219	0.5561	-0.4430
previous_duration_z	5	4,230	0.6590	-0.4317
previous_duration_z	6	4,204	0.7428	-0.4420
previous_duration_z	7	4,206	0.8673	-0.4534
previous_duration_z	8	4,218	1.1842	-0.4452
previous_duration_z	9	4,213	2.1372	-0.4513
previous_duration_z	10	4,215	8,139.101	2.3092
abs_net_residual	5	42,332	0.0000	-0.0453
abs_net_residual	10	195	1,345.487	1.5821
log_previous_duration	1	4,157	4.2469	-0.0738
log_previous_duration	2	4,336	4.8995	-0.0857
log_previous_duration	3	4,177	5.1405	-0.0882
log_previous_duration	4	4,184	5.4091	-0.0842
log_previous_duration	5	4,245	5.7224	-0.0830
log_previous_duration	6	4,212	5.9630	-0.0858
log_previous_duration	7	4,183	6.1729	-0.0853
log_previous_duration	8	4,224	6.4275	-0.0866
log_previous_duration	9	4,220	6.7635	-0.0852
log_previous_duration	10	4,210	8.2386	0.4381

hour_cos	3	20,577	0.7071	0.0575
hour_cos	6	16,718	0.8660	0.0536
hour_cos	9	12,705	0.9659	0.0597
pattern_train_count	1	5,195	151,625.246	0.2327
pattern_train_count	2	4,670	281,890.731	-0.0188
pattern_train_count	4	10,999	949,170.000	-0.0235
pattern_train_count	6	11,027	1,911,656.000	0.0079
pattern_train_count	9	18,109	3,305,442.000	0.0074
signed_log_pay_fee	1	2	-6.1513	0.0662
signed_log_pay_fee	2	11,153	0.0000	0.0816
signed_log_pay_fee	3	3,827	5.0198	-0.0216
signed_log_pay_fee	4	5,160	5.7087	-0.0273
signed_log_pay_fee	5	4,832	6.0770	-0.0248
signed_log_pay_fee	6	5,007	6.4221	-0.0267
signed_log_pay_fee	7	4,967	6.8183	-0.0230
signed_log_pay_fee	8	5,050	7.2596	-0.0138
signed_log_pay_fee	9	4,945	7.6571	-0.0126
signed_log_pay_fee	10	5,057	8.3911	-0.0106
pay_group_count	4	33,544	1.0000	-0.0155
pay_group_count	9	6,115	2.0000	0.0127
pay_group_count	10	2,868	5.0645	0.0244

Table S4D. Gantry-time burden sensitivity to minimum cell size.

Minimum transactions per cell	Retained windows	Mean burden	SD burden	Median burden	95th percentile	99th percentile
5	203,857	0.2279	0.1956	0.2034	0.5446	0.9947
10	177,651	0.2360	0.1927	0.2135	0.5353	0.9949
20	142,614	0.2498	0.1904	0.2292	0.5353	0.9950

Table S5A. Weak-label posterior distributions by analytical role.

Role	Records	Mean	P01	P05	P25	P50	P75	P95	P99	p~ >= 0.90	p~ <= 0.10	Any anomaly vote	Joint normal anchor
Auxiliary	441,675	0.2079	0.0000	0.0000	0.0000	0.0000	0.5686	1.0000	1.0000	40,943	311,218	40,943	311,218
Joint-transfer test	158,896	0.2249	0.0000	0.0000	0.0000	0.0000	0.5686	1.0000	1.0000	15,916	108,133	15,916	108,133
Spatial-transfer test	1,628,850	0.1917	0.0000	0.0000	0.0000	0.0000	0.5686	1.0000	1.0000	146,137	1,190,692	146,137	1,190,692
Temporal-transfer test	779,896	0.2143	0.0000	0.0000	0.0000	0.0000	0.5686	1.0000	1.0000	72,558	540,970	72,558	540,970
Core training	8,079,105	0.1849	0.0000	0.0000	0.0000	0.0000	0.5686	1.0000	1.0000	682,628	5,970,006	682,628	5,970,006
Joint validation	289,517	0.1948	0.0000	0.0000	0.0000	0.0000	0.5686	1.0000	1.0000	25,251	209,498	25,251	209,498
Spatial validation	1,765,005	0.1789	0.0000	0.0000	0.0000	0.0000	0.0000	1.0000	1.0000	153,468	1,326,232	153,468	1,326,232
Temporal validation	1,367,903	0.1960	0.0000	0.0000	0.0000	0.0000	0.5686	1.0000	1.0000	114,070	982,901	114,070	982,901

Table S5B. CAWS-ETC score distributions by analytical role.

Role	Records	Mean score	SD	P01	P05	P25	P50	P75	P95	P99	Correlation with weak-label posterior
Auxiliary	441,675	0.2784	0.4390	0.0045	0.0049	0.0053	0.0060	0.9488	0.9955	0.9956	0.9258
Joint-transfer test	158,896	0.3001	0.4490	0.0045	0.0050	0.0054	0.0067	0.9923	0.9955	0.9956	0.9227
Spatial-transfer test	1,628,850	0.2523	0.4254	0.0044	0.0047	0.0050	0.0054	0.9082	0.9955	0.9956	0.9270
Temporal-transfer test	779,896	0.2899	0.4447	0.0045	0.0049	0.0054	0.0065	0.9916	0.9955	0.9956	0.9260
Core training	8,079,105	0.2469	0.4225	0.0044	0.0044	0.0050	0.0054	0.0445	0.9955	0.9956	0.9300
Joint validation	289,517	0.2618	0.4306	0.0044	0.0045	0.0053	0.0058	0.9240	0.9955	0.9956	0.9290
Spatial validation	1,765,005	0.2353	0.4155	0.0044	0.0044	0.0050	0.0053	0.0134	0.9955	0.9956	0.9315
Temporal validation	1,367,903	0.2668	0.4331	0.0044	0.0045	0.0053	0.0058	0.9319	0.9955	0.9956	0.9289

Table S5C. Computational timings.

Computational procedure	Rows/records processed	Elapsed time (s)	Throughput (records/s)	Peak memory (MB)	Notes
Initial streaming CSV audit	14,510,847	873.8930	16,604.840	25.7900	Single-pass CSV audit
Refined columnar audit	14,510,847	28.9160			Parquet/DuckDB audit
Spatial-temporal partitioning	14,510,847	13.7270			Leakage-resistant split
Feature construction	14,510,847	16.9130			Training-only reference statistics
Weak-source generation	14,510,847	6.4570			14 candidate functions

Probabilistic weak-label learning/scoring	14,510,847	61.3880		500,000-record fit sample
LightGBM discriminator training	600,000	16.8800		CPU; n_jobs=4
Complete-data scoring and aggregation	14,510,847	87.3430		233,476 cells
Rule-aligned intervention generation	432,000	46.1650		216,000 matched pairs
Comparator scoring	432,000	94.6450		Five methods
Rule-aligned evaluation	432,000	56.4210		100 bootstrap resamples
Rule-orthogonal robustness/ablation/interpretability	216,000	216.0630		Multiple retrained variants

Table S5D. Software requirements specified by the workflow.

Software	Compatible version range	Primary use
DuckDB	>=1.1,<2.0	Local Parquet conversion and analytical queries
NumPy	>=1.24,<3.0	Numerical computation
pandas	>=2.0,<3.0	Model samples and evaluation tables
LightGBM	>=4.0,<5.0	Discriminative ranking model and tree contributions
scikit-learn	>=1.3,<2.0	Isolation Forest and evaluation metrics
psutil	>=5.9,<8.0	Runtime and memory diagnostics
matplotlib	>=3.7,<4.0	Figure generation

Table S6. Independent proxy association and tie-aware high-score enrichment.

Operational proxy	Temporal AUROC	Temporal AUPRC	Spatial AUROC	Spatial AUPRC	Joint AUROC	Joint AUPRC	Joint baseline rate	Top-1% achieved fraction	Top-1% lift	Top-5% achieved fraction	Top-5% lift
Transaction failure	0.9850	0.9176	0.9810	0.8599	0.9796	0.8674	0.1533	0.0111	5.7809	0.0513	5.2772
Special-transaction status	0.8477	0.7863	0.7869	0.6800	0.8226	0.7289	0.2203	0.0111	4.1418	0.0513	3.7668
Nonzero fee-calculation status	0.9875	0.8894	0.9823	0.8348	0.9830	0.8478	0.1485	0.0111	5.5469	0.0513	5.1134

Note: Proxy outcomes provide association/enrichment evidence only.

Table S7. Independent proxy association and tie-aware high-score enrichment. (The parser safeguard had zero empirical coverage and was excluded from probabilistic anomaly-source fitting. It was nevertheless retained in the deterministic hard-rule and vote-fraction comparators. The parser intervention also removed the monetary-normal component of the joint normal anchor; consequently, label-model separation of parser-control pairs reflects loss of normal-anchor evidence rather than learning from parser-error examples.)

Panel A

Mechanism	Additional base eligibility	Mild	Moderate	Severe	Principal modified features
Monetary total mismatch	pay_fee, pay_group_sum, fee_group_sum available	1%	5%	20%	delta=max(1,abs(pay_fee)×fraction); payable/net residuals and absolute residuals=delta; associated component sums=total-delta
Discount total mismatch	discount_fee, discount_group_sum available	0.5%	2%	10%	delta=max(1,abs(pay_fee)×fraction); discount residual and absolute residual=delta; discount_group_sum=discount_fee-delta
Entry time invalid	none beyond base eligibility	binary	binary	binary	entry_time_valid=False; entry duration, log duration, and deviation set missing
Previous-time conflict	none beyond base eligibility	binary	binary	binary	previous gantry present=True; previous validity=False; conflict=True; corresponding duration variables missing
Entry-duration outlier	entry-duration features available	z=8.5; log +0.50	z=12; log +1.00	z=20; log +2.00	entry-duration deviation and log-duration
Previous-duration outlier	previous-duration features available	z=8.5; log +0.50	z=12; log +1.00	z=20; log +2.00	previous-duration deviation and log-duration
Semantic rare pair	base rule only	count=20	count=5; vehicle type=255	count=0; vehicle type=__UNK__	disagreement=True; pair count/rarity updated; severe also OOD=True
Rare missing pattern	base rule only	count=20	count=5	count=0	payable/net group missing; affected sums/residuals/counts missing; pattern rarity updated
Parser corruption stress	base rule only	spread=1	spread=2	spread=3	parse-error=True; group-count spread altered

Note: All rule-aligned bases satisfied weak_anomaly_probability ≤ 0.10 and the joint normal anchor. Within each analytical role and mechanism, 2,000 bases were selected deterministically using seed 20221213. Each base generated matched control and intervention records at all three severity levels. Binary validity interventions retained identical transformations across severity labels.

Panel B

Rule-orthogonal mechanism	Mild	Moderate	Severe	Principal intervention
Contextual fee shift	z=4.5; log +0.10	z=6.0; log +0.30	z=7.5; log +0.60	payable/net contextual deviations
Entry-duration subthreshold	z=4.5; log +0.20	z=6.0; log +0.50	z=7.5; log +0.80	entry-duration features
Previous-duration subthreshold	z=4.5; log +0.20	z=6.0; log +0.50	z=7.5; log +0.80	previous-duration features
Semantic mid-frequency	pair count=999	pair count=200	pair count=21	disagreement=True and pair rarity updated
Pattern mid-frequency	pattern count=9999	pattern count=500	pattern count=21	payable-group unavailable and pattern rarity updated
Multiview subthreshold	z=4.5; pair=500; pattern=5000	z=6.0; pair=100; pattern=200	z=7.5; pair=21; pattern=21	simultaneous contextual-fee, entry-duration, semantic, and pattern-frequency shifts

Note: Before intervention, both members of each rule-orthogonal pair were normalized to the same non-anchor ambiguous state using pair_train_count=999, pair_rarity_score=log[(Ntrain+1)/1000], and vehicle_type_disagreement=False. Each role/mechanism used 1,500 deterministic bases with seed 20221213. Post-construction verification required zero primary anomaly votes and an inactive joint normal anchor for every control and intervention record.

Panel C

Target intervention prevalence	Total records	Intervention records	Control records	Threshold
1%	100,000	1,000	99,000	Fixed validation-selected threshold
3%	100,000	3,000	97,000	Fixed validation-selected threshold
5%	100,000	5,000	95,000	Fixed validation-selected threshold

Note: Records were sampled separately from the test_joint rule-aligned intervention and control pools using deterministic random states derived from seed 20221213. Replacement was used only when the requested sample size exceeded the available pool.

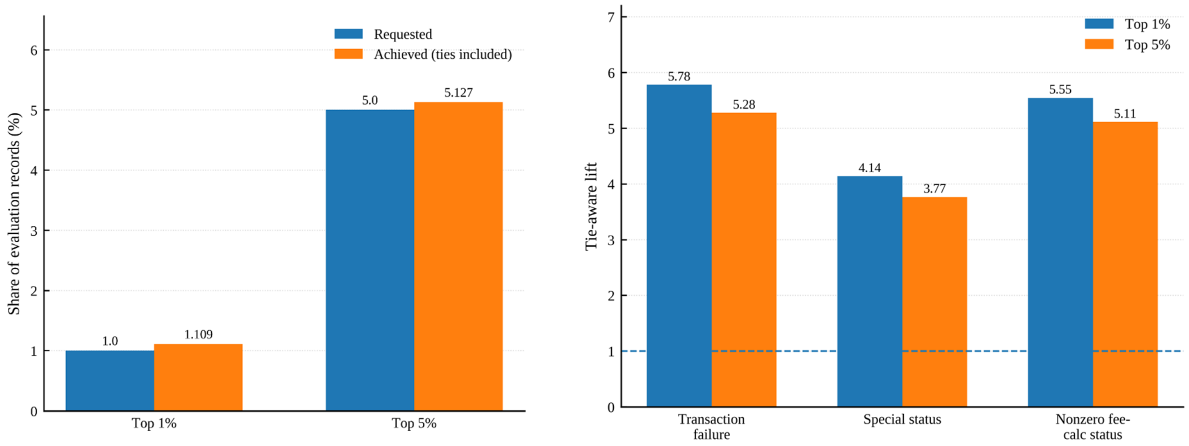

(**a**) (**b**)

Figure S1. Tie-aware high-score enrichment in the joint-transfer subset: (a) requested and achieved high-score fractions after retaining all records tied at the selection cutoff; and (b) enrichment lifts for transaction failure, special-transaction status, and nonzero fee-calculation status at the inclusive Top-1% and Top-5% cutoffs.
